# AI-driven nanomedicine for cancer theranostics

**DOI:** 10.1186/s12943-025-02563-9

**Published:** 2026-02-13

**Authors:** Ashutosh Tiwari, Widodo, Dyah Ika Krisnawati, Chih-Yu Chen, Tsung-Rong Kuo

**Affiliations:** 1 International Ph.D. Program in Biomedical Engineering, College of Biomedical Engineering, Taipei Medical University, Taipei, 11031 Taiwan; 2 Institut Teknologi Al-Mahrusiyah, Kediri, 64112 East Java Indonesia; 3 Department of Nursing, Faculty of Nursing and Midwifery, Universitas Nahdlatul Ulama Surabaya, Surabaya, 60237 Indonesia; 4 Center for Continuing Care Research (C3R), Universitas Nahdlatul Ulama Surabaya, Surabaya, 60237 Indonesia; 5 Department of Orthopedics, Shuang Ho Hospital, Taipei Medical University, 291, Zhongzheng Road, Zhonghe District, New Taipei City, 23561 Taiwan; 6 School of Biomedical Engineering, College of Biomedical Engineering, Taipei Medical University, Taipei, 11031 Taiwan; 7 Graduate Institute of Nanomedicine and Medical Engineering, College of Biomedical Engineering, Taipei Medical University, Taipei, 11031 Taiwan; 8 Precision Medicine and Translational Cancer Research Center, Taipei Medical University Hospital, Taipei, 11031 Taiwan

**Keywords:** AI-driven nanomedicine, Cancer theranostics, Smart nanoparticle, Inverse design, Precision oncology, Nanotoxicity

## Abstract

Nanomedicine's merging with artificial intelligence (AI) is fundamentally changing cancer theranostics through precise creation of multifunctional nanoparticles which can simultaneously diagnose and treat diseases. Traditional cancer treatments today face issues with imprecise delivery and general body toxicity as well as late-stage disease recognition which theranostic nanoplatforms address through precision drug transport and live imaging functions. In this analysis, we examine the existing state and forthcoming developments of AI applications in cancer theranostics through nanoparticles. Our study first examines the three primary classes of theranostic nanomaterials that show clinical significance: liposomes, gold nanoparticles, iron oxide nanoparticles, and quantum dots. AI is being applied to nanoparticle development through machine learning, deep learning, reinforcement learning, and generative models that support physicochemical predictions, synthesis optimization, biodistribution modeling, and inverse design. We analyze clinical applications of AI solutions which support patient identification, response predictions, and implementation of virtual patient models for individualized cancer treatment. The paper evaluates major difficulties which are nanotoxicity, AI explainability, data limitations, and regulatory concerns while addressing ethical dilemmas. Rather than a broad overview of nanomedicine, we center on AI methods that directly improve theranostic decisions and support this with worked exemplars reporting datasets, baselines, metrics, and clinical tie-ins. This Task–Data–Method–Metric lens replaces generic background and grounds claims in reproducible evidence.

**Graphical abstract:**

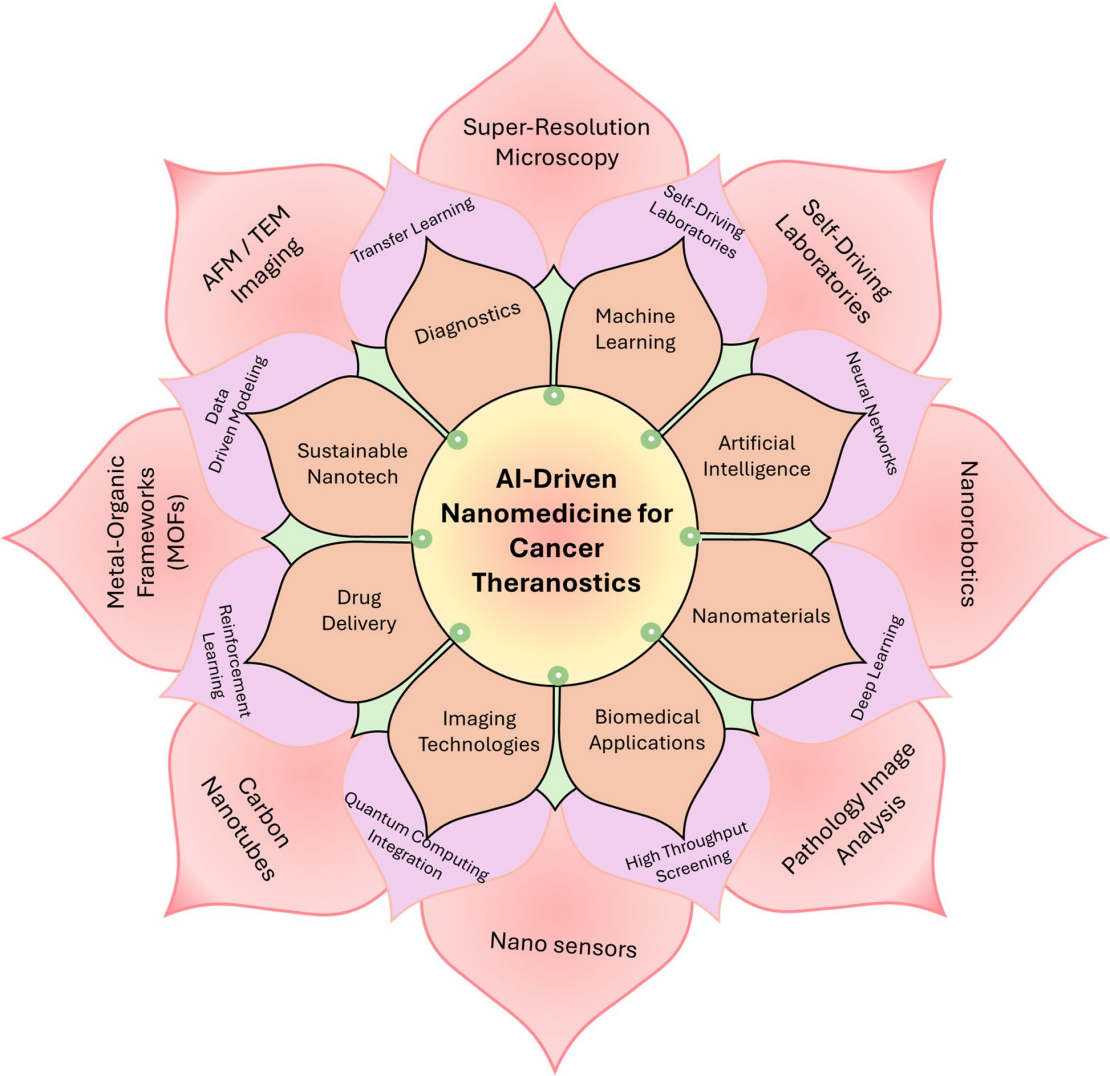

**Supplementary Information:**

The online version contains supplementary material available at 10.1186/s12943-025-02563-9.

## Introduction: rethinking cancer management at the nanoscale

Cancer imposes a severe strain on global healthcare systems with about 10 million annual deaths recorded in 2020 and future projections of a 60% increase in the incidence during the next 20 years [1]. Existing systemic chemotherapy and radiotherapy as well as immunotherapy treatments have experienced significant advancements, but overall survival outcomes for most solid tumors have remained poor in cases of advanced-stage diagnoses, because current treatments produce limited and non-specific benefits [2, 3]. Cancer control fails most often at the point where biology outpaces our timing. Intratumoral heterogeneity, rapid evolutionary escape, and spatially diverse tumor niches mean that the same patient can host multiple disease states, some sensitive, others already resistant. Conventional pathways still deliver average therapies to changing tumors, and diagnostic signals arrive too slowly to prevent overtreatment in some and undertreatment in others [4, 5]. Standard systemic chemotherapy cancer treatment faces major problems because treatments display numerous drawbacks, including poor delivery to target sites, random distributions across the body, and harmful effects on normal cells. Implementing radiotherapy can damage healthy tissues because treatments cannot be precisely targeted to specific anatomical areas [6–8]. These two treatment modalities have limitations because they do not possess the capabilities to track tumor evolution or detect which specific disease states are responding to treatment and which are becoming resistant. The clinical management of tumors is difficult because existing treatments do not work effectively due to complex variations in both tumor spatial distributions and molecular compositions [9, 10].

The medical field transitioned into a new dimension through nanomedicine which enabled solutions to currently faced difficulties. The distinctive physicochemical characteristics of nanomaterials, including their high surface area-to-volume ratio and surface tunability with enhanced permeability and retention (EPR) effects, enable nanoparticle (NP)-based therapeutic delivery with improved accuracy and decreased system-wide harm [11, 12]. Combining diagnostic capabilities with these platforms has established theranostics as a revolutionary system in which cancer imaging and monitoring are performed alongside therapeutic functions [13]. The use of theranostic NPs makes it possible to follow changes in tumor responses in real time, while delivering therapy according to molecular biomarkers and establishing novel approaches for personalized oncology. However, the elaborate design requirements for NP development make it difficult to convert theoretical theranostic systems into real-world applications. The process of optimization for material composition, size, shape, surface charge, ligand density, drug loading, release kinetics, and biological interaction profiles must take place simultaneously [14, 15]. Conventional methods of creating NP designs through experimentation require extensive human labor combined with low-speed processes which lead to inconsistent results in patient care [16, 17].

Theranostic nanomedicine has received a significant boost from artificial intelligence (AI) together with machine learning (ML) and deep learning (DL) technologies [18–21]. AI algorithms can be used to analyze large datasets to discover relationships between NP-biological interactions which support rational design through nonlinear models of physicochemical properties and therapeutic efficacies [4]. Medical researchers now implement ML methodologies to predict NP biodistributions and toxicity, while deep generative models help design materials and convoluted neural networks (CNNs) have optimized NP image analyses. AI applications have demonstrated potential for patient stratification together with therapy response predictions and adaptive treatment regimen development through real-time biomarker monitoring [18, 22]. The current moment in precision oncology presents an optimal environment for AI-nanomedicine integration because medical decisions now depend on multi-omics data, digital pathology, and dynamic imaging. Through the combination of these three data types with intelligent NP systems, AI enables cancer management strategies which can adapt to disease evolution through real-time feedback. Standard population-based treatment protocols are now evolving into autonomous personalized nanoscale medical interventions [23, 24].

In this paper, we extensively outline recent AI-based advancements in nanotheranostics through a detailed examination of material developments and their transition to medical practice. The research begins with a detailed description of basic nanomaterials that serve as the foundation for theranostic systems, including gold NPs (AuNPs), liposomes, quantum dots, (QDs) and magnetic nanostructures because their adjustable physicochemical attributes enable dual diagnostic and therapeutic functions. The paper continues by explaining how AI optimizes design processes through its prediction of NP behavior, synthesis parameter optimization, biodistribution modeling, drug release time control, and multifunctional platform inverse design. The paper presents comprehensive evaluations of translational applications by examining clinical case studies alongside AI-based patient stratification techniques and digital twin frameworks for personalized therapy response simulations. Prior articles summarize the promise of ML for nanotheranostics or focus on cancer-type–specific theranostics (e.g., TNBC) without a decision framework that ties methods to clinical endpoints. In contrast to previous reviews, our review contributes: (i) a decision-centric TDMM framework mapping tasks to data, methods, and decision-relevant metrics (e.g., calibration and net-benefit) rather than accuracy alone; (ii) worked exemplars with datasets, baselines, and clinical tie-ins, including closed-loop inverse design via microfluidics/active learning, uncertainty-aware decision support, corona-aware fate prediction, and multimodal foundation models; (iii) an explicit calibration and uncertainty emphasis for safer thresholds at deployment; and (iv) a translation playbook that integrates SaMD-oriented regulation, ethics (GDPR/HIPAA), and a SWOT-grounded roadmap to prospective validation. Together, these move the field from descriptive surveys toward actionable, clinically anchored AI-nanotheranostic development.

**Fig. 1:**
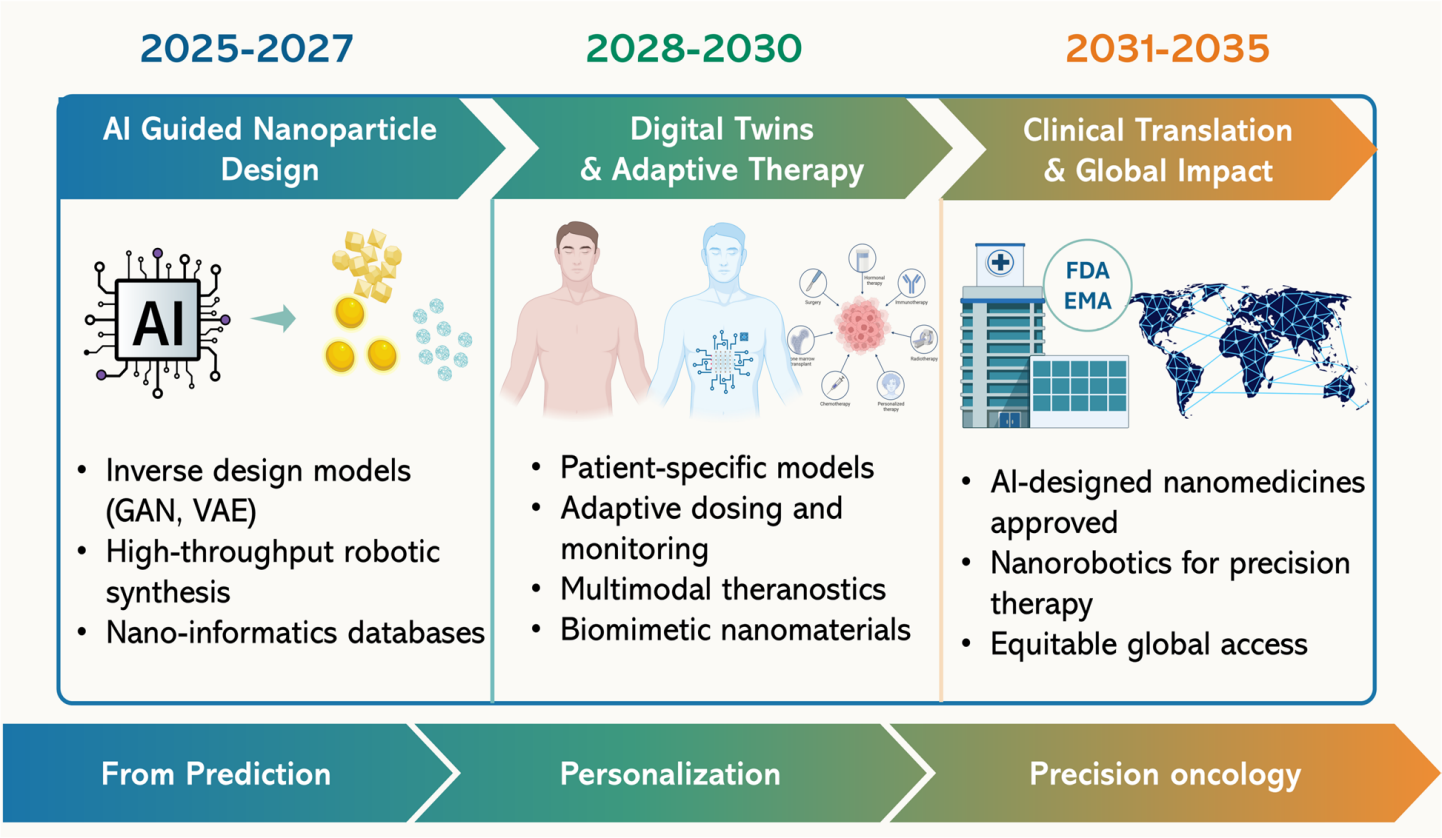
2025–2035 roadmap for AI-integrated nanomedicine. The progression highlights three phases: AI-guided nanoparticle design (2025–2027) with generative modeling and robotic synthesis, digital twins and adaptive therapy (2028–2030) for personalized monitoring and multimodal theranostics, and clinical translation and global impact (2031–2035) featuring regulatory approval of AI-designed nanomedicines, nanorobotics for precision therapy, and equitable global access

To contextualize these developments, Fig. 1 presents a 2025–2035 roadmap highlighting the projected evolution of AI-integrated nanotheranostics. The first phase (2025–2027) emphasizes AI-guided nanoparticle design, where generative algorithms and robotic synthesis platforms accelerate material optimization and populate standardized nano-informatics resources. The second phase (2028–2030) advances toward digital twins and adaptive therapy, integrating patient-specific models with real-time monitoring, multimodal imaging, and biomimetic nanoplatforms for personalized interventions. The final phase (2031–2035) envisions clinical translation and global impact, where the first AI-designed nanomedicines receive regulatory approval, nanorobotics enable precision drug delivery, and equitable access frameworks extend these innovations worldwide. Together, this trajectory illustrates a decadal vision in which prediction-driven design converges with personalization and precision oncology.

## Nanotheranostics: a platform for targeted cancer diagnosis and therapy

The field of cancer management has seen a groundbreaking transformation with the integration of diagnostics and therapeutics into nanoscale platforms known as nanotheranostics [25–27]. Diagnostic and therapeutic elements in conventional approaches exist separately in time and space while nanotheranostic systems combine real-time imaging with targeted drug delivery and dynamic therapeutic response monitoring in a single framework [28–32]. Advanced therapeutic systems as illustrated in Table 1, focus on tumor-specific characteristics, such as abnormal vascular permeability, acidic microenvironments, overexpressed receptors, and metabolic aberrations, to achieve precise therapeutic approaches [44, 45]. Chou et al. reported medians and 95% confidence intervals for the AI-predicted tumor kinetic parameters (KTRES_50, KTRES_max, KTRES_n, KTRES_rel), which closely matched the distributions of the original PBPK-derived parameters. At the delivery-efficiency level, the AI–PBPK model achieved higher adjusted R^2^ values for DE24 and DEmax (0.83 and 0.82, respectively) than the earlier PBPK-based delivery-efficiency model (0.62 and 0.81), with a greater fraction of predictions within 2- and threefold error, indicating that the AI-predicted parameters reduced systematic bias in short-term tumor delivery [33].

**Table 1:** AI-driven nanotheranostic exemplars

Exemplar	Task	Data	Method & Baseline	Metrics	Outcome	Clinical tie-in	Key refs
Hybrid PBPK–ML for NP tumor delivery (dose planning)	Predict organ/tumor deposition from physicochemical features to inform dosing	Nano-Tumor Database: NP features (Type, Size/HD 5–456 nm, Zeta magnitude/Charge, Shape, MAT), therapy descriptors (TS, TM, CT, TW, TSiz), data-driven PBPK params (KTRES_rel, KTRES_n, KTRES_max, KTRES_50), tumor delivery efficiency (DE24, DE168, DEmax), dose regimen (Dose, BW, AR); multiple tumor models and cancer types	AI-QSAR model (LR, SVR, RF, XGBoost, LightGBM, DNN) trained on 288 tumor datasets from the Nano-Tumor Database to predict four tumor-related PBPK kinetic parameters (KTRES_max, KTRES_50, KTRES_rel, KTRES_n). The best-performing DNN is integrated with a whole-body PBPK model for tumor-bearing mice comprising plasma, lungs, liver, kidneys, spleen, brain, muscle, remaining tissues, and a tumor compartment; except for plasma and brain, each tissue/tumor is subdivided into capillary blood, interstitium, and endocytic/tumor cells. AI-predicted parameters are constrained within physiologically plausible ranges derived from prior PBPK calibration rather than formal Bayesian priors and are passed into the PBPK ODE system implemented in R (mrgsolve, FME) to simulate DE24, DE168, DEmax, and tumor pharmacokinetic profiles; the AI-QSAR component is implemented in Python (scikit-learn, TensorFlow/Keras, Hyperas)	DNN test R^2^/RMSE (tumor parameters): KTRES_rel 0.47/1.85; KTRES_n 0.53/1.60; KTRES_max 0.91/0.71; KTRES_50 0.23/32.15. Delivery efficiency vs data-driven PBPK: Adj-R^2^ 0.83 (DE24), 0.56 (DE168), 0.82 (DEmax); % within twofold error: 69.7%, 11%, 74.6%; % within threefold error: 92%, 19%, 92%. Tumor PK profiles: Adj-R^2^ 0.67; 57% within twofold and 87% within threefold error; R^2^ ≥ 0.70 in 133/288 datasets	AI-assisted PBPK improves short-term tumor delivery prediction relative to the authors’ previous PBPK-based delivery-efficiency model and reproduces measured tumor pharmacokinetics across diverse NPs and tumor types	Enables cross-NP prioritization and model-informed selection of dose and schedule for preclinical screening; provides a template for future AI-assisted PBPK tools to support early-phase dose planning	[33]
Protein corona prediction to uptake/PK (safety & targeting)	Predict corona composition from NP features to anticipate uptake/off-target effects	17 DNA nanostructures (tetrahedron Th/Th-Ch, box Bx, squares Sq1/Sq2/Sq3 ± aptamers/biotin, tube Tu, rod Rd) with/without PLL-PEG (@PL) coating; pooled human serum incubation; negative-control magnetic beads; SDS-PAGE to in-gel/in-solution digestion to nanoUHPLC–MS/MS on high-resolution Orbitrap platform; database search with target–decoy strategy, FDR-controlled, peptide-filtered label-free UHPLC-MS/MS (timsTOF Pro 2 + Evosep One) with triplicate nanostructure samples; spectral-count–based relative abundances vs diluted serum, magnetic bead controls, and log2 fold-changes plus t-tests for enrichment; GO-term enrichment reported at FDR ≤ 0.05	XGBoost + SHAP for two binary tasks: (i) present vs absent in corona (including enriched, depleted, and uniquely present proteins), (ii) enriched vs depleted relative to serum (using MB-subtracted log2 fold-changes).Comparators: Random Forest and Gradient Boosting (inferior); heuristic baselines (size/charge rules). Protocols: 10-split evaluation on pooled data and leave-one-nanostructure-out generalization	Pooled-data performance: AUC 0.97, 92% accuracy (present/absent); AUC 0.96, 91% accuracy (enriched/depleted). Held-out nanostructure generalization: AUC 0.95, 88% accuracy (present/absent); AUC 0.94, 89% accuracy (enriched/depleted). Reliability: Predicted in-corona probabilities differ significantly by outcome: TP > FP and TN < FN (p < 0.01), indicating probabilities can serve as a confidence/reliability measure	Design insights: non-DNA modifications (PLL-PEG, cholesterol) and ζ-potential dominate adsorption; shape has limited effect across tested forms. Polymer coating produces distinct corona clusters; complement components (e.g., C3/C4, C1q) and immunoglobulin subclasses are differentially enriched across designs, allowing models to estimate patterns of complement activation and Fcγ-receptor engagement. SHAP analysis highlights interpretable levers (e.g., exposed residue types, flexibility, surface charge) and shows that immunogenic/opsonic vs dysopsonic (albumin/apolipoprotein-rich) coronas can be partially disentangled from NP design features	Prospective pre-screen to steer uptake/PK and immune interactions before in vivo work; informs stealth vs targeting trade-offs, corona engineering (e.g., PLL-PEG, cholesterol) and downstream PBPK priors; enables early flagging of designs predicted to drive strong complement activation or Fc-mediated clearance; reduces failed candidates and supports safer, “design-for-translation” decisions rather than patient-level predictions	[34, 35]
PA/US imaging + deep learning for response assessment	Non-invasive prediction of luminal vs non-luminal breast cancer subtypes from PA/US imaging	n = 388 patients (train 271; test 117, 7:3 split). Baseline characteristics largely balanced (only age differed, p = 0.004; all other variables p > 0.05). Features: 3,609 total (1,561 radiomics; 2,048 DL). After screening and Z-score normalization to 3,599; Pearson filtering retained 2,453; LASSO selected 9 non-zero features (3 radiomics, mainly GLSZM/GLCM; 6 DL from ResNet50)	Integrated DLRN model = Clinical predictors (BMI, Menopause, Posterior features) + DLR features in logistic regression. Baselines: Clinical-only, Radiomics-only (Rad), DL-only, DLR-only. RF/ET/XGB showed overfitting; LR chosen for DLR due to best generalization	Test AUC (95% CI): DLRN 0.924 (0.877–0.972); DLR 0.847 (0.758–0.936, p = 0.026 vs DLRN); DL 0.822 (0.725–0.919, p = 0.06); Rad 0.717 (0.597–0.838, p < 0.001); Clinical 0.820 (0.745–0.895, p = 0.002). Accuracy (test): 0.809. Sensitivity: 0.960 (test). NPV: 0.951–0.986; PPV: ≈0.519–0.533. Calibration: good (Hosmer–Lemeshow, calibration curves). Decision curve: highest net benefit for DLRN	The DLRN integrated model significantly outperforms all single-modality baselines with strong generalization and calibration on the test set	Feasible preoperative subtype stratification from PA/US alone, supporting patient selection and potential tailoring of nano-theranostic strategies (agent choice; dose/energy adjustments) without biopsy delays	[36]
Learning biodistribution/tumor delivery directly from data	Predict delivery efficiency (DE, %ID at 24 h post-IV) to tumor and major organs from NP descriptors	Nano-Tumor Database (latest version). Records after cleaning: tumor n = 403, heart n = 252, liver n = 341, spleen n = 312, lung n = 274, kidney n = 298. Inputs: NP type, MAT/core material, shape, size (HD), zeta, TS (targeting), TM (tumor model), CT (cancer type), dose variables; outputs: DE to each tissue	Model comparison: LR, SVR, RF, XGBoost, LightGBM, DNN; fivefold CV + held-out test. Best: DNN. Baselines: linear and tree ensembles	DNN (test set R^2^/RMSE): DETumor 0.41/2.02, DEHeart 0.42/0.69, DELiver 0.45/7.89, DESpleen 0.79/1.27, DELung 0.87/0.35, DEKidney 0.83/0.85. DNN (fivefold CV R^2^ mean ± SD): tumor 0.39 ± 0.28, heart 0.52 ± 0.20, liver 0.55 ± 0.12, spleen 0.64 ± 0.17, lung 0.90 ± 0.04, kidney 0.74 ± 0.09; CV RMSE similar to test. Traditional regression check (Adj-R^2^, observed vs DNN-predicted): tumor 0.62, heart 0.51, liver 0.71, spleen 0.85, lung 0.87, kidney 0.79. XGBoost (test): R^2^ 0.08–0.41, RMSE 0.27–9.58 across tissues; RF similar for several organs; LR/SVR weak	DNN outperformed all ML baselines, yielding reliable, reproducible predictions across tissues; SHAP analysis: MAT (core material) most important physicochemical driver, CT most important tumor-strategy feature. Virtual screening: 7,000 synthetic formulations (7 MATs) scored; 377 predicted with DETumor 3–4%ID and tissue DEs above BLQ	Rapid in-silico triage of NP designs and dose/schedule hypothesis generation for theranostic trials; informs PBPK priors and material/targeting choices before animal work	[37]
Integrating clinical variables, radiomics, and tumor-derived cfDNA for outcome prediction in resectable esophageal adenocarcinoma	Improve prediction of overall survival (OS), time to progression (TTP), and pathologic complete response (pCR) by combining clinical variables with radiomics and cfDNA	n = 111 stage II–III rEAC from 2 centers (Amsterdam UMC, UMC Utrecht). Imaging: baseline & restaging CT (n = 111/109), restaging [18F]FDG-PET (n = 105; baseline PET n = 61 not used for radiomics). Radiomics: 105 PyRadiomics features per scan; ComBat harmonization (manufacturer, slice thickness). cfDNA (baseline): fragmentomics (P20–150, FES), ichorCNA, mutation panel; tumor-agnostic. SOURCE clinical predictor included	Elastic-net models with fivefold internal–external CV (Cox for OS/TTP; logistic for pCR); 500 × bootstrap CIs; redundancy filter for correlated features. Baselines: SOURCE alone; combinations with baseline CT, restaging CT/PET, and cfDNA	OS (C-index, CV): SOURCE 0.45; + baseline CT 0.54; + cfDNA 0.55; + restaging PET 0.65; + restaging PET + cfDNA 0.62. TTP (C-index, CV): SOURCE 0.44; + baseline CT 0.55; + cfDNA 0.59; + restaging PET 0.60; + restaging PET + cfDNA 0.59. pCR (AUC, CV): SOURCE 0.47; + baseline CT 0.61; + cfDNA 0.48; + baseline CT + cfDNA 0.61; + restaging CT 0.49; + restaging PET 0.42; + restaging CT/PET + cfDNA ≤ 0.50. Risk stratification: Baseline models split high- vs low-risk (OS log-rank p = 0.0017 for SOURCE + rad and SOURCE + rad + cfDNA; TTP best separation p = 0.0001 for SOURCE + rad + cfDNA)	Adding radiomics or cfDNA improves discrimination over SOURCE alone (largest gain for restaging PET on OS/TTP; modest pCR gains with baseline CT radiomics). Models are exploratory; external validation needed before clinical use	Template for multimodal triage: select candidates and timing for therapy intensification/de-escalation and (by extension) for nano-theranostic agent use; informs trial enrichment and monitoring strategies	[38]
Data-driven 3D dose calculation via deep learning	Fast, accurate 3D dose computation to support iterative planning/kinetic modeling workflows (e.g., nano-tracers, radionuclide or RT theranostics)	IMRT cases across 4 sites (nasopharynx, lung, rectum, breast): 267 pts total to 200 train, 20 val, 47 test; per-patient ~ 10 beams; inputs: CT volumes + fluence maps (converted to 3D)	3D U-Net/ResNet with long skip connections; inputs = CT + fluence-map–converted volume (FMCV) generated by 3D-DDA ray traversal (includes inverse-square law); ReLU, Adam (MSE), dropout + augmentation; batch = 2, ~ 200 epochs on 2080Ti (12 GB). Baseline/“ground truth”: TPS (collapsed-cone convolution) dose	Per-voxel bias vs TPS (normalized to Rx): 0.17% ± 2.28% across 47 test pts. Speed: “less than several seconds per beam.” DVH/clinical indices: organ- and target-level means SD closely match TPS; two-sample t-tests typically non-significant across indices (e.g., brainstem Dmax p = 0.05; spinal cord Dmax p = 0.06; many p ≫ 0.05 up to 0.99)	DL dose closely reproduces TPS distributions across disease sites with minimal bias and tight variance; DVHs/indices concordant with TPS, demonstrating feasibility and reliability of data-driven dose calculation	Enables near-real-time plan iteration, adaptive RT, and integration with dynamic PET nano-tracer kinetic modeling (rapid forward dose estimates during parameter sweeps); pathway to MR-only and proton implementations for nano-theranostic trials	[39]
DNN for Ag-nanoprism synthesis	Optimize nano-synthesis conditions to match a target absorbance spectrum (λ_peak ≈ 645 nm) and geometry (triangular prisms)	Closed-loop droplet microfluidics HTE; per run: 15 chemical conditions × 20 droplet replicas; variables: flow rates of AgNO₃ (), seeds (Q_seed), trisodium citrate (Q_TSC), PVA (Q_PVA), and total flow (Q_total); TEM for size/shape; UV–Vis full spectra	Two-step framework: Step-1 BO with GP surrogate + Local Penalization (batch) on sparse data; random sampling (RS) used as control; dynamic expansion of parameter space at runs 4–5 to 6–8. Step-2 DNN regression trained on BO data; DNN samples via grid search; SHAP for feature importance. Baselines: RS and DNN-only grid search	BO vs RS: BO overtakes RS by run 3 (loss declines faster). Parameter-expansion triggers further loss drop. DNN best performer at run 8 shows significantly lower median loss than BO. Shape vs amplitude correlations: runs 1–5	DNN outperformed BO after expansion, achieving lower loss and accurate spectral predictions; TEM confirmed nanoprisms narrowing toward ~ 65 nm edge (≈13 nm thick); SHAP ranked Q_{AgNO3} and Q_{seed} most influential; DNN avoided BO surrogate artifacts on Q_{total}	Rapid convergence to clinically viable nano-agents	[40]
Active targeting ligand design (GNN/transformers)	Prioritize receptor–ligand pairs (small molecules/peptides as nanoparticle ligands) by predicting binding affinity to tumor-overexpressed targets to guide active-targeting functionalization	Davis: 30,056 pKd values (68 drugs, 442 targets; 5.0–10.8). KIBA: 118,254 affinity scores (2,111 drugs, 229 targets; 0–17.2). Drug molecular graphs via RDKit; protein contact maps (PconsC4); DTI bipartite network; similarity matrices (PubChem clustering; Smith–Waterman). Six-fold split (5 train/1 test)	CSCo-DTA: cross-scale graph contrastive learning. GCN encoders for molecule-scale (drug graph; protein contact map) + network-scale (drug–target graph); InfoNCE across scales; MLP head; multi-task loss (MSE + contrastive). Baselines: DeepDTA, GraphDTA, DGraphDTA, MATT_DTI (attention), FusionDTA, and a concat ablation	Davis: MSE 0.166, r^2^m 0.776 (vs DGraphDTA 0.221/0.687; FusionDTA 0.208/0.749). KIBA: MSE 0.127, r^2^m 0.808 (vs DGraphDTA 0.131/0.797; FusionDTA 0.130/0.791)	Outperformed all baselines on both datasets; ablations showed both scales and the contrastive module are required. Case study: predicted a novel Erlotinib target (IRAK4); docking score 118.115 vs EGFR 122.603; 9/10 top predictions matched experimental activity ranks	Use predicted high-affinity ligand–receptor pairs to select/engineer targeting moieties (e.g., peptides/aptamers/SMALL-molecule ligands) for nanoparticle surface modification, narrowing wet-lab screens and improving on-target accumulation before in vivo testing	[41]
Nanotoxicity prediction & safe-by-design	Predict NP-induced cellular toxicity to enable safe-by-design screening	n = 244 records (NanoHUB repository) across metallic, metal-oxide, polymeric, silica NPs; features: core size, shape, coating, ζ-potential, surface area; exposure dose/duration; tissue, cell type/line; binary outcome: “Triggered” vs “No effect.” KNN imputation for missing (k = 3)	Five ML classifiers with tenfold CV and threefold inner CV for tuning: Random Forest (RF), Decision Tree (DT), SVM, Naïve Bayes (NB), ANN; Gini index for feature importance. Baseline: DT, SVM, NB, ANN	RF (best): Accuracy 93.45% ± 3.39, AUC 0.966 ± 0.027, F1 92.44% ± 3.85, Sensitivity 92.70% ± 8.20, Specificity 94.18% ± 5.67, Error 6.55% ± 3.39. DT: Accuracy 90.57% ± 5.52, AUC 0.817 ± 0.178, F1 89.31% ± 6.15, Sens 90.70% ± 7.51, Spec 90.55% ± 6.86. (SVM/NB/ANN lower.)	RF outperformed all comparators on this dataset; Gini analysis highlighted cell line, exposure dose, and tissue as the most influential factors; exposure duration, shape, and cell type had least effect. Authors recommend RF for NP toxicity prediction to save cost/time vs. wet-lab assays	Front-end hazard triage to down-select safer nano-formulations before in vivo work; supports safe-by-design and reduces animal studies; plugs into theranostic pipelines to de-risk candidate agents prior to efficacy testing	[42]
Active learning for cell-specific uptake optimization (microfluidics + HCI + Bayesian ML)	Maximize PLGA-PEG nanoparticle uptake in MDA-MB-468 human breast cancer cells under quality constraints (size/PDI)	Three iterative cycles: Cycle-0 DoE (n = 29 formulations), Cycle-1 exploration (n = 10), Cycle-2 exploitation (n = 10); additional validation sets (5 high-predicted vs 5 low-predicted). High-content imaging (10 FOV/condition; nuclei/membrane/NP channels) to quantify per-cell fluorescence as fold-change vs bulk 100% PLGA-PEG control. Design variables: PLGA, PLGA-PEG, PLGA-PEG-COOH, PLGA-PEG-NH2 ratios; solvent/antisolvent FRR. Virtual library: 100,000 candidates. PDI filter < 0.20; DLS for size/PDI	Closed-loop active learning: Bayesian neural network (SVI; 3 hidden layers, ReLU; 500 MC draws for predictive uncertainty) for uptake; XGBoost models for size/PDI; Cycle-1 uncertainty-driven exploration (k-means diversity on top-uncertainty set), Cycle-2 exploitation (highest predicted uptake with low uncertainty). Baseline: initial DoE/random screen; alternatives assessed (RF, XGBoost, Gaussian Processes)	Uptake (fold vs control): Cycle-0 mean 2.03 ± 1.28 (range 0.40–4.77); Cycle-1 mean 9.72 ± 2.70 (5.72–14.40); Cycle-2 mean 12.30 ± 2.02 (8.60–14.50). Validation split: high-predicted 10.54 ± 0.66 vs low-predicted 2.74 ± 0.99; p < 0.001. Size/PDI (validation): high-uptake 114.0 ± 5.2 nm, PDI 0.122 ± 0.015; low-uptake 154.5 ± 21.4 nm, PDI 0.059 ± 0.013. Cycle time ≈ 1 week/iteration	Closed-loop platform tripled best uptake (≈ 5 × to ≈ 15 ×) in two ML-guided iterations; model uncovered composition levers (low PLGA fraction ≈ 5% and higher PLGA-PEG-COOH/NH2 ≈ 25% each associated with high uptake) and reliably separates high vs low performers	Rapid, unbiased lead optimization for cell-specific delivery, reducing experimental screens and enabling fast formulation lock for nano-theranostic agents; extendable to organoids/OOAC and endpoints beyond uptake (e.g., endosomal escape, cytotoxicity)	[43]

*%ID* Percent injected dose, *3D-DDA* Three-dimensional dose deposition algorithm, *AUC* Area under the ROC curve, *ANN* Artificial neural network, *AR* Administration route, *BLQ* Below limit of quantification, *BO* Bayesian optimization, *BMI* Body mass index, *BW* Body weight, *Bx* Box-shaped DNA nanostructure, *cfDNA* cell-free DNA, *CT* Cancer type (Nano-Tumor Database), *CV* Cross-validation, *DCR* Decision-curve analysis, *DE* Delivery efficiency, *DL* Deep learning, *DLRN* Deep learning + radiomics + clinical model, *DLS* Dynamic light scattering, *DNN* Deep neural network, *DT* Decision tree, *DVH* Dose–volume histogram, *EAC/rEAC* (resectable) esophageal adenocarcinoma, *ET* Extra-trees classifier, *FES* Fragment end signature, *FMCV* Fluence-map–converted volume, *FP/FN* False positive/false negative, *FOV* Field of view, *GCN* Graph convolutional network, *GLCM* Gray-level co-occurrence matrix, *GLSZM* Gray-level size zone matrix, *GP* Gaussian process, *GNN* Graph neural network, *HD* Hydrodynamic diameter, *HCI* High-content imaging, *IMRT* Intensity-modulated radiotherapy, *IRAK4* Interleukin-1 receptor-associated kinase 4, *LC–MS/MS* Liquid chromatography tandem mass spectrometry, *LR* Logistic regression, *LASSO* Least absolute shrinkage and selection operator, *LightGBM* Light Gradient Boosting Machine, *MAT* Core material (Nano-Tumor Database), *MPS/RES* Mononuclear phagocyte system/reticuloendothelial system, *MS/MS* Tandem mass spectrometry, *MSE* Mean squared error, *NB* Naïve Bayes, *NP/NPs* nanoparticle/nanoparticles, *PA/PAI* Photoacoustic/photoacoustic imaging, *PBPK* Physiologically based pharmacokinetic model, *PCR/pCR* Pathologic complete response, *PconsC4* Protein contact map prediction tool, *PD* Pharmacodynamics, *PET* Positron emission tomography, *PLL-PEG* Poly-L-lysine–polyethylene glycol, *PK* Pharmacokinetics, *PL* PLL-PEG-coated DNA nanostructure, *PVA* Polyvinyl alcohol, *PDI* Polydispersity index, *Rad* Radiomics-only model, *RF* Random forest, *RMSE* Root mean square error, *ROC* Receiver operating characteristic, *RS* Random sampling, *RT* Radiotherapy, *SDS-PAGE* Sodium dodecyl sulfate–polyacrylamide gel electrophoresis, *SHAP* SHapley additive explanations, *Sq* square-shaped DNA nanostructure, *SVM* Support vector machine, *SVR* Support vector regression, *TCIA* The Cancer Imaging Archive, *TEM* Transmission electron microscopy, *Th* Tetrahedral DNA nanostructure, *TPS* Treatment planning system, *TSC* Trisodium citrate, *TS* Targeting strategy (Nano-Tumor Database), *TM* Tumor model (Nano-Tumor Database), *TSiz* tumor size (Nano-Tumor Database), *Tu* tube-like DNA nanostructure, *UHPLC-MS/MS* Ultra-high-performance liquid chromatography–mass spectrometry, *VIF* Variance inflation factor, *WSI* Whole-slide imaging, *XGBoost* Extreme Gradient Boosting

Within our TDMM framework, Table 1 focuses on the tasks and clinical objectives that nanotheranostic platforms aim to achieve tumor-selective drug delivery, imaging-guided treatment, and integrated monitoring, along with the underlying nanomaterial classes and their physicochemical bases. Here, the data are preclinical and clinical nanomedicine studies, pharmacokinetic measurements, and imaging readouts, the methods are primarily experimental and mechanistic (e.g., EPR exploitation, ligand targeting, hyperthermia, gene delivery); and the metrics are classical clinical endpoints (OS, PFS, response rate, toxicity) and imaging surrogates. This section therefore defines the therapeutic and diagnostic targets that later AI methods are meant to support, rather than focusing on AI algorithms themselves.

**Fig. 2:**
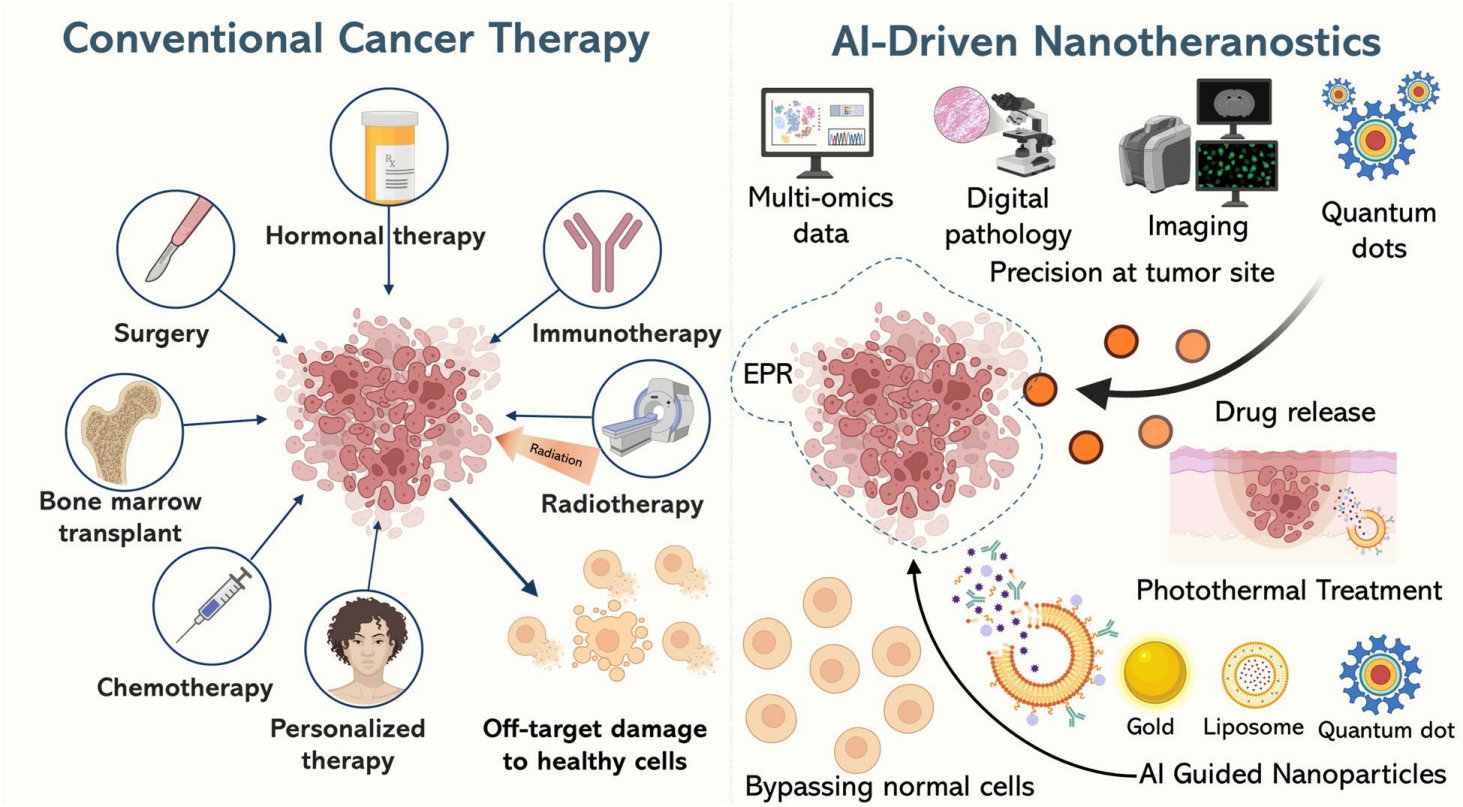
Conventional cancer therapy versus AI-driven nanotheranostics. Traditional chemotherapy and radiation lack specificity, causing off-target toxicity and damage to normal cells. In contrast, AI-guided nanotheranostics integrate multi-omics data, digital pathology, and engineered nanoparticles to bypass healthy tissues, enabling targeted drug release and photothermal therapy with precision at the tumor site

The fundamental principle of nanotheranostics depends on specially designed nanomaterials that combine diagnostic agents with therapeutic cargos for simultaneous use [46]. Implementation of size, shape, surface chemistry, and endogenous or exogenous stimulus responsiveness allows scientists to develop these nanostructures for multiple imaging functionalities [47] alongside controlled drug delivery and specific spatiotemporal therapy Fig. 2. The combination of factors leads to enhanced treatment results along with reduced body-wide damage while creating a continuous monitoring system that helps with precision cancer therapy [48, 49].

**Fig. 3:**
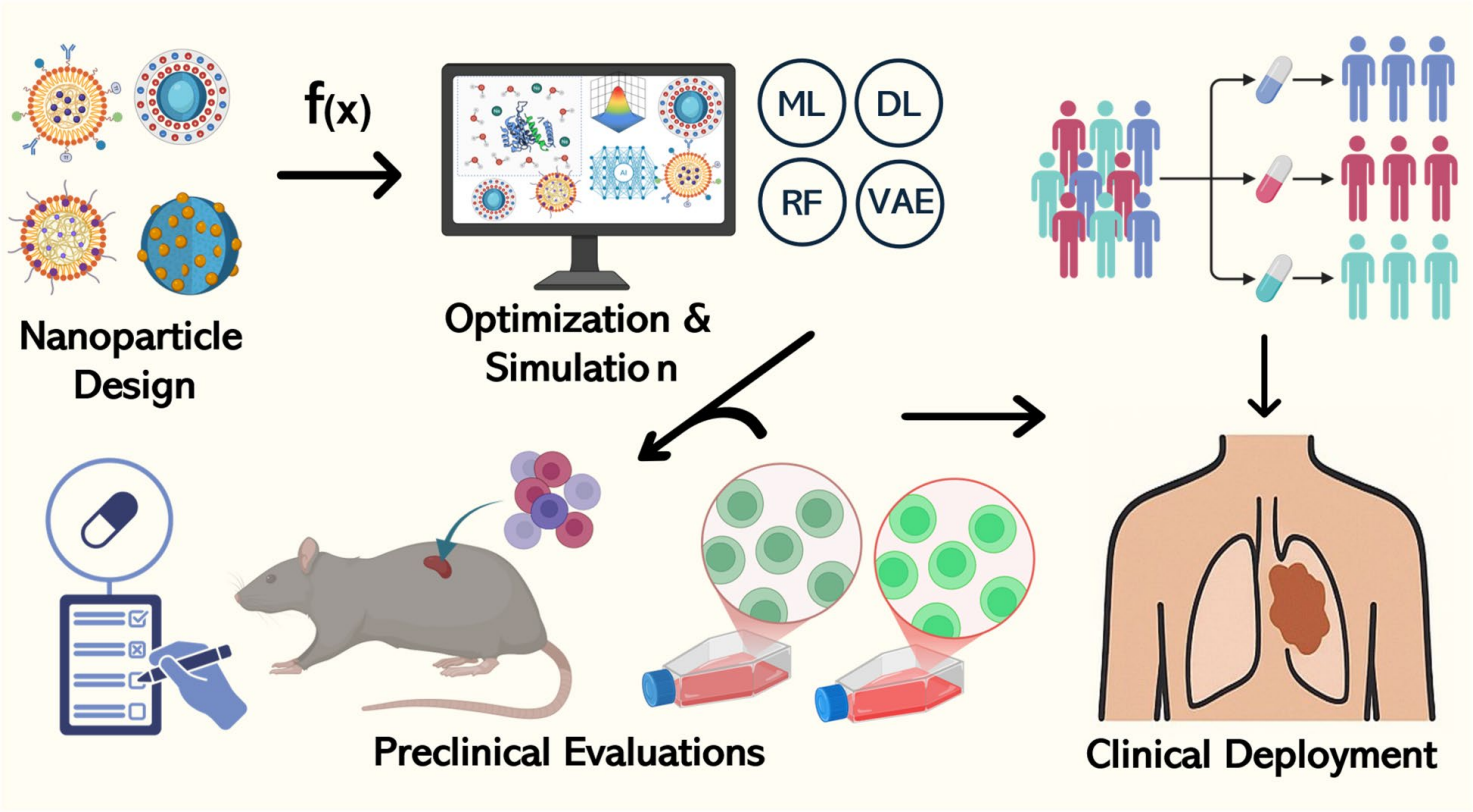
Simplified workflow of AI-driven nanoparticle design to clinical deployment. Nanoparticle designs are optimized through AI-based simulations (ML, DL, VAE, RL), validated in preclinical models, and advanced to clinical deployment for precision cancer therapy

Different types of NPs, such as AuNPs, liposomes, QDs, iron oxide nanocrystals, and polymeric carriers along with their distinctive physical and chemical features enable scientists to create nanotheranostic formulations that meet specific medical requirements [50–52]. Small modifications in NP design create substantial alterations in bioavailability alongside pharmacokinetics (PKs) and therapeutic effects which must be understood when developing complex systems [32]. The sophisticated development process Fig. 3 requires scientists to combine AI technology and ML with conventional methods because these modern tools deliver efficient high-throughput testing and data-based improvements which standard laboratory work cannot achieve [53, 54]. The following subsections explore essential categories of nanomaterials which researchers use in theranostic applications while examining the physical foundations of both therapeutic and diagnostic functions and the methods by which nanoscale characteristics enable specific cancer treatments.

### Types of nanomaterials in theranostics

**Fig. 4:**
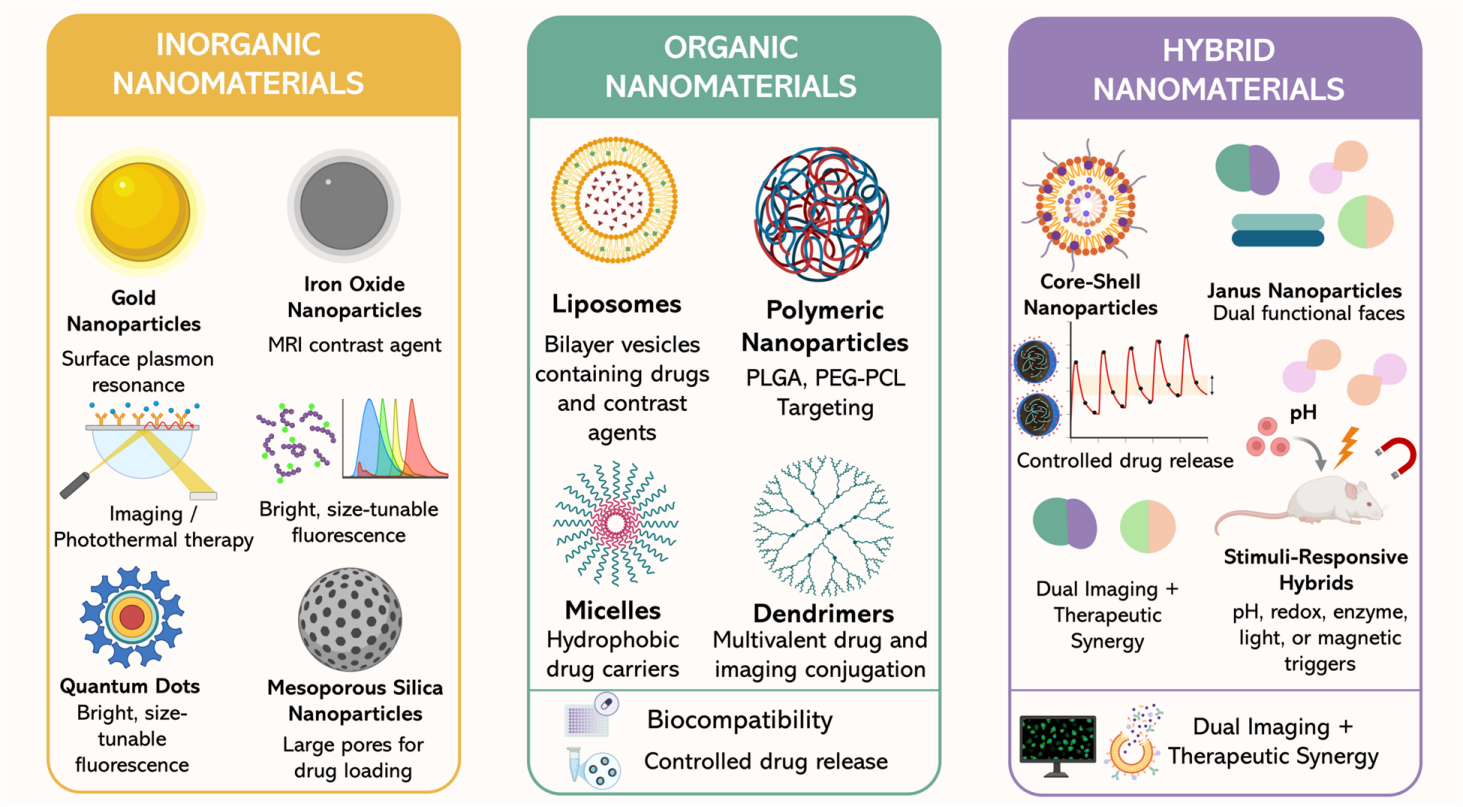
Classes of nanomaterials used in cancer theranostics. Inorganic, organic, and hybrid nanomaterials provide distinct therapeutic and diagnostic advantages. Inorganic platforms (gold, iron oxide, quantum dots, mesoporous silica) enable imaging, contrast, and drug loading. Organic systems (liposomes, polymeric nanoparticles, micelles, dendrimers) offer biocompatibility and controlled release. Hybrid nanomaterials (core–shell, Janus, and stimuli-responsive designs) combine functionalities for dual imaging and synergistic therapy

A cornerstone of nanotheranostics is the careful choice and design of nanomaterials that have the capability to undertake diagnostic and therapeutic functions in a single platform. Choices of material compositions, architectural designs, and surface properties play critical roles in shaping the PKs and biodistributions of nanocarriers, as well as compatibility with different imaging modalities and drug-loading processes [55–57]. Theranostic nanomaterials are generally divided into three main classes–inorganic, organic, and hybrid systems–each Fig. 4, with distinct advantages and challenges depending on their projected clinical use [14, 58, 59].

## Inorganic nanomaterials

Inorganic NPs possess unique optical, magnetic, and electronic properties that make them very useful for both diagnostic and therapeutic purposes. Specifically, AuNPs (AuNPs) [60] have been a focus of increasing interest in research due to the strong surface plasmon resonance (SPR) they possess, which can be utilized for photoacoustic imaging [61], photothermal therapy (PTT) [62, 63], and surface-enhanced Raman scattering (SERS) diagnostics [64]. Additionally, their versatile surface chemistry allows for precise conjugation of targeting ligands, drugs, and contrast agents [65]. Iron oxide NPs are superparamagnetic and are very effective contrast agents for magnetic resonance imaging (MRI) while also providing therapeutic benefits through magnetic hyperthermia [66]. Their intrinsic biodegradability, coupled with an attractive safety profile demonstrated in the clinic (e.g., ferumoxytol/Feraheme®) [67], further enhances their potential for medical use [68]. QDs, or semiconductor nanocrystals, exhibit fluorescence emissions that are size-tunable, with excellent brightness and photobleaching resistance, making them extremely well-suited for multiplexed optical imaging [69, 70]. However, concerns over toxicity from heavy-metal cores, e.g., cadmium [71], have limited their application in the clinic, thus prompting research into less-toxic alternatives and forms encapsulated in silica [70]. Silica NPs, in particular mesoporous silica NPs (MSNs), have a high surface area and tunable pore size, which makes them ideal for encapsulating drugs and as carriers for imaging agents [72, 73]. In addition, their surfaces can be modified to allow for stimulus-responsive release mechanisms, and their intrinsic optical transparency facilitates compatibility with imaging fluorophores [74].

Gold nanostructures, other than simple spheres, have plasmonic properties that change a lot with their shape, and this impacts how well they work for theranostics. Spherical gold nanoparticles, about 20–80 nm in size, usually have an LSPR band around 520–600 nm in dispersed media, with modest red-shifts as the diameter increases [75]. This is helpful for imaging in lab settings and for surface-enhanced spectroscopies. But, it is suboptimal for deep tissues use because it doesn't penetrate well in the visible range.

On the other hand, gold nanorods and nano-stars have a longitudinal plasmon mode that can be adjusted. Its peak can be moved into the near-infrared windows (NIR-I/II, about 650–900 and 1,000–1,350 nm) by changing the aspect ratio [76]. This makes photothermal and photoacoustic better at wavelengths that can get through tissue. Branched ‘nanostar’ shapes bring together the electromagnetic field at sharp tips, making strong hot spots. These spots greatly increase SERS signals and can make photothermal conversion work better, making them good for combined imaging and ablation.

Core–shell structures, like gold nanoshells or Au–silica core–shell particles, allow more adjustment of the LSPR by changing the core radius and shell thickness. This allows balancing NIR absorption for therapy with X-ray attenuation for CT contrast. But, these shape-dependent optical properties need to be balanced with differences in how they spread in the body, how they are opsonized, and how they are cleared. This is because particles with a high aspect ratio or highly branched particles often have different uptake patterns compared to spheres [62, 77].

So, choosing between spherical, rod-like, star-shaped, or core–shell gold platforms is a key thing to consider. It decides what combo of imaging contrast, photothermal or photoacoustic output, and pharmacokinetic behavior can be achieved in nanotheranostic applications. The figures in this paper show spheres for simplicity, but many of the strategies discussed can be applied to these other gold shapes.

## Organic nanomaterials

Organic nanocarriers exhibit biocompatibility and multifunctionality, including a broad range of functional characteristics that make them especially suitable for the systemic delivery of both hydrophilic and hydrophobic drugs [78, 79]. Liposomes, the most clinically developed class of nanocarriers (e.g., Doxil®) [80], are phospholipid bilayers that can encapsulate either water-soluble or lipid-soluble drugs [81]. These systems are carefully engineered for applications in theranostics by incorporating imaging contrast agents like Gd^3^⁺ [82] for magnetic resonance imaging (MRI) or near-infrared (NIR) dyes for fluorescence imaging [83, 84]. Polymeric NPs, including biodegradable polymers like poly(lactic-co-glycolic acid) (PLGA) [85], polyethylene glycol (PEG)-polycaprolactone (PCL) [86], and PCL [87], allow for control of drug release while providing potential for tailorable surface modifications. Their use is increasingly being adopted for dual-function systems that concurrently deliver drug payloads with contrast agents or photoresponsive items [88]. Micelles, formed by the self-assembly of amphiphilic block copolymers, act as carriers for delivering hydrophobic drugs and fluorescent labels in compact and dynamic structures [89, 90]. Dendrimers, with highly branched structures and homogenous size distributions, have multiple sites for binding and targeting delivery, drug conjugation, and imaging purposes [91, 92]. Natural systems offer an advantageous choice for modular theranostic assembly, allowing for both sequential and combinatorial strategies for imaging and therapeutic uses. Their use is often, however, limited by structural susceptibility and rapid clearance from systemic circulation [93, 94].

## Hybrid nanomaterials

Hybrid nanomaterials combine inorganic and organic components to take advantage of the synergistic properties of both classes of materials [95, 96]. One of the best examples of this approach is core–shell NPs, which have an inorganic core (e.g., gold, iron oxide, or silica) to promote imaging or thermal function and an organic shell to provide biocompatibility, a stealth effect, and drug-loading capacity [97, 98]. Stimulus-responsive hybrids [99], such as those sensitive to pH, redox gradients [100], enzyme activity, or external fields like light and magnetism, are becoming more favored for the spatiotemporal control of drug release. Such intelligent systems can remain in an inactive phase during circulation but can be selectively activated in the TME, thereby minimizing off-target effects [101]. In addition, Janus NPs [102], which have two or more discrete physical domains within one particle, enable spatial differentiation for diagnostic and therapeutic components, thus increasing functional autonomy and signal integrity in multimodal imaging techniques [77, 103].

The systematic design of hybrid theranostics, particularly those with multifunctional stratification, orthogonal targeting approaches, and AI-assisted frameworks, is an active area of research aimed at maximizing payload incorporation, circulation times, and therapeutic efficiencies [104–106]. The different material categories, taken collectively, provide a rich set of resources for developing theranostic platforms with tailored physical, chemical, and biological properties [30]. The strategic selection and combination of these materials are critical, since they determine both the system's operational functionality and its path to practical use. In the following sections, we discuss the role of AI in accelerating the design and optimization of these nanostructures, and hence the realization of their all-around diagnostic and therapeutic properties.

### Imaging modalities enabled by nanomaterials

One of the main advantages of nanotheranostic platforms is their ability to complement and integrate multiple imaging modalities, enabling accurate localization of disease, real-time evaluation of therapeutic responses, and resolution at the molecular level [107, 108]. At the core of this capability is the unique physicochemical plasticity of nanomaterials, which allows for the controlled incorporation of contrast-enhancing agents into nanoscale delivery systems [109, 110]. These platforms augment signal intensities, extend circulation half-lives, enable active targeting, and consequently enhance the specificity and sensitivity of conventional imaging systems [111, 112].

## Magnetic Resonance Imaging (MRI)

Magnetic NPs, particularly superparamagnetic iron oxide NPs (SPIONs) [113], are a leading class of nanostructures used to produce contrast enhancement in MRI [114, 115]. Their high magnetic susceptibility induces strong local field inhomogeneities, resulting in pronounced T₂ relaxation shortening (high r₂/r₁ ratio) and consequent T₂ signal attenuation. [116], thus allowing enhanced lesion detection in neoplasms, hepatic tissues, and lymph nodes [117]. By contrast, NPs loaded with gadolinium or manganese oxide [118] are designed for T₁-weighted imaging, which increases contrast brightness [119]. In addition, such systems can be functionalized with tumor-targeting ligands or with stimulus-responsive coatings to allow selective uptake and responsive imaging in targeted microenvironments, including the acidic interstitial tumor space or hypoxic tissues [120].

## Positron Emission Tomography (PET) and Computed Tomography (CT)

Nanomaterials have also been used in PET imaging through radiolabeling of NPs with isotopes such as ^64^Cu, ^89^Zr, and ^68^ Ga, which requires no chelators and hence limits interference with the physicochemical properties of the particles [121]. The high sensitivity of PET enables the quantitative tracking of NPs in deep tissues, while its combination with CT provides anatomical contextualization [122]. In CT imaging, high-atomic-number elements such as gold (Au), bismuth, and tantalum are incorporated into nanocarriers to enhance x-ray attenuation. Particular interest has been shown toward AuNPs, which offer dramatic contrast enhancement due to their strong photoelectric effects and demonstration of features of biocompatibility, inertness, and synthetic accessibility in tunable morphologies (e.g., nanospheres and nanorods) [123]. These preparations are now under investigation for dual PET/CT or CT/fluorescence imaging, thus enhancing their clinical utility [124].

## Fluorescence imaging

Nanomaterials engineered with fluorophores, QDs, carbon dots, or upconversion NPs (UCNPs) have significantly improved the performance of optical imaging [125, 126]. In 2013, Zhan et al. successfully performed HeLa cancer cell membrane imaging using NaYF_4_: 20% Yb^3+^/2% Er^3+^ targeting NPs [127]. QDs serve as an example through their provision of an extensive array of excitation wavelengths paired with narrow emission spectra to enable multiplexed fluorescence imaging with high photostability [128]. In addition, UCNPs [129] can convert low-energy NIR light into higher-energy visible emissions, thus reducing background autofluorescence and enabling deeper tissue penetration [130].

In 2018, a study by Zhang et al. introduced core/shell PbS/CdS QDs (CSQDs) that emit at ~ 1600 nm, with a CdS shell which enhances photostability and enables aqueous transfer via an amphiphilic polymer coating and PEGylation. They achieved tumor accumulation (tumor-to-normal (T/N) ratio of ~ 32) due to their brightness and large Stoke’s shift (~ 800 nm). The probes showed biliary excretion and minimal toxicity, highlighting their promise for in vivo NIR-IIb biomedical imaging [131]. In addition, covalent binding or attachment of fluorescent dyes onto NPs increases stability and photostability in biological milieus and thus enables extended imaging periods [132]. Also, surface modifications with targeting peptides or antibodies greatly enhance particle localization in tumors and thus makes fluorescence imaging an invaluable tool for intraoperative guidance as well as the prolonged evaluation of therapeutic outcomes [133, 134].

## Photoacoustic Imaging (PAI)

PAI successfully combines optical resolution with acoustic penetration, which has resulted in significant advances through the use of nanoscale materials with better NIR absorption properties [135, 136]. Gold nanorods, nanoshells, and carbon nanostructures are good photoacoustic transducers that generate acoustic signals from absorbed light [137, 138]. The synergistic combination of these NPs produces high-contrast imaging of tumors, blood vessels, and lymphatic systems, and they often outperform traditional dyes in terms of depth and resolution [139]. Stimulus-responsive NPs that change their optical absorption properties in response to environmental stimuli (e.g., pH or redox potential) enable dynamic functional imaging [140]. This ability provides insights not only into structural features but also into tumor physiology and the microenvironmental status [141].

## Raman imaging

Raman spectroscopy has the ability to provide molecular signatures, and its use in clinics has been greatly augmented by the addition of surface-enhanced Raman scattering (SERS) NPs, which dramatically enhance Raman signal intensities through localized surface plasmon resonance (SPR) [142]. Tailor-designed AuNPs and silver NPs (AgNPs), which are Raman reporter-modified and ligand-targeted, have been produced for the ultra-sensitive detection of tumors, with detection limits at the sub-femtomolar level [143]. SERS nanoprobes allow multiplexed imaging of different biomarkers and are being explored for their ability to delineate tumor margins in real-time during surgery [64, 144].

## Contrast enhancement and target specificity

The key distinguishing feature of NP-based imaging compared to conventional methods is the potential to realize simultaneous signal amplification with molecular targeting [145]. NPs prolong circulation times in the blood, penetrate tumors via the EPR effect, and may be actively targeted to specific receptors, such as epidermal growth factor receptor (EGFR), human EGFR 2 (HER2), or integrins [146]. Such targeting specificity improves signal-to-noise ratios, reduces off-target background noise, and allows precise spatial mapping of pathologies [147, 148]. Additionally, theranostic NPs are increasingly designed to enable multimodal imaging capabilities combining PET/MRI, CT/fluorescence, or MRI/PAI on a single platform [149, 150]. By integrating these imaging modalities, synergistic data acquisition is made possible: the anatomical precision of CT complements the molecular specificity of PET [151], while the real-time responsiveness provided by fluorescence is supplemented by the depth penetration of MRI or PAI [152]. Together, these developments highlight the potential of nanomaterials in transforming the functional profiles of biomedical imaging to achieve earlier detection, higher-resolution visualization, and real-time treatment monitoring, each of which is a key imperative in precision oncology [153]. In the following sections, we discuss how AI further enhances these functions by streamlining imaging interpretation, feature extraction, and the diagnostic decision-making process.

### Therapeutic applications

**Fig. 5:**
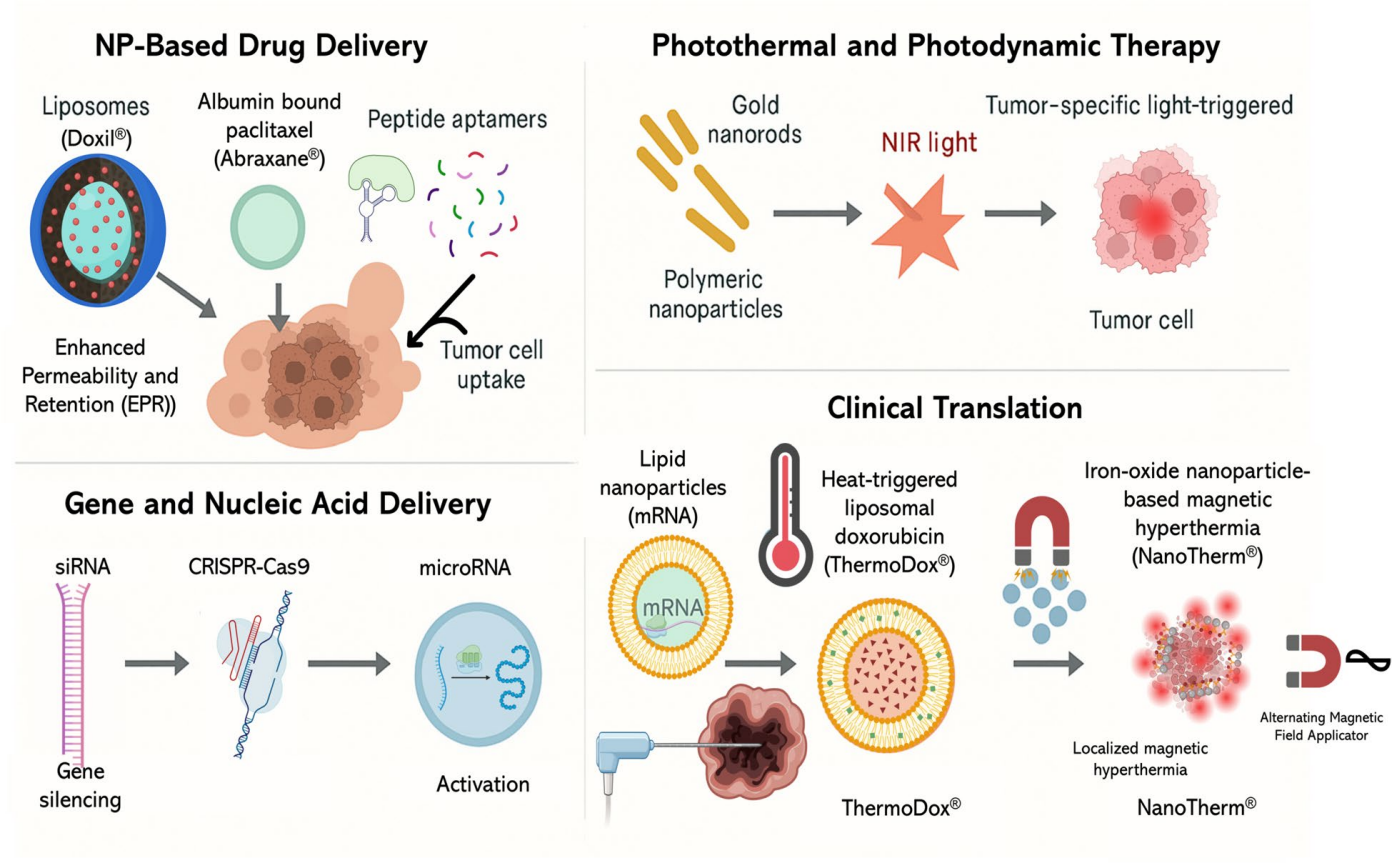
Major therapeutic applications of nanotheranostic platforms in oncology. Nanoparticle-based drug delivery systems, including liposomes (Doxil®), albumin-bound paclitaxel (Abraxane®), and peptide aptamers, enhance tumor uptake via the enhanced permeability and retention (EPR) effect. Photothermal and photodynamic therapy employs gold nanorods and polymeric nanoparticles activated by near-infrared (NIR) light for tumor-specific ablation. Gene and nucleic acid delivery strategies, such as siRNA, CRISPR-Cas9, and microRNA, enable gene silencing or activation. Clinical translation has advanced with mRNA-loaded lipid nanoparticles, heat-triggered liposomal doxorubicin (ThermoDox®), and iron-oxide nanoparticle-based magnetic hyperthermia (NanoTherm.®)

Along with enhancing diagnostic accuracy, nanotheranostic platforms have revolutionized therapeutic strategies in oncology by enabling targeted, controlled, and multimodal therapeutic regimens [154, 155]. Nanosystems are being carefully engineered to selectively accumulate in cancerous tissues, avoid premature clearance from the organism, and release therapeutic agents in a controlled fashion, both spatially and temporally [156]. Notably, several of these platforms Fig. 5, are multifunctional, enabling the combination of therapeutic efficacy with real-time imaging and response assessment, hence realizing the goal of personalized and adaptive anticancer treatments [157, 158].

## NP-Based drug-delivery systems

A major use of nanotechnology in cancer therapy is the use of nanocarriers to deliver chemotherapeutic compounds (Supplementary Table 1) [159]. By encapsulating cytotoxic drugs in nanoscale delivery vehicles, such technology improves drug solubility, protects unstable molecules, extends circulation half-lives, and reduces off-target toxicities [160, 161].

Liposomal formulations like Doxil® (PEGylated liposomal doxorubicin) are some of the first US Food and Drug Administration (FDA)-approved nanomedicines [162]. The Doxil® formulation takes advantage of the enhanced permeability and retention (EPR) principle to allow passive targeting of neoplastic tissues, and its polyethylene glycol (PEG) coating reduces immune recognition, thus prolonging systemic exposure and reducing cardiotoxicity compared to free doxorubicin [163]. Similarly, Abraxane®, an NP-albumin binding formulation of paclitaxel, improves the delivery of the drug to solid tumors via albumin receptor-mediated transcytosis. This eliminates the need for toxic solvents normally used in conventional paclitaxel formulations, thus achieving better toxicity profiles [164]. In modern use, nanocarriers leverage both passive delivery mechanisms and active targeting ligands, such as antibodies, peptides, or aptamers, that bind to tumor-specific receptors (for example, HER2, EGFR, or the folate receptor), thereby enhancing selectivity [165, 166]. Moreover, stimulus-responsive systems further optimize delivery by releasing their payloads in response to changes in pH, enzymatic activities, or redox states found within the TME [167].

## Photothermal (PTT) and Photodynamic Therapy (PDT)

These optical properties of nanomaterials have enabled the emergence of light-based therapeutic solutions, particularly PTT and PDT, thus providing targeted and minimally invasive treatment options [168]. In PTT, nanomaterials such as gold nanorods, graphene oxide, or carbon nanotubes absorb NIR light and convert it into heat, selectively ablating tumor cells Fig. 5, while sparing adjacent healthy tissues [169, 170]. These platforms can be designed for dual-functionality, carrying both photothermal agents and chemotherapeutic drugs, thereby enabling synergistic combination therapy [171]. PDT, on the other hand, utilizes photosensitizing agents usually encapsulated or conjugated in NPs that when exposed to light, generate cytotoxic reactive oxygen species (ROS) [172]. The delivery of these agents in nanocarriers improves their PK profiles, body distributions, and tumor retention, while also reducing skin photosensitivity, a common side effect of free photosensitizers [173, 174].

One advantage of these methods is their ability for external regulation, that is, light can be delivered and controlled with precision, thus allowing a degree of spatial and temporal control that cannot be achieved using systemic chemotherapy (Table 2) [178].

**Table 2:** Summary of Light-Activated Nanotherapeutics

Modality	Nanomaterial	Trigger	Action Mechanism	Clinical Status	Advantages	References
PTT	Gold nanorods	NIR laser	Localized heating	Clinical/preclinical	Minimally invasive	[175]
PDT	Porphyrin-loaded liposomes	Visible/NIR light	ROS generation	Clinical/preclinical	Dual imaging/therapy	[176]
Photoacoustic Imaging	Carbon nanomaterials	Pulsed light	Acoustic contrast	Research	High spatial resolution	[177]

*PTT* Photothermal therapy, *NIR* Near infrared, *PDT* Photodynamic therapy, *ROS* Reactive oxygen species

## Gene therapy and nucleic acid delivery

NPs have increasingly been central players in gene-targeted therapies, which face significant challenges regarding stability, delivery efficiency, and the release of endosomes after the administration of unprotected nucleic acids [179]. Polymers [180], lipids [181], and inorganic NP [182] materials have been used to deliver small interfering (si)RNA [183], messenger (m)RNA [184], microRNA [185], plasmid DNA [186], and CRISPR–Cas9 [187] elements specifically into cancer cells [188]. These delivery systems are responsible for protecting nucleic acids from degradation, facilitating cellular uptake, and allowing specific delivery into tumor tissues. One of the best examples of this trend is the development of lipid NPs (LNPs) applied for mRNA delivery, such as in the case of COVID-19 vaccines [189]. This same technological platform is now being adapted for use in cancer gene therapy, allowing for the targeted expression of mRNA for tumor suppressors, immune-modulating cytokines, or engineered T-cell receptors in the TME [190]. Additionally, co-delivery systems that encapsulate nucleic acids with chemotherapeutics or immune checkpoint inhibitors are being explored for the creation of multimodal nanotherapeutics. This is expected to enable simultaneous genetic modulation and pharmacological treatment [191, 192].

## Clinical translation and case examples

While many nanotheranostic systems are still in the preclinical testing stage, several have reached different phases of clinical development [193]. In addition to Doxil® and Abraxane®, other products, such as ThermoDox® [194], a temperature-sensitive liposomal formulation that releases doxorubicin when exposed to mild hyperthermia, have reached phase III clinical trials for treating localized unresectable recurrent and metastatic cancers, including breast cancer, bone metastasis, pancreatic cancer, and liver cancer. Similarly, NanoTherm® [195], a formulation of iron oxide NPs, was approved in Europe for use in magnetic hyperthermia therapy for glioblastomas. Apart from glioblastoma therapy, NanoTherm® has been explored for the ablation of prostate cancer in a recently completed clinical trial [194].

The above-mentioned clinical milestones highlight the great promise of nanotherapeutic platforms for a range of clinical uses. However, they also highlight the need for scalable manufacturing processes, reproducible performances, and extensive long-term evaluations of safety, which are necessary preconditions for gaining regulatory approval and clinical adoption [196, 197]. There is a burgeoning trend toward incorporating AI to drive improved design parameters, mimic in vivo results, and reduce the experimental burden of preclinical development [198, 199]. Together, these therapeutic methods illustrate the potential of nanomaterials through systematic design and tunable functionalities to overcome many limitations associated with conventional cancer therapy modalities. With further development by AI and optimization of these systems, the distinctions between static nanocarriers and dynamic therapeutic systems will increasingly fade away, making truly personalized and intelligent approaches possible in cancer therapy [200].

### Translational barriers of cancer nanomedicines

Across cancer nano-medicine platforms, late-stage failures are not random but recurrently traceable to a small set of system-level barriers (Supplementary Table 2). Many liposomal chemotherapies and conjugates (Doxil/Myocet, NC-6004, class-wide nano-taxanes) reduced acute toxicity yet failed to deliver clear OS/PFS or QoL gains over cheap generics, while EPR- and “actively” targeted systems such as MM-302, BIND-014 and CRLX101 suffered from highly heterogeneous tumor delivery and were tested in unselected, heavily pre-treated populations, diluting any benefit [201]. Drug–device combinations like ThermoDox and NanoTherm added further operational complexity, with inconsistent heat dosing and center-dependent expertise undermining pivotal trials. In parallel, several RNA and intrathecal platforms (MRX34, DCR-MYC, DepoCyt) revealed nano-specific immunotoxicity or neurotoxicity that narrowed the therapeutic window, and technically elegant constructs such as CALAA-01, exosome-based therapies (ExoASO-STAT6, iExosomes) and other sophisticated formulations ultimately proved difficult to scale reproducibly or justify economically. Taken together, the exemplars in Table 3 illustrate how modest incremental efficacy, variable delivery, suboptimal trial design, nano-specific safety liabilities, manufacturing/commercial fragility and rapid competition from IO/ADCs converge to stall ostensibly promising nanomedicines in Phase II/III [208].

**Table 3:** Comparative properties of theranostic nanomaterials

Nanomaterial Type	Size (nm)	Composition	Imaging Modality	Therapy Type	Targeting Mechanism	FDA-Approved Example	References
Gold nanoparticles (NPs)	5–100	Inorganic	CT, PAI, SERS	PTT	Passive/Active	–	[202]
Iron oxide NPs	10–200	Inorganic	MRI	Magnetic hyperthermia	Passive	NanoTherm®	[203]
Liposomes	80–150	Lipid	PET (if radiolabeled)	Chemo	Passive	Doxil®	[204, 205]
Polymeric NPs	50–200	PLGA, PEG	Fluorescence	Drug delivery	Active	–	[206]
Quantum Dots	2–10	Semiconductor	Fluorescence	PDT (if loaded)	Passive	–	[207]

*CT* Computed tomography, *FDA* Food and Drug Administration, *MRI* Magnetic resonance imaging, *NP* Nanoparticle, *NPs* Nanoparticles, *PAI* Photoacoustic imaging, *PDT* Photodynamic therapy, *PEG* Polyethylene glycol, *PET* Positron emission tomography, *PLGA* Poly(lactic-co-glycolic acid), *PTT* Photothermal therapy, *SERS* Surface-enhanced Raman scattering

These barriers define the context in which AI-enabled nanotheranostics must operate. For example, AI-assisted trial enrichment or response prediction might mitigate some issues related to heterogeneous EPR or unselected patient populations but cannot on its own overcome an intrinsically weak therapeutic index, irreproducible manufacturing, or a lack of incremental benefit over rapidly improving standards of care. Throughout Sects. " AI in NP Design and Therapeutic Modeling" and " Clinical Translation of AI- Integrated Nanotheranostics", we therefore emphasize that AI should be viewed as a tool to reduce uncertainty and improve trial design, not as a substitute for rigorous clinical evaluation or as a guarantee of successful translation.

### Advantages of theranostics

Nanotheranostics represent a remarkable improvement in oncological therapy, as they provide an integrated platform that unites diagnostic and therapeutic functionality in a single multifunctional nanostructure [209]. This (Table 3) combination offers many significant advantages over conventional, separate treatment methods, particularly with respect to spatiotemporal control, accurate dosing, and non-invasive delivery methods [210].

A major strength of nanotheranostic platforms is their ability to achieve spatiotemporal specificity. Targeted action is made possible by nanoplatforms through preferential accumulation in tumor tissues, which is obtained via passive processes such as the EPR effect or ligand-targeting approaches [211]. Furthermore, through the use of stimulus-responsive release mechanisms, drug release can be selectively triggered by intra-tumoral markers (e.g., acidic pH, enzyme activity, or redox gradients), external stimuli (e.g., light, heat, or ultrasound) [212], or surface modifications like biomimetic cell membrane coatings [213]. At the same time, the inclusion of diagnostic agents allows clinicians to assess drug distributions and tumor responses in real time, thus enabling adaptive therapeutic approaches and early detection of therapy resistance [214].

One major benefit of targeted drug delivery is that it can reduce the systemic dose of drugs, thus reducing off-target toxicity [160]. Traditional chemotherapeutic drugs, although efficacious, often have non-specific distributions within the body, which can cause undesired harm to non-cancerous tissues and side effects that restrict dosing [215]. Nanotheranostics, however, enhance therapeutic indices by delivering pharmacological action specifically within a targeted tumor environment, thus enhancing efficacy and minimizing undesirable side effects [216].

**Fig. 6:**
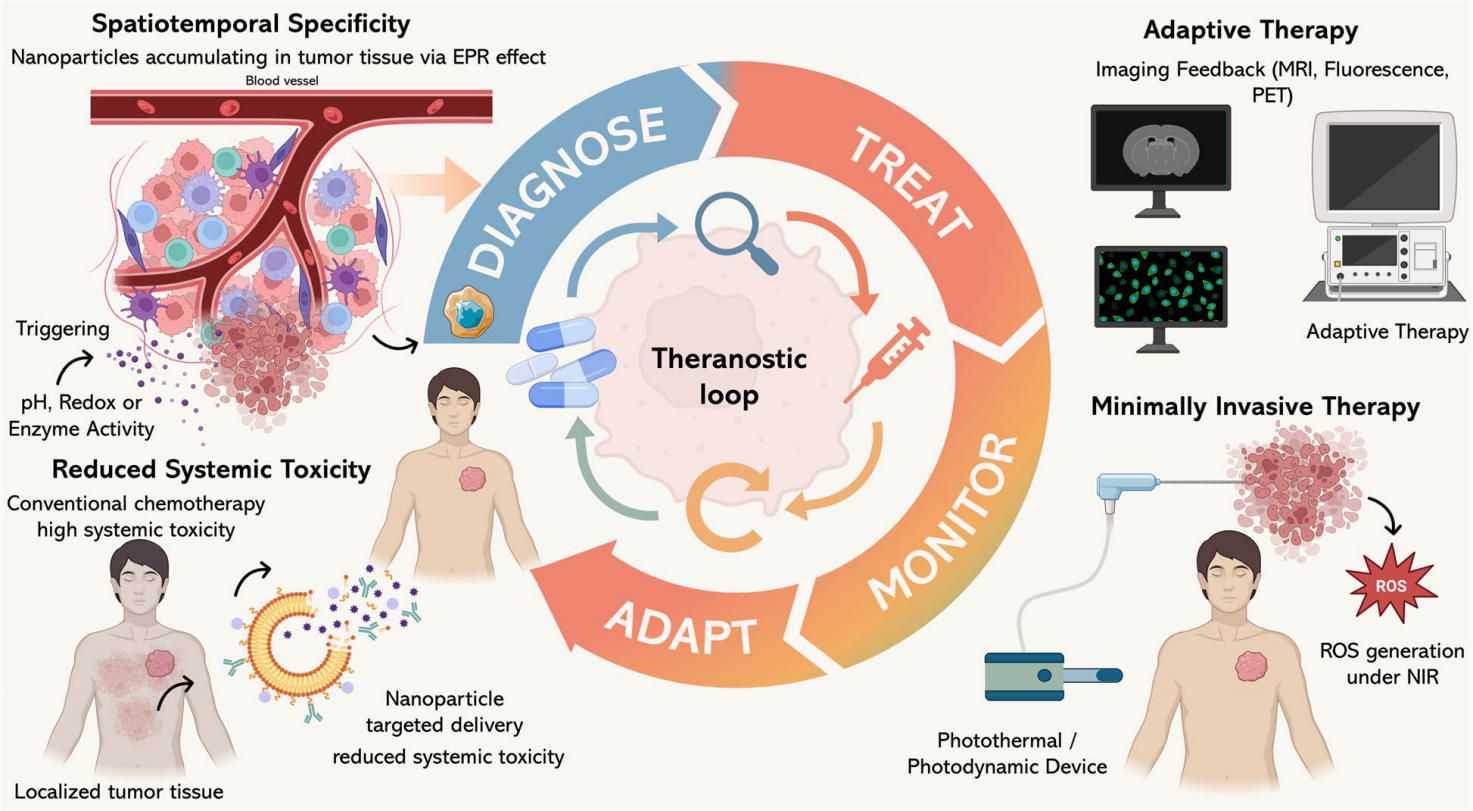
The theranostic loop for precision nanomedicine. Illustration of the theranostic cycle integrating diagnosis, treatment, monitoring, and adaptation. Nanoparticles accumulate at tumor sites via the EPR effect and can be triggered by pH, redox, or enzymatic activity for targeted therapy. Compared with conventional chemotherapy, nanoparticle delivery reduces systemic toxicity, enables minimally invasive photothermal/photodynamic interventions, and supports adaptive therapy guided by imaging feedback

In addition, the minimally invasive nature of nanotheranostic treatments differentiates them from many common clinical therapies. As an illustration, photothermal or photodynamic treatments can be externally triggered by applying non-ionizing radiation, thus avoiding the need for surgical procedures [217, 218]. Similarly, image-guided drug delivery enables targeted delivery with minimal need for repeated invasive treatments or biopsies. Such attributes Fig. 6, are especially valued for patients with compromised health or tumors located in anatomically complex areas where surgical access is a major challenge [219, 220].

Essentially, the theranostic approach supports a treatment cycle guided by feedback:

diagnose → treat → monitor → adapt.

Synergistic interaction reinforces clinical outcomes and is closely in accordance with the principles of precision medicine, which individualizes interventions based on the biological characteristics of both the tumor and patient. With AI continually improving each stage of this spectrum from the fabrication of NPs to the modeling of treatment effects, the advantages realized with nanotheranostics are expected to grow in impact and clinical relevance [221, 222].

## AI in NP design and therapeutic modeling

**Fig. 7:**
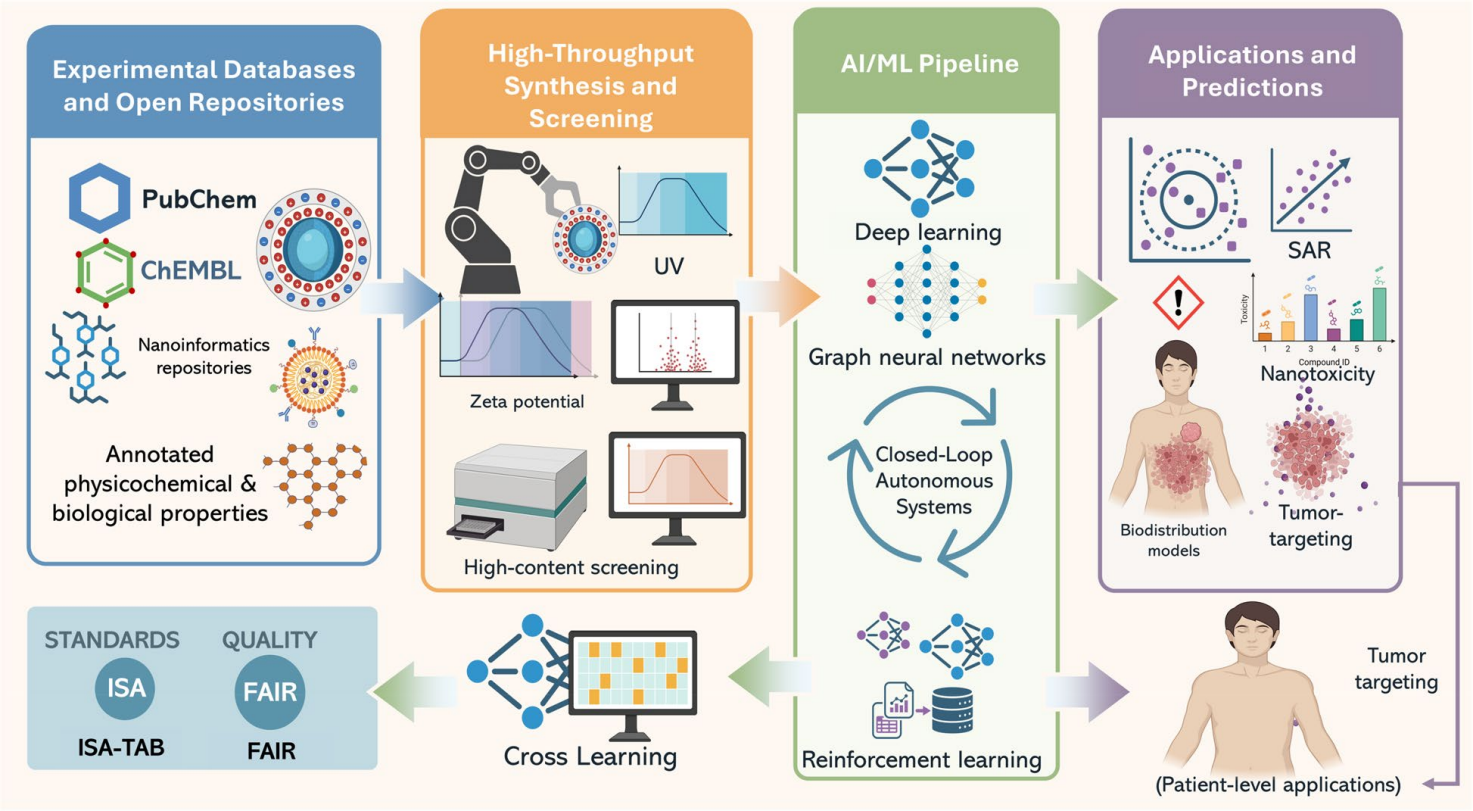
Data sources and AI pipelines for nanotheranostics. Workflow illustrating how experimental databases and nano-informatics repositories provide annotated physicochemical and biological data, which are integrated with high-throughput synthesis and screening (zeta potential, UV, high-content assays). These inputs feed into AI/ML pipelines, including deep learning, graph neural networks, reinforcement learning, and closed-loop autonomous systems. The resulting models enable key applications such as structure–activity relationship (SAR) prediction, nanotoxicity assessment, tumor targeting, and biodistribution modeling at the patient level

A major scientific challenge is posed in the design of optimized nanotheranostic platforms because the vast, nonlinear, and multidimensional design space includes a range of parameters Fig. 7, such as material composition, particle size, morphology, surface chemistry, drug encapsulation, release kinetics, targeting ligands, and biological interactions [43, 223]. Even minor changes in these variables can lead to substantial variations in biodistributions, therapeutic activities, and safety profiles. Historically, optimization of such complex systems largely relied on trial-and-error approaches, a time-consuming and expensive route that often yielded suboptimal or non-generalizable results [224].

In recent years, AI has emerged as a key tool for overcoming these challenges. On the basis of ML and DL algorithms, researchers can now identify important patterns within large, high-dimensional datasets and accurately predict NP behaviors [225]. AI not only allows for the accelerated systematic design of nanomaterials but also for inverse modeling, in which specific biological effects like tumor-targeted accumulation, an enhanced therapeutic index, or dual-modality imaging can be algorithmically used to generate optimal NP designs [226, 227].

Besides its uses in design, AI has a central function in therapeutic modeling through simulating in vivo behaviors, predicting PKs, and tailoring anticipated outcomes using digital twin models [228, 229]. Additionally, it supports the identification of toxicity hazards and promotes regulatory transparency by using explainable AI (XAI) methods, which explain the reasoning behind certain predictions [230, 231].

In this section, we discuss the TDMM framework to nanoparticle design and therapeutic modeling. The core tasks are forward design of nanotheranostic platforms (materials, size, shape, surface chemistry, ligands), prediction of key properties (size, PDI, zeta potential, corona composition, biodistribution), and inverse design of formulations that meet specified theranostic goals (e.g., NIR-tuned plasmonics, prolonged circulation, reduced RES uptake). The data include nano-informatics repositories (e.g., caNanoLab, eNanoMapper), chemical and bioactivity databases, standardized characterization and nanotoxicology assays, high-throughput and microfluidic screens, and image-level TEM/SEM and release profiles. The methods range from penalized linear and logistic models to tree ensembles, SVMs, GNNs, CNNs, VAEs, GANs, and RL, with explainability tools (e.g., SHAP, LIME) to identify influential design features, as summarized in Supplementary Table 3. The main metrics are prediction error and calibration, synthetic yield and robustness, target optical or magnetic performance, in vitro/in vivo concordance, and decision-curve net benefit for design choices, rather than direct clinical endpoints.

### Data sources for AI Models

The success of AI in the field of nanotheranostics heavily depends upon the amount, size, and type of data available to researchers for model building and validation processes Fig. 7. Unlike traditional biomedical approaches, NP-based systems require the integration of material science, chemistry, biology, and clinical metadata, thus calling for the use of diverse datasets mapping molecular properties to phenotypic traits [232].

## Experimental databases and open repositories

AI systems in the nanomedicine field increasingly draw on structured databases of chemical and biological data, many of which were initially designed for drug discovery but are now being repurposed for examining nanomaterials.

- PubChem provides over 100 million compounds with annotated physicochemical properties and bioassay results. Although not NP-specific, it supports predictive modeling of surface ligands and payload molecules used in nanocarriers [233].
- ChEMBL, is a curated bioactivity/ADMET database for small-molecule payloads and ligands, widely used for target interaction, potency (IC50/EC50), and mechanism annotations; it does not contain nanoparticle release kinetics. In NP applications, ChEMBL can indirectly inform pharmacodynamic (PD) components (e.g., Emax/EC50 in exposure–response models) and ligand/payload selection or QSAR, while NP-specific pharmacokinetics and release are modeled separately using materials/geometry-driven release models [234, 235] (e.g., Higuchi, Korsmeyer–Peppas, mechanistic polymer-degradation/diffusion) coupled to NP PBPK frameworks [236, 237]. For liposomes/polymeric carriers, PBPK/PopPK models that explicitly track encapsulated and free drug with inter-compartment release rates have been reported, enabling estimation of in vivo release and linkage to therapeutic outcome [234, 238, 239].
- Nanoinformatics databases, such as caNanoLab [240], eNanoMapper [241], and NBI Knowledgebase [242], directly catalog NP physicochemical features (e.g., size, charge, shape, and surface functionalization), biological responses (e.g., cytotoxicity and cellular uptake), and experimental protocols These datasets enable supervised learning for structure–activity relationship (SAR) modeling and facilitate cross-study comparisons in nanotoxicology [243] and biodistribution predictions [244].

In addition to these accessible public datasets, projects like the Nanotechnology Characterization Laboratory (NCL) [245] and the EU NanoSafety Cluster [246] provide standardized assay protocols (e.g., the NCL Assay Cascade) and reference materials to improve reproducibility; however, NCL-generated data are not produced under GLP. Accordingly, while these resources advance harmonization [247],, regulatory submissions requiring GLP must rely on studies conducted in appropriately certified facilities, and manuscripts should clearly report data provenance and quality controls.

## High-throughput synthesis and screening

As the design space for theranostic nanomaterials expands, high-throughput experimentation (HTE) platforms are becoming essential sources of AI-compatible data [248]. These systems combine robotic synthesis, automated characterization (e.g., dynamic light scattering, ultraviolet–visible (UV–Vis) spectroscopy, and zeta potential), and biological assays (e.g., viability, ROS generation, and immune activation) into integrated workflows [249, 250].

Beyond generic Bayesian optimization and heuristic design loops, a recent study by Canning et al*.* demonstrated an AI-integrated, fully automated synthesis platform for plasmonic nanostars that is directly relevant to nanotheranostics. Using a low-cost Arduino-controlled system driving multiple peristaltic pumps, the authors systematically varied reagent volumes and injection timings to synthesize gold and bimetallic nanostars at scale, while recording absorbance spectra, SERS enhancement, and detailed morphological descriptors. They then trained and compared several machine learning models (SVR, RF, CatBoost, XGBoost, and ANNs) to predict key optical readouts, peak wavelength, peak height, peak width, and |E|/|E₀| SERS enhancement, from the synthesis parameter space, selecting gradient-boosted tree ensembles as the most robust option under fivefold nested cross-validation. The final models were used to generate a dense look-up table over the experimental design space, enabling “on-demand” synthesis of nanostars with targeted plasmonic peaks (e.g., 808 and 1064 nm relevant for photothermal therapy) with < 1.2% deviation from the desired wavelength and optimized SERS response. This work exemplifies how AI-driven, closed-loop optimization can deliver reproducible, batch-to-batch consistent nanostar platforms for both photothermal cancer therapy and SERS-based biosensing, and provides a concrete blueprint for translating automated, ML-guided nanomaterial production into clinically oriented theranostic pipelines [251].

Microfluidic synthesis platforms are able to simultaneously generate multiple NP formulations through adjusting key parameters like polymer concentrations, mixing speeds, or solvent polarities [252, 253]. When combined with high-content screening performed against multiple cell lines or organoids, these techniques generate multimodal datasets that are typified by complex physicochemical and biological relationships [254, 255]. These platforms provide carefully curated datasets for training predictive models (e.g., prediction of cytotoxicity based on size and charge parameters) while also enabling real-time feedback mechanisms in autonomous laboratories [256]. In these environments, AI algorithms iteratively direct new experiments based on prior results. These closed-loop systems are the key to advancing AI-powered inverse design approaches in the field of nanomedicine [257, 258].

## Challenges in data curation and standardization

Despite recent advances, most nano–bio interaction datasets remain relatively small and heterogeneous, and are far from the large, harmonized corpora often assumed in generic AI discussions. Protein corona composition, targeting ligand performance, cellular uptake, and toxicity can vary markedly with experimental context-including cell line model, animal strain, serum or protein source, media composition, assay protocol, and instrumentation, which introduces substantial domain shift between laboratories and studies [259, 260]. Under these conditions, models that perform well on a single-laboratory dataset cannot be assumed to generalize reliably to other settings or patient populations without careful external validation. Initiatives such as ISA-TAB-Nano [261] the eNanoMapper ontologies, and the FAIR (findable, accessible, interoperable, reusable) principles [262] attempt to standardize descriptors, units, and metadata reporting, and programs like the NCL and EU NanoSafety Cluster promote reference materials and assay cascades. However, these efforts are only a partial solution; robust AI generalization will require harmonized nano-bio descriptors, explicit documentation of experimental context, and prospective multi-center datasets designed with model transportability in mind [263]. As the field moves towards automated, closed-loop development workflows, the need for quality, interoperable, and biologically annotated NP datasets is likely to grow, to enable translation of findings to the clinic [264].

### AI Techniques used in designing nps and predicting functions

**Fig. 8:**
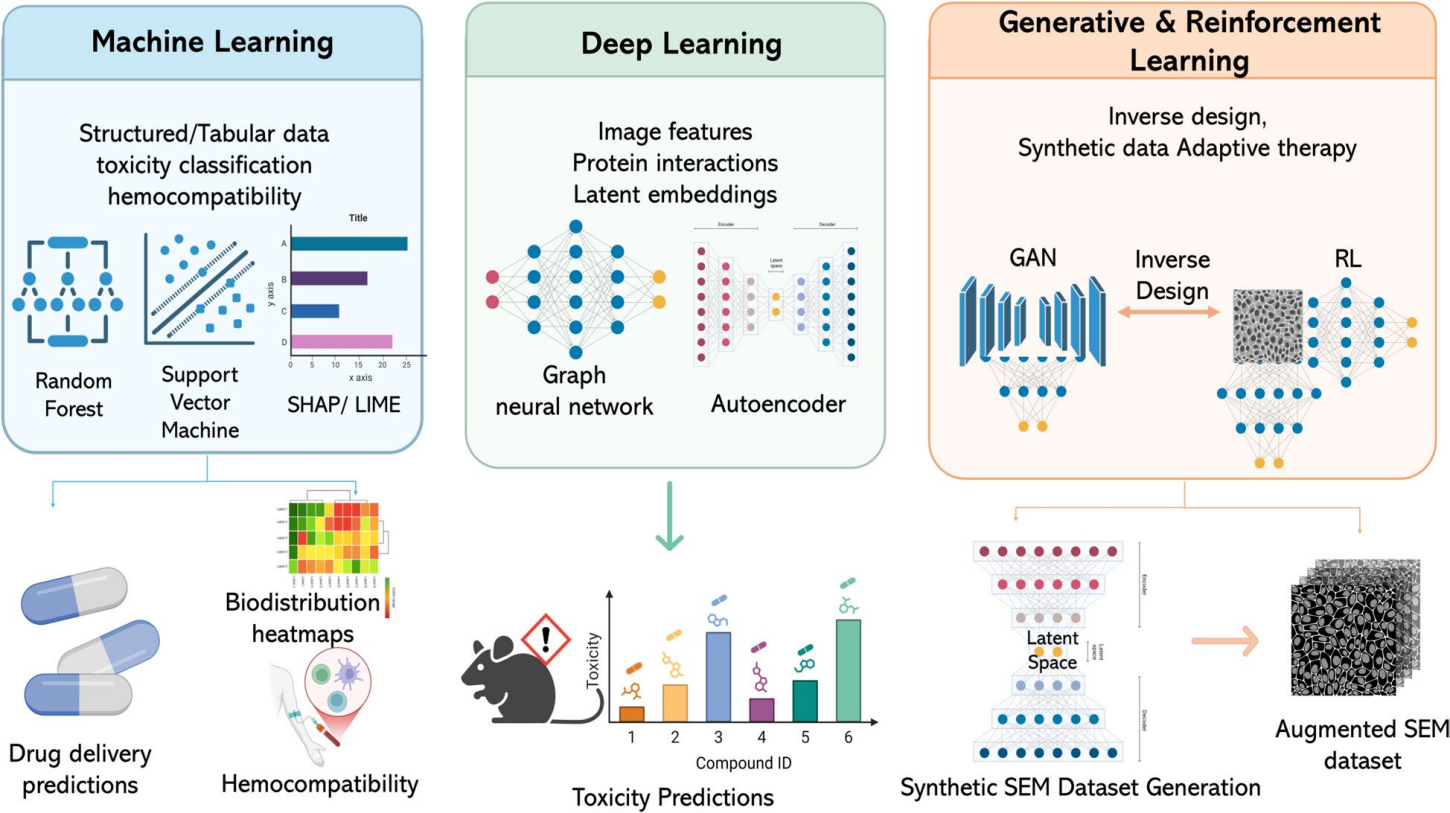
AI techniques for nanoparticle design and prediction. Machine learning models (random forest, SVM, SHAP/LIME) enable drug delivery predictions, biodistribution mapping, and hemocompatibility assessment from structured data. Deep learning approaches (graph neural networks, autoencoders) capture image features and protein interactions for toxicity predictions. Generative and reinforcement learning frameworks (GANs, RL) support inverse design, adaptive therapy, and creation of synthetic or augmented SEM datasets

Applying AI in the area of nanomedicine depends on the ability of different AI approaches to integrate knowledge from high-dimensional, complex datasets and clarify subtle intercorrelations among NP designs, physicochemical properties, and biological activities [21]. A wide variety of ML, DL, and generative modeling approaches Fig. 8, have been successfully applied across nanotheranostic design-to-development pipelines, each of which brings specific strengths to prediction, optimization, and exploration of the design space [265, 266].

## Parsimonious baselines and model escalation:

In keeping with the principle of parsimony, analyses of structured clinical and physicochemical datasets should begin with interpretable linear baselines and escalate complexity only when performance or decision utility improves by a clinically meaningful margin [267–269]. For classification, an appropriate baseline is logistic regression (with ℓ2/ℓ1/Elastic-Net penalties when $p\approx n$ or $p>n$) [270]. For regression, an appropriate baseline is linear/Elastic-Net regression [271]. Such baselines offer (a) stable effect estimates with shrinkage to mitigate overfitting; (b) transparent coefficients interpretable as standardized β or odds ratios (95% CI); and (c) well-calibrated probabilities after Platt scaling or isotonic regression, when required [272].

## Baseline-first protocol checklist

1. Pre-processing: define a missing-data strategy (single imputation with indicator, or MICE where appropriate) [273]; standardize continuous features; assess multicollinearity (pairwise r, VIF) [274].
2. Model: fit logistic/linear models with Elastic-Net; tune α via inner cross-validation and λ along the regularization path [275].
3. Validation: use nested cross-validation or repeated K-fold with an untouched outer test fold to avoid optimistic bias [276, 277].
4. Metrics: report AUROC/AUPRC (classification) or RMSE/MAE (regression) alongside calibration (Brier score, ECE, reliability plots) [278].
5. Decision relevance: quantify net benefit (decision-curve analysis) and/or NRI when operating thresholds have clinical consequences [279].
6. Escalation rule: consider RF/XGB/SVM/NN only if improvement exceeds a pre-specified margin (e.g., ΔAUROC ≥ 0.03 or a material gain in calibration/net benefit), and retain linear baselines as comparators in reporting [280, 281].

When mild non-linearities are anticipated but full black-box models are not warranted, generalized additive models (GAMs) [282] provide additive smooth terms with partial-dependence-style interpretability, preserving transparency while capturing key curvatures [283]. Because datasets in nanotheranostics frequently have limited sample sizes, correlated descriptors, and inter-lab domain shift, penalized linear baselines reduce variance, surface robust signals, and yield communicable explanations for translational stakeholders (clinicians, regulators). Escalation to more complex models is appropriate when they deliver calibration-aware and clinically significant gains rather than nominal improvements in discrimination alone [284].

## ML approaches: interpretability and management of structured data

Traditional ML methods (Supplementary Table 3) are still widely used in nanomedicine, mostly due to interpretability, robustness, and effectiveness when handling smaller, structured datasets.

- Random Forests (RFs) are particularly beneficial because of their ensemble approach, which allows recognition of nonlinear interactions among NP attributes (e.g., size, zeta potential, and surface functionalization) and resulting effects, such as cytotoxicity or cellular uptake. RF models were found to exhibit a high level of accuracy in predicting hemocompatibility, protein corona formation, and tumor accumulation patterns [285, 286].
- Support Vector Machines (SVMs), especially when coupled with kernel functions, display significant efficacy in high-dimensional applications and have been utilized to classify nanomaterials on the basis of their toxicity, encapsulation capability, or therapeutic efficacy in targeted delivery. Their ability to handle imbalanced datasets is particularly beneficial in the scope of nanotoxicology, where toxic formulations are rare but essential for clinical purposes [287, 288].

The models used in ML are often the first step in predictive pipelines and are most often coupled with feature selection methods or tools for increased interpretability, like Shapley additive explanation (SHAP) and local interpretable model-agnostic explanation (LIME), to explain important design parameters [289].

## Deep Learning (DL): acquisition of representations from multimodal and unstructured data

Given the increasing availability of image, structure, and omics data in nanomedicine, DL methods offer a unique advantage through the autonomous extraction of hierarchical features from raw inputs.

- Convolutional Neural Networks (CNNs) are frequently applied to analyze transmission electron microscopic (TEM)/scanning electron microscopic (SEM) images of NPs [289], enabling classification based on shape, aggregation, and surface morphology. CNNs have also been used for predicting drug release curves by learning from kinetic plots and encapsulated drug profiles [290, 291].
- Graph Neural Networks (GNNs) have shown impressive potential in representing NPs in terms of molecular or structural graphs, where nodes represent atoms, functional groups, or surface domains, and edges are defined by chemical bonds or spatial proximity. GNNs have achieved top performance in predicting interactions between NPs and proteins, as well as cell targeting [292, 293].
- Autoencoders and Variational Autoencoders (VAEs) have been applied to a number of unsupervised learning problems, such as feature compression, clustering of formulation libraries, and anomaly detection in batch synthesis processes. Often, the representations from their latent space are used as inputs to downstream predictive or generative models [294, 295].

## Generative models and reinforcement learning: structuring the unknown

Besides its predictive applications, AI is increasingly utilized in generative design to enable innovative design of NPs for specific therapeutic or diagnostic goals.

- Generative Adversarial Networks (GANs) were utilized by Bals & Epple in 2023 to generate artificial scanning electron micrographs of NPs with well-defined morphological characteristics. The method used includes converting particle assemblies created using Blender software into synthetic SEM images by applying a GAN architecture. The inclusion of height maps, which provide a three-dimensional (3D) description of surface topography, significantly increases the realism of images by adding depth information to the generation process. Synthetic images created in this process are then used to enrich training datasets for image-based classification purposes as well as to create novel morphological variations that do not exist in empirical data. As a result, convolutional neural networks (CNNs) trained on these artificial SEM images are able to carry out tasks such as NP identification and classification. Although CNNs trained on synthetic data were found to have marginally lower performances compared to those trained on original SEM images, this procedure suggests a potential method for generating labeled training data suitable for segmentation and classification in SEM image analyses using DL methods [296].
- VAEs and Conditional VAEs (CVAEs) can serve in conditional generation pipelines, producing NP architectures conditioned on specific properties (e.g., high tumor retention, low liver accumulation), thus aiding inverse design [297–299].
- Reinforcement Learning (RL) frameworks enable the exploration of design parameters by deploying AI agents in a trial-and-error fashion [300]. In nanomedicine, RL has been used to optimize multistep synthesis processes, predict optimal formulation methodologies that conform to biological constraints, and control adaptive treatment methodologies for digital twins [301–303].

Simultaneously, these AI techniques provide a solid computational basis for the systematic design, prediction, and optimization of nanotheranostic systems. Good model selection depends on the nature of the data, the task complexity, and the need for interpretability; however, their combined use is rapidly leading to a new era of intelligent nanomedicine translating discovery processes from empirical estimations to one driven by learning algorithms capable in navigating vast design spaces.

When considering the move to clinical settings, not all AI families listed in Supplementary Table 3 are equally ready. Models that will guide real patient care have to meet standards beyond just high accuracy. These standards include: (i) training on clinically important results (like response to treatment, survival rates, or toxicity) using adequate sample sizes; (ii) strong validation within the study and across different medical centers, using planned analyses; (iii) proof of proper calibration and clear clinical advantage (assessed by decision-curve analysis) at medically relevant levels; (iv) providing understandable interpretations or measures of uncertainty at the individual case level (using methods like SHAP or Bayesian approaches) to aid shared decision-making; and (v) feasibility within regulatory and practical limits, including pipelines that can be repeated, observation for changes over time, and compliance with requirements for high-risk Software as a Medical Device. In effect, simpler, well-calibrated models (such as penalized regression, tree ensembles with SHAP, or Bayesian models) are more easily applied in clinical practice right now. More complicated designs (like diffusion models, NeRFs, or HyperNetworks) are mainly research tools used to support design, enhancement, or representation learning before clinical decisions are made [304].

### AI in property predictions and optimization

Nanomedicine platform effectiveness and safety features are profoundly influenced by key physicochemical properties, such as NP size, morphology, surface charge, and chemical makeup. Such properties control biological interactions that cover cellular internalization, blood circulation lifetimes, immune system evasion, and tumor penetration [305–309]. Due to the intricate and non-linear correlations between design parameters and biological outcomes, AI has emerged as an important tool for accurately predicting and optimizing NP functions, in preclinical and clinical development [310].

In biological settings, nanoparticle's coating, such as PEG, zwitterions, polysaccharides, or targeting ligands, mainly determine its biological identity. The coating influences how a protein corona forms, how opsonization occurs, and how the reticuloendothelial system (RES) recognizes it [311, 312]. The ensuing protein corona-not the bare surface-governs cell association, biodistribution, and immune interactions. PEGylation and related hydrophilic coatings generally reduce opsonization and prolong circulation (with dependence on PEG MW and surface density), but effects are context-specific and may be offset by anti-PEG responses or “accelerated blood clearance [313, 314].

## Physicochemical property predictions

AI models have demonstrated high efficacy in predicting NP structural features from formulation parameters and vice versa.

For example, the size and polydispersity index (PDI), which are important for understanding colloidal stability and cellular internalization [315], can be accurately predicted using methods like RFs, gradient boosting machines (GBMs), or deep neural networks (DNNs). These models are based on datasets that capture formulation parameters like solvent ratios, stirring speeds, and polymer concentrations [316]. The shape- and aspect-ratio-related parameters influencing biodistributions and endocytic processes [316] can be predicted through the use of convolutional neural networks (CNNs) applied to SEM/TEM images [317], or otherwise through the use of graph-based models that capture morphological properties [318]. Surface charge and zeta potential modeling, which are critical factors for protein corona formation and immune clearance, can be performed using support vector machines (SVMs) and ensemble approaches. The modeling often involves inputs such as pH, ionic strength, and ligand surface density [319, 320]. Such predictive models are increasingly incorporated into automated formulation platforms, enabling real-time feedback and adaptive control during NP synthesis, a key step towards autonomous discovery of nanomaterials.

## AI for biodistribution and targeting efficiency

An important use of AI in the field of nanomedicine is its ability to predict in vivo biodistributions and targeting efficiencies, a task that has been difficult to achieve using traditional PK models, due to the complexities involved in the enhanced permeability and retention (EPR) phenomenon along with the heterogeneity of biological barriers [321, 322].

ML algorithms based on imaging data (e.g., MRI, PET, and NIR fluorescence (NIRF)) and biologic markers (e.g., tumor vascularity and organ-specific fenestrations) have shown great efficacy in predicting NP accumulation in target and non-target tissues [323, 324]. AI-based models for predicting ECM permeability were generated [325] using attributes of the TME, such as interstitial fluid pressure, collagen density, and angiogenic markers, to guide the design of NPs with improved extravasation and retention profiles [326, 327]. To increase the efficiency of targeting ligands, approaches that integrate AI combine receptor density data, NP valency, ligand-receptor binding affinity, and steric hindrance to mimic binding kinetics and cellular internalization [328, 329]. Recent efforts have utilized reinforcement learning algorithms to optimize surface functionalization strategies in order to improve delivery specificity to neoplasms and lowering off-target toxicity [330]. In addition, PK modeling has evolved using hybrid AI-mechanistic models, where neural networks are trained using in vitro-in vivo correlations (IVIVCs) and physiologically based PK (PBPK) simulations to deduce system-level dynamics from lab data [331–333].

## Clinical and regulatory implications

The ability of AI to accurately predict biodistributions or deposition of substances in certain organs [334, 335] and the triggering of therapeutic targets is an important advantage in reducing the need for preclinical animal testing, streamlining dosing regimens, and tailoring treatment regimens [336]. The US FDA and European Medicines Agency (EMA) have begun exploring AI-augmented models for assessing risk–benefit ratios and toxicity predictions, thus highlighting the translational value of predictive modeling in nanomedicine [337, 338]. In summary, AI-based tools for property predictions and optimization are not only accelerating the process from design to function but also enabling a more systematic, data-driven approach to personalized nanotherapeutics–a strategy with the potential for greater precision, safety, and clinical relevance in cancer treatment [339].

### AI-guided inverse design of theranostic NPs

**Fig. 9:**
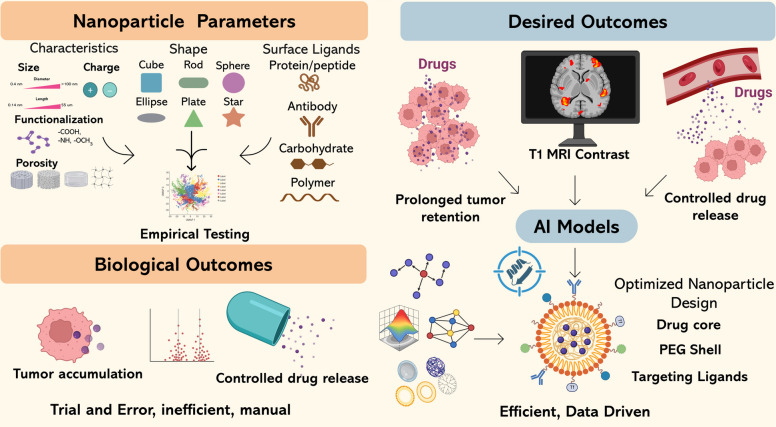
AI-guided inverse design of theranostic nanoparticles. Traditional trial-and-error testing of nanoparticle parameters (size, shape, ligands) yields limited biological outcomes. AI models enable data-driven optimization of nanoparticle design, improving tumor retention, MRI contrast, and controlled drug release through rational integration of core materials, PEG shells, and targeting ligands

The classical method of nanomedicine development generally follows the forward design paradigm, whereby parameters of NPs, i.e., size, composition, and ligand density, are iteratively tuned, followed by empirical evaluation of biological responses [340, 341]. This approach, while yielding many clinically accepted nanocarriers Fig. 9, is plagued by inefficiency in terms of time, poor use of available information, and scalability issues, especially with multifunctional theranostic systems [321, 342]. To overcome these shortcomings, AI can enable the transition towards an inverse design paradigm, where the therapeutic or diagnostic objective is first specified, and the system independently designs optimal NP strategies to achieve that objective [343, 344].

## Principles of inverse design

In the context of inverse design, the problem is stated as follows:

“Given a target function (e.g., drug release kinetics, imaging contrast at a specific wavelength, or tumor accumulation efficiency), what nanoparticle configuration is most likely to achieve this?”

Unlike forward modeling, which defines mapping from design parameters to resulting outcomes, inverse models perform a reverse-mapping operation. Often using generative or probabilistic approaches, inverse models sample viable NP candidates from a learned design space. The approach enables accelerated exploration of the formulation-function landscape, guiding experimental synthesis to high-promise candidates and reducing the trial-and-error process [345–347].

## AI Strategies in inverse design

A wide variety of AI methods can be utilized for the reverse engineering of theranostic NPs.

Variational Autoencoders (VAEs) and Generative Adversarial Networks (GANs) have been utilized to obtain latent representations of NP structures and design innovative candidates with specified physicochemical and biological properties. For instance, VAEs can be used to design lipid NP formulations that are optimized for specific drug-release properties [348], while GANs can enable incorporation of new surface ligand designs for improved targeting efficiency [349].

Reinforcement learning (RL) frameworks allow AI agents to iteratively tune parameters in nanomaterial design, including the core material and PEGylation density, with the goal of maximizing a reward function [350] defined by therapeutic efficacy, biodistribution, or safety metrics. In a study by Pereira T. et al., a recurrent neural network generator was trained with RL to bias molecule generation toward selective blood–brain barrier (BBB)-permeable compounds, illustrating how RL can tune design constraints like core composition and PEGylation density [351]. Graph-based RL, such as graph convolutional policy networks (GCPNs), further enable stepwise generation of graph-structured molecules to satisfy multi-objective rewards, implicitly modeling NP structures as molecular graphs [352].

Although explicit studies applying GNN-guided generative models to inorganic–organic theranostics with predicted metrics like photoacoustic signal strength or BBB penetration are still emerging, the methodology is well established. Molecules and hybrid nanostructures can be modeled as graphs, allowing GNNs to learn structure–function mappings and enable exploration of novel designs optimized for defined performance metrics [339].

## Few-shot learning in limited data scenarios

A major challenge in using AI for the inverse design of NPs is the unavailability of high-quality, standardized data, particularly relating to novel or multifunctional NP systems. In response to this limitation, several strategies including few-shot learning and transfer learning have been devised [353].

Few-shot performance improves materially when models are trained on integrated, multi-source datasets that pool assays, formulations, and experimental conditions, provided that sources are harmonized and batch effects are controlled [354]. In nanomedicine, this “information fusion” combines core/coating descriptors with contextual metadata (cell line or tissue, media/protein source, dose/time, readout type) across studies, increasing task diversity and enabling models to learn transferable structure rather than overfitting a single lab’s regime [355]. Practical pipelines: (1) schema/ontology alignment and unit normalization (e.g., ISO/OECD descriptors; eNanoMapper ontologies) [247]; (2) quality filters and assay mapping (endpoint equivalence classes); (3) batch-effect mitigation (e.g., ComBat-style adjustments for continuous endpoints, domain-adversarial training or importance reweighting for classification) [356]; and (4) multi-task/meta-learning that leverages shared representations with uncertainty estimation for out-of-domain queries. Repositories such as eNanoMapper and the NanoCommons KnowledgeBase already aggregate physicochemical, hazard, exposure and fate data under FAIR principles, making them natural substrates for fusion-based modeling [357–359].

Few-shot learning allows AI programs to learn from a few labeled examples through leveraging existing knowledge from related domains or tasks. A recent GAN-like latent framework by Liu et al. [360] tailored for synthesizable ionizable lipids enabled the proposal of new lipid candidates with favorable delivery properties [353, 360].

A recent case study by He et al. (2024) [361] introduced NANO.PTML for read-across prediction in neurosciences, explicitly fusing information from different nanosystems and assay contexts to extrapolate to new, data-poor compounds. Their workflow aggregates biological activity parameters, cell-line context, NP shape, measurement conditions, and coating agents into a unified learning problem, demonstrating how multi-source integration can compensate for thin per-compound data while preserving mechanistic signal for prediction. This illustrates a general recipe: curate cross-system descriptors + context, harmonize endpoints, and train models that borrow statistical strength across related nanosystems to enable few-shot or even zero-shot predictions for novel candidates.

Meta-learning (learning-to-learn) techniques train models that can rapidly adapt to new NP tasks with minimal fine-tuning, making them ideal for emerging applications where experimental data are scarce or costly to obtain. Recent work such as GS‑Meta demonstrated a strong performance on molecular property tasks under few-shot constraints [362], underscoring the potential for similar adaptations in NP design for theranostic endpoints These methods can have high effectiveness in the field of theranostics, where functional endpoints (e.g., the effectiveness of image-guided drug delivery) can be difficult to obtain at scale; however, they can be inferred from similar systems by imposing meta-learned priors [363, 364].

Pure meta-learning helps when per-task labels are scarce but tasks are numerous and comparable; information fusion is preferable when labels are sparse overall, yet heterogeneous sources exist that can be reconciled through standardized descriptors and context variables. In practice, the strongest performance often comes from hybrid strategies: fused multi-source pretraining (multi-task or self-supervised) followed by task-aware adaptation (few-shot fine-tuning) on the local distribution, with external validation across labs/species to quantify robustness [365–368].

Even with these improvements, many present AI systems provide layered, not truly personalized, care. Models usually learn from group data and find links that show the 'typical' patient in the training data. But they may miss how germline and somatic genetic changes, tumor-environment interactions, organ problems, multiple medicines, and complex diseases impact drug behavior and treatment response. Specifically, predictions often worsen when a new patient's tumor blood supply, macrophage activity, structural makeup, or past treatments vary much from the training data. Most datasets only encode the tumor environment through some static images or tissue samples, instead of detailed, ongoing measurements. So, current models don't yet record changing blood flow, vessel permeability, or immune structure during treatment. As a result, AI-run nanotheranostic models should now be seen as ways to sort risks and improve designs for groups or subgroups, not as trustworthy predictors of individual patient results or exact drug doses [369, 370].

Whereas Sect. " AI in NP Design and Therapeutic Modeling" primarily addresses design-level AI that proposes or optimizes nanoparticle formulations, the following section concentrates on AI systems whose outputs are intended to inform oncologists’ decisions about who should receive nanotherapies, how they should be combined and sequenced, and when they should be continued or stopped.

## Clinical translation of AI-Integrated nanotheranostics

**Fig. 10:**
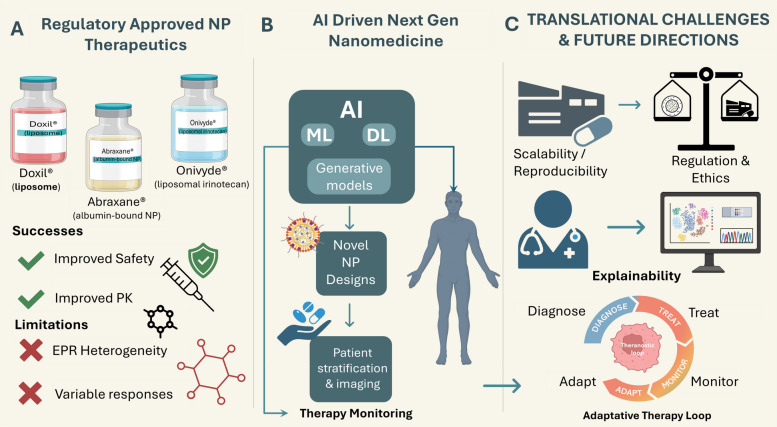
Clinical translation of AI-integrated nanotheranostics. **A** Regulatory-approved nanomedicines such as Doxil®, Abraxane®, and Onivyde® have demonstrated improved safety and pharmacokinetics but remain limited by EPR heterogeneity and variable responses. **B** AI-driven next-generation nanomedicine leverages machine learning, deep learning, and generative models to design novel nanoparticles, enable patient stratification, and guide therapy monitoring. **C** Translational challenges and future directions include scalability, reproducibility, regulatory frameworks, ethical considerations, and the development of adaptive, explainable therapy loops

In clinical practice, oncologists mainly require AI support for three therapy-guiding questions rather than for nanoparticle design per se: (i) patient selection, which patients are likely to derive meaningful benefit from nanoparticle-based interventions compared with standard systemic options; (ii) therapy adaptation, when to escalate, de-escalate, combine, or discontinue nano-enabled regimens based on early evidence of benefit or harm; and (iii) response confirmation, how to integrate imaging and biomarker readouts to distinguish true response from pseudo-progression or pharmacologically futile exposure. Sect. " Clinical Translation of AI-Integrated Nanotheranostics" therefore applies the TDMM framework to these decision-centric tasks: using clinical and imaging data (tumor burden, perfusion, vascularity, immune contexture, ctDNA) to train methods such as survival models, imaging-based deep learning, and digital twin–like simulators, and evaluating them with metrics that matter at the bedside (calibration, decision-curve net benefit, subgroup performance) rather than design-only endpoints. In this framing, AI is positioned as decision support layered on top of existing nanomedicines, rather than as a purely upstream tool for material optimization [18]. Despite considerable advancements that have been made in the area of laboratory-based AI-driven theranostic nanotechnologies, their clinical utility in real-world clinics depends upon successful clinical translation [371, 372]. In an effort to close the gap between research findings and therapeutics that are ready for patient implementation, verification of efficacy and safety and integration of AI systems into regulatory frameworks are required. The convergence of nanotechnology and AI is fostering unprecedented levels of personalized treatment, flexibility, and real-time decision-making support; however, these innovations elicit new challenges that complicate traditional clinical development paradigms [373]. Several nanotherapeutics driven by AI are making the leap from preclinical to clinical development, Fig. 10, such as imaging-guided drug delivery, cancer target phototherapy, and signal-driven intelligent release systems [374, 375]. At the heart of these developments is the ability of AI to classify patients based on molecular and phenotypic information, simulate PK populations across diverse patient groups, and mimic expected therapeutic effects through new digital twin models [376, 377]. However, integration into healthcare environments calls for more than technological readiness alone. Clinical efficacy depends on several factors, such as interpretability, reproducibility, and safety validation across different populations, as well as adherence to ethical guidelines and regulatory requirements for both nanomedicine and AI elements [378, 379]. This section takes a closer look at the realm of clinical translation and provides case studies of NP formulations involving AI-facilitated patient stratification and therapy monitoring, complemented by careful examination of the infrastructural, regulatory, and ethical factors that drive the journey towards concrete real-world applications [380].

### Regulatory-approved and pipeline products

Successful clinical translation of NP-based therapeutics represents a landmark development in the history of nanomedicine. Doxil®, Abraxane®, and Onivyde® are all commercial products that represent major milestones, demonstrating that nanocarriers can successfully address long-standing limitations of conventional chemotherapy. However, while these approved systems have had significant effects, they also reflect both the promise and the limits of first-generation nanomedicine, especially compared to the emerging generation of AI-assisted nanotheranostics [381].

## FDA-approved NP therapeutics: successes and limitations

Doxil® (PEGylated liposomal doxorubicin) is the first US FDA-approved nanotherapeutic that improves PK properties and reduces cardiotoxicity for treating ovarian cancer and Kaposi's sarcoma. Nevertheless, even with a longer circulation half-life and improved safety profile, the efficacy of accumulation in tumors has not been maximized, mainly due to heterogeneity in EPR effects [382].

Abraxane®, a paclitaxel albumin NP formulation, improved the drug solubility and eliminated the need for toxic solvents. Regulatory approval was granted for its application in treating breast, lung, and pancreatic malignancies. Targeting specificity was still largely passive, and treatment outcomes revealed marked variations among patients, stressing the need for personalized therapeutic approaches [383].

Onivyde®, a liposomal form of irinotecan, exhibited benefits in treating pancreatic ductal adenocarcinoma (PDAC) through enhanced drug deposition in neoplastic tissues. However, real-world clinical use revealed challenges in the ability to predict responses and alter dosing regimens, thus emphasizing the limitations of standardized, rigid nanocarrier systems [384].

These examples demonstrate that while NP-mediated delivery can minimize toxicity and increase circulation times, it does not necessarily ensure precise targeting of tumors, nor does it adapt in real-time to patient-specific conditions, challenges that AI is well-suited to overcome.

## AI-Influenced NP systems in clinical trials

The next generation of nanotherapeutics is increasingly adopting ML-, DL-, and AI-guided design approaches to guide formulation development, patient stratification, and treatment evaluations. Although few AI-amplified NP platforms have had widespread regulatory approval thus far, a growing number are beginning early human clinical trials, supported by compelling arguments for the likely success of translation approaches [385, 386].

From a clinical oncology standpoint, the realistic near-term role of AI-guided nanotheranostics is concentrated in a few clearly defined scenarios rather than across all solid tumors. The most plausible beneficiaries are patients with highly vascularized, fast-growing malignancies in which nano-delivery can meaningfully augment drug exposure (e.g., selected hepatocellular, renal, and triple-negative breast cancers), and those with peritoneal carcinomatosis or compartmentalized disease amenable to intraperitoneal or other locoregional administration [387]. In these settings, AI models can be trained on routinely acquired imaging (multiphase CT, DCE-MRI, PET/CT) and clinical covariates to (i) identify patients with favorable perfusion, permeability, or peritoneal distribution patterns for nanoparticle accumulation, (ii) quantify early on-treatment changes in nano-tracer uptake or residual disease to guide escalation, de-escalation, or early switching, and (iii) integrate toxicity, comorbidity, and prior-therapy history into individualized risk–benefit estimates. At the same time, we underscore that complex nano-constructs entail substantial manufacturing, quality-control, and cost-of-goods burdens; therefore, AI-enabled nanotheranostics are likely to be justifiable only in indications where these operational constraints can be balanced against clear gains in survival, organ preservation, or quality of life, consistent with the translational barriers discussed in Sect. " Translational Barriers of Cancer Nanomedicines".

## Lessons learned and translational considerations

Several critical lessons have emerged from the trajectory of both traditional and AI-influenced nanotherapeutics.

i. Passive targeting is insufficient in most clinical settings due to tumor heterogeneity and the expression of unpredictable EPR effects [388].
ii. Scalability and batch-to-batch reproducibility remain technical bottlenecks, especially for AI-designed multifunctional nanostructures [196, 389].
iii. Clinical trial design must evolve to accommodate AI models as co-diagnostic tools, requiring new regulatory frameworks for SaMD integrations [390, 391].
iv. Explainability and trustworthiness of AI systems are essential for clinician adoption and regulatory endorsement [392, 393].

In essence, the integration of AI promises to transform NPs from static drug delivery tools into dynamic, adaptive therapeutic agents capable of responding to patient-specific cues. However, regulatory pathways, trial designs, and manufacturing infrastructure must also co-evolve to realize this potential [394].

**Fig. 11:**
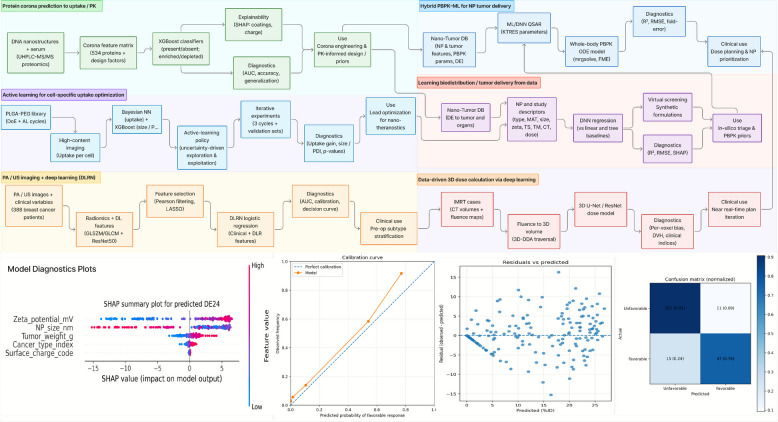
Schematic overview of six AI workflows for nanotheranostics: (1) protein corona prediction to uptake/PK, (2) active learning for cell-specific uptake, (3) PA/US imaging with deep learning (DLRN), (4) hybrid PBPK–ML tumor delivery modeling, (5) data-driven biodistribution/tumor delivery prediction, and (6) deep-learning based 3D dose calculation. Bottom panels showing feature contributions to predicted 24-h delivery efficiency (DE24), (SHAP summary plot, calibration curve, residuals vs. predictions, and confusion matrix) illustrating recommended evaluation for these pipelines

To make these concepts more concrete, Fig. 11 summarizes both exemplar pipelines and the associated diagnostics we advocate for AI-guided nanotheranostic models. The upper panels depict end-to-end workflows for six representative applications, from protein corona prediction and cell-specific uptake optimization to hybrid PBPK–ML tumor delivery models and imaging-driven dose selection, explicitly showing data inputs, feature engineering, core ML/AI components (including any mechanistic sub-modules), and the decision-relevant outputs and clinical use cases. The lower panels illustrate recommended diagnostic views on held-out or external data: a SHAP summary plot to identify influential features, a calibration curve to assess probability reliability, a residuals-versus-predicted plot to reveal bias and heteroscedasticity, and a normalized confusion matrix for classification tasks. These panels are intended as didactic exemplars rather than new experimental results, and together they provide a practical blueprint for how future primary studies should report and interrogate AI models in nanotheranostics.

### AI for patient stratification and predictive response modeling

The advent of personalized medicine has accentuated limitations inherent in one-size-fits-all cancer treatment strategies, especially within the context of nanotheranostics, where the PKs of NPs and efficacies of therapies are both heavily influenced by individual patient factors [395, 396]. AI has emerged as a revolutionary solution to this complexity by translating vast and varied sets of biomedical data into actionable knowledge. Within the field of AI-based nanomedicine, stratification of patients using multi-omics and imaging profiles, and predictions of therapeutic responses have moved from a state of simple feasibility to one of greater precision [397–399].

Despite these advances, most current AI systems deliver stratified rather than truly individualized care. Models are typically trained on cohort-level data and learn associations that reflect the “average” patient in the training distribution, which can fail to capture the full impact of germline and somatic genetic variation, tumor-microenvironment interactions, organ dysfunction, polypharmacy, and complex comorbidities on nanoparticle pharmacokinetics and treatment response. In practice, sparsity of well-annotated outcome data for rare molecular subtypes, elderly and frail patients, or those with multiple coexisting diseases strongly limits the degree of personalization achievable by purely data-driven approaches. As a result, AI-guided nanotheranostic decisions often remain constrained to risk groups or response clusters, rather than robust “N-of-1” predictions for a given individual [400].

## From variability to precision: the role of AI

Cancer is inherently heterogeneous genetically, epigenetically, and phenotypically [401, 402]. This heterogeneity affects how tumors interact with nanocarriers, respond to drugs, and evolve during treatment [403]. AI algorithms, particularly those based on ML and DL, are well-equipped to handle the high dimensionality and complexity of biological data. These models can learn latent patterns that correlate with NP uptake, cellular uptake dynamics, immune infiltration, and therapeutic outcomes [404].

For instance, supervised ML algorithms using genomic and proteomic features can successfully predict the compatibility of NPs with specific tumor genotypes, thus enabling the subtyping of patients to improve the effectiveness of nanodrugs. A related example is the integration of support vector machines (SVMs) to predict the stability or dispersity of curcumin-loaded liposomes, with area under the receiver operating characteristic curve (AUROC) values exceeding 0.88 in validation cohorts (88% ± 5% with stratified *k*-fold cross-validation) [405].

## Omics-Informed classification for nanomedicine

Multi-omics datasets encompassing genomics, transcriptomics, proteomics, and metabolomics offer a comprehensive view of tumor biology [406, 407]. AI frameworks can integrate these heterogeneous data types via feature-fusion architectures, such as multimodal autoencoders and graph neural networks (GNNs), that consider both molecular abundances and molecular interaction frameworks [408].

For example, the use of transcriptomic clustering with unsupervised DL approaches has identified unique subgroups of lung cancer patients with differential expression levels of surface proteins interacting with NPs (e.g., scavenger receptors and integrins), thus modulating the efficacy of targeted NPs [409, 410]. Similarly, the use of AI in metabolomic profile analyses can enable patient stratification based on metabolic phenotypes, which are predictors of enhanced permeability and retention effects–critical determinants of passive NP accumulation [411, 412].

## Imaging-AI synergy for response forecasting

Besides molecular information, radiological and histopathological imaging also offers spatial views of tumor architectures and the microenvironmental conditions surrounding tumors [413]. AI-based algorithms, trained on MRI, PET, or CT imaging often utilize convolutional neural networks (CNNs) that are able to harvest radiomic features associated with vascular density, interstitial pressure, necrosis, and other attributes that are relevant to NP penetration and drug distributions [413, 414].

For example, deep CNNs trained with dynamic contrast-enhanced MRI [415] demonstrated high accuracy for predicting malignancy among the breast lesion models and achieved accuracy levels of over 90% in a slice-based method, while in a case-based method, the sensitivity exceeded 90%, with a specificity of nearly 60% and an accuracy higher than 80% [149, 416] DL models that incorporate diffusion-weighted imaging (DWI) were shown to have promise in predicting tumor responses to photothermal nanotherapies by modeling heat dissipation and temporal necrotic volume growth [417].

## Modeling the TME for therapeutic predictions

The TME, comprising immune cells, ECM, stromal fibroblasts, and the vasculature, profoundly influences therapeutic responses [418, 419]. AI-driven approaches have advanced the ability to dynamically map and model the TME, using both molecular and imaging data [420, 421].

By training models on single-cell RNA-sequencing (scRNA-seq) and spatial transcriptomics, researchers can predict the immune landscape of tumors and identify "cold" vs. "hot" TME profiles that are more or less responsive to nanocarrier-based immunotherapies [422, 423]. GNNs, in particular, have excelled at modeling intercellular relationships within the TME, predicting how stromal barriers or immune cell distributions may hinder or enhance NP access to tumor cores [424].

## Promoting predictive oncology by leveraging digital twins

Another pioneering application of AI to patient stratification is the development of digital twins–virtual replicas of individual patients that are programmed to simulate disease progression and treatment responses within a virtual setting [425, 426]. Such systems leverage real-time electronic health records, omics data, and imaging data to build multiple therapeutic paths, thus allowing healthcare providers to "evaluate" nanomedical strategies before their application in a clinical setting [427, 428]. AI-enhanced stratification and predictive modeling must no longer be viewed as ancillary tools but are ever more central to the success of AI-incorporated nanotheranostics. By enabling precise patient selection, prognostication, and dynamic therapy strategies, AI is revolutionizing prospects for individualized cancer therapy [429, 430]. The integration of omics, imaging techniques, and advanced AI platforms reflects a shift from conventional empiricism to data-guided methodologies in nanomedicine, furthering our quest for predictive, adaptive, and ultimately curative therapeutic paradigms [431, 432].

### Digital twins in cancer nanomedicine

The concept of digital twins–computer-simulated models of specific patients that mimic biological and physiological processes in real-time–can emerge as an innovative paradigm in the field of precision oncology [433]. By combining AI with patient-specific data streams, digital twins enable in silico simulations of disease progression and therapeutic interventions, such as those involving NPs. For the field of cancer nanomedicine, digital twins offer an unprecedented opportunity for personalizing treatment, optimizing dosing schedules, and predicting responses and side effects in advance, thereby revolutionizing clinical applications of nanotheranostics.

## From static models to dynamic patient avatars

Traditional modeling methods applied to nanomedicine often rely on static parameters derived from population-level research, which cannot capture the variability seen among individual patients [224]. In contrast, digital twins are dynamic systems governed by real-time or longitudinal streams of data covering a broad range of information, including multi-omics, imaging, and physiologic and behavioral data from wearable sensors. Such models are automatically updated by AI-driven algorithms, which learn and improve predictions over time, creating a living model of the patient that evolves with disease development and treatment responses [434, 435].

Digital twins have been used in cancer nanomedicine to simulate the PKs and pharmacodynamics (PDs) of NPs by considering factors like tumor size, vascular permeability, lymphatic clearance, and immune reactions. Surrogate modeling techniques, driven by AI, like Gaussian process regression and physics-informed neural networks, allow these computer simulations to realistically mimic complex biological processes while keeping computational costs low.

## Therapeutic simulations and optimization

A key role of digital twins in the field of nanotheranostics is their ability to forecast the efficacy of different therapeutic regimens using unique NP configurations, routes of administration, and dosing schedules before any concrete intervention is implemented. For instance, reinforcement learning algorithms incorporated into digital twins can simulate many treatment paths, analyzing outcomes with respect to tumor reductions, side effects, and progression-free survival durations, in turn suggesting an optimal treatment regimen [224, 436, 437].

## Adaptive therapy via real-time feedback loops

The therapeutic potential of digital twins mainly lies in their promise to enable adaptive therapy. By integrating real-time patient data, e.g., levels of circulating tumor (ct)DNA, real-time imaging data, or data derived from wearable biosensors, digital twins allow dynamic modifications of treatment regimens [377, 426]. AI algorithms embedded in a twin system can detect early signs of treatment resistance or poor responses, thus prompting the need for therapy changes, such as redesigning NPs, adding other co-therapies, or changing treatment timing.

## Challenges and potential directions for digital twins

**Fig. 12:**
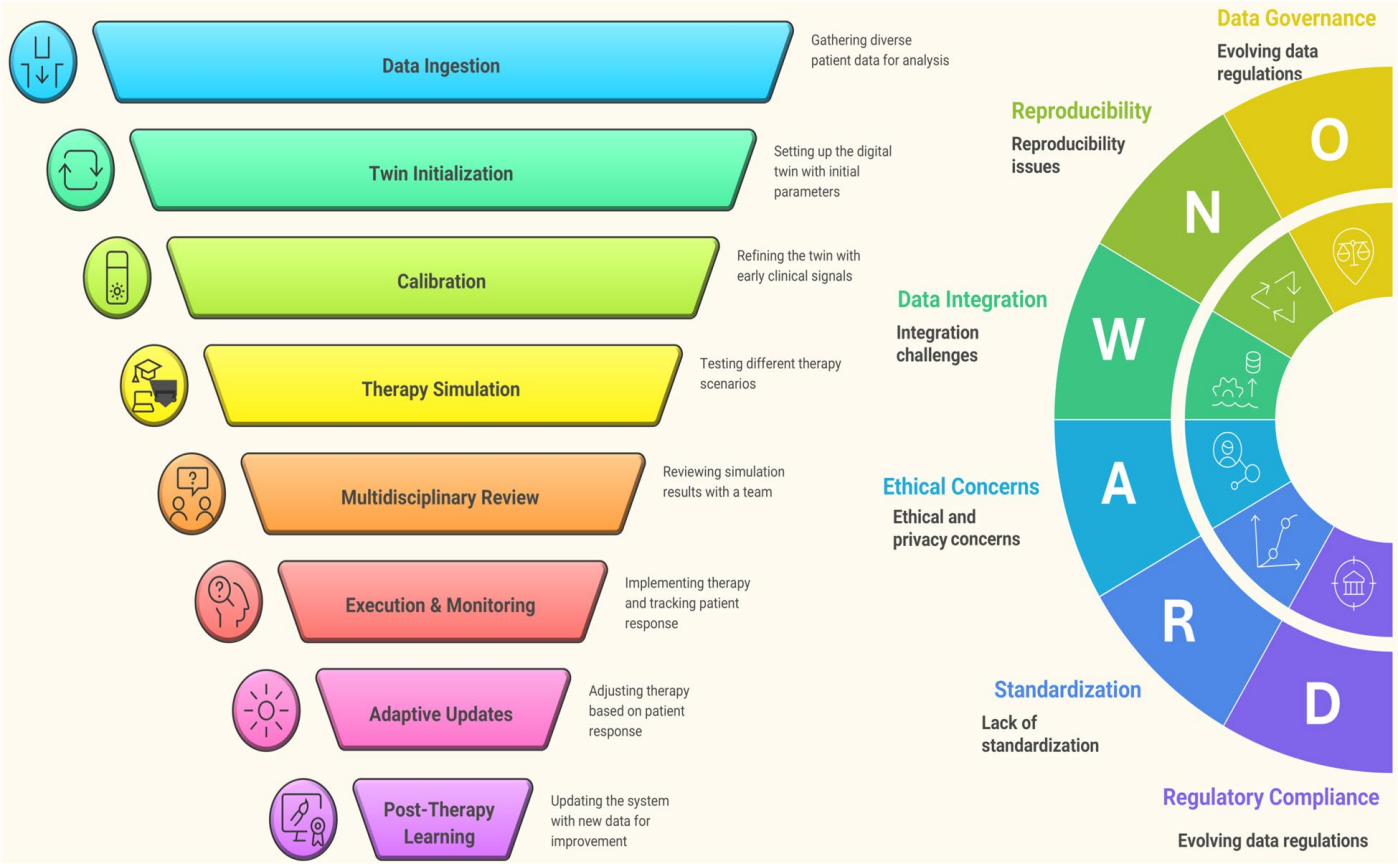
Clinical Workflow for AI/ML-Enabled Digital Twins and the ONWARD Framework for Nanotheranostics Deployment

Despite their promise, adoption of digital twins in cancer nanomedicine faces many challenges [438]. The first challenge relates to data integration, since most current clinical data are still compartmentalized or unstandardized across modalities [439]. The development of FAIR (findable, accessible, interoperable, reusable) data infrastructures is critical for the successful scaling of digital twin implementation [440]. The next challenge relates to model interpretability, as clinicians need to understand and trust decisions generated by AI, especially when recommending unusual NP formulations or off-label drug-nano combinations [441]. To address such issues, hybrid explainable AI (XAI) architectures are being incorporated into digital twin systems [442]. They use either rule-based or attention-based architectures to highlight patient features that have the most critical effect on a treatment decision, enabling the linkage between black-box predictions and clinical confidence [443, 444]. Additionally, multi-institutional consortia are now piloting digital twin registries for model validation and regulatory acceptance, with regulatory authorities like the EMA and FDA initiating initial frameworks for approving in silico clinical trials [445–447]. Digital twins represent the fusion of nanomedicine, AI, and systems biology into a unified tool for personalized cancer therapy Fig. 12, [448]. Patient avatars allow modeling of nanotherapy responses, promote adaptive treatment strategies, and enhance real-time clinical decision-making, aimed toward abolishing the trial-and-error strategy prevalent in traditional cancer therapies [449, 450]. As digital twin platforms mature while being supported by a foundation of explainability, interoperability, and strict validation, they are well-positioned to define the underlying paradigm for AI-augmented nanomedicine [451, 452].

## Challenges and limitations

**Fig. 13:**
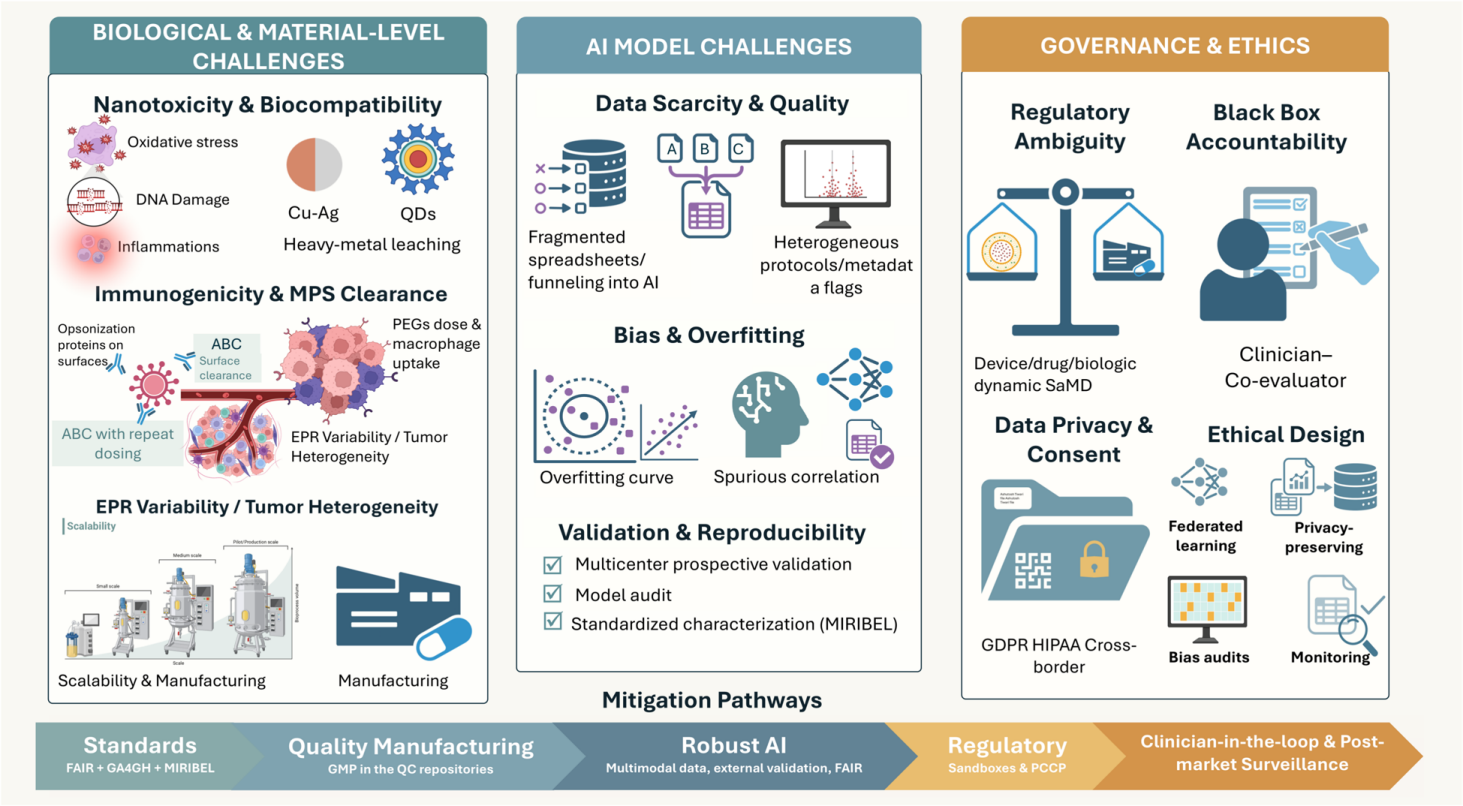
Challenges and limitations of AI-integrated nanotheranostics. Key barriers span (i) biological and material-level challenges, including nanotoxicity, immunogenicity, and EPR variability; (ii) AI model challenges, such as data scarcity, bias, and reproducibility; and (iii) governance and ethics issues, including regulatory ambiguity, black-box accountability, and privacy concerns. Mitigation pathways emphasize robust standards, quality manufacturing, multimodal validation, regulatory sandboxes, and clinician-in-the-loop frameworks for safe translation

The integration of AI with nanotheranostic systems has the potential to reshape the future of cancer diagnosis and treatment, yet its current applications are beset by fundamental challenges that hinder seamless clinical integration [453, 454]. These challenges Fig. 13, are multifaceted and diverse, ranging from basic issues of data quality and NP safety to higher-level concerns of explainability, regulatory ambiguity, and health equity [455]. As AI models continue to grow more sophisticated, their trustworthiness is inextricably linked to data quality and assumptions that feed them, yet much of the available nanomedicine data are marked by sparsity, heterogeneity, or discrepancies in experimental results [4]. At the same time, biological complexities underlying nano-bio interactions, especially in the context of the TME, introduce elements of unpredictability that are difficult to effectively model with contemporary computational tools [456, 457]. Additionally, the lack of transparency of many AI systems further hinders their integration into clinical workflows, where interpretability and accountability are of paramount importance [458, 459]. The regulatory infrastructure necessary to underpin such hybrid systems is still in the process of being developed, while ethical concerns regarding patient safety, data privacy, and algorithmic bias continue to be increasingly common [380, 460, 461]. In this section, we explore these pressing limitations in depth, providing critical perspectives through which to assess both the readiness and potential pitfalls of AI-augmented nanomedicine in oncology.

### Biological and material-level challenges

Despite the significant potential offered by AI-augmented nanotheranostics, the clinical deployment of NP-based platforms is still hindered by a number of major biological- and materials-related issues [462]. Foremost among these are concerns with nanotoxicity, immunogenicity, scalability, and the inherent heterogeneity of tumor biology–factors that make it difficult to achieve therapeutic efficacy and guarantee patient safety [155].

## Nanotoxicity and biocompatibility

One of the most important and ongoing issues in the area of nanomedicine is related to the possible toxicological impacts of nanomaterials [155]. Even if most NPs are engineered with a focus on biocompatibility, unforeseen interactions with cell and subcellular components can cause harmful consequences like oxidative stress, genotoxicity, and inflammation [463]. For instance, AuNPs, generally considered to be inert, can show size- and shape-dependent toxicity, especially if surface functionalization is poor or when these particles become concentrated in organs like the liver and spleen [76, 464]. QDs, particularly those with cadmium or selenium, are of even greater concern because of the possibility of heavy-metal leaching, despite their excellent imaging characteristics [465, 466].

AI algorithms based on available toxicological data have been used to predict cytotoxicity profiles through in silico approaches [467]; however, these models often struggle with a deficiency of high-resolution, context-dependent biological information [468]. Predictions are negatively impacted when NPs are placed in complex microenvironments such as tumors, which involve complex cell-to-cell contact, the ECM, heterogeneous oxygen levels, and the presence of immune cells [469]. Thus, the persistent issue of accurately predicting the nano-bio interface is a major limitation.

## Immunogenicity and immune evasion

Despite attempts to reduce toxicity, the aspect of immunogenicity still shows variability [470]. Unintentional activation of immune recognition by NPs can occur via opsonization, causing rapid clearance by the mononuclear phagocyte system (MPS) or, in certain cases, cause systemic hypersensitivity reactions [471, 472]. While attempts like PEGylation [314] and surface charge modifications are commonly utilized to reduce immune recognition, the process known as "accelerated blood clearance" (ABC) typically occurs with repeated dosing [473, 474]. Moreover, the immune system’s response to nanomaterials is highly individualized, influenced by genetic polymorphisms, comorbidities (preexisting health conditions, such as autoimmune diseases, allergies, or chronic infections), and prior exposure [475, 476]. AI can assist in identifying immunogenic patterns from patient-specific data, but the training of such models requires vast, immunophenotypically annotated datasets, which the field currently lacks [477, 478].

## Tumor Heterogeneity and the Enhanced Permeability and Retention (EPR) Paradox

Tumor heterogeneity is a major hurdle. The EPR effect, historically considered a basic principle for the passive targeting of NPs, is presently increasingly recognized to vary with different tumor types, stages, and indeed with different lesions within the same patient [388, 479]. Variables like the stromal density, vascular permeability, and lymphatic drainage show significant heterogeneity, thus undermining the ability to predict NP accumulation [480, 481]. AI-driven biodistribution models have attempted to forecast EPR efficiency using imaging and omics features [33, 149], yet the variability in intra-tumoral architectures renders such predictions unreliable without continuous data integration [22]. This variability demands the development of adaptive, feedback-driven delivery systems–an area where smart nanomaterials and digital twins may eventually offer solutions.

## Scalability and manufacturing bottlenecks

Besides biological constraints, material-level challenges have greatly retarded the process of clinical translation [482]. Many methods of fabricating NPs face scalability issues while maintaining uniform size, charge, drug loading, and functionalization. Batch-to-batch variability undermines quality control and raises regulatory concerns [483]. More-complex hybrid NPs, like core–shell structures or stimuli-sensitive release systems, tend to require multistep fabrication processes that yield low amounts, and which are unsuitable for good manufacturing practices (GMPs) [484]. AI-guided synthesis optimization and robotic high-throughput screening platforms offer partial solutions, accelerating formulation discoveries and process standardization [485]. However, their implementation is currently limited to research settings, and industry-wide adoption will require robust validation, cost reductions, and integration into pharmaceutical manufacturing pipelines [486].

### AI Model challenges

While AI has emerged as a major factor in designing and applying nanotheranostic platforms, its inclusion in clinical decision-making is constrained by a variety of algorithmic and data-related challenges [487, 488]. These challenges involve a lack of available data, sampling bias concerns, concerns over overfitting, low generalizability, and a long-term lack of interpretability concerns that are very much highlighted in the high-stakes arena of oncology, where decision-making often carries deep implications for patients’ lives. A further and often under-appreciated risk is that AI recommendations may become outdated, despite proper initial training. Changes in care standards, imaging, meds, or eligibility criteria create “concept drift,” where the relationships learned by the model no longer reflect current practice can cause the AI's knowledge to not reflect current practices. Changes in patient types can degrade a model's performance, causing it to give bad advice. Without monitoring and updates, AI tools in cancer care could spread outdated suggestions that don't match current guidelines [489, 490].

## Data scarcity and quality issues

AI models, particularly those within DL paradigms, inherently depend on large datasets [491]. In the context of nanomedicine, there is a significant lack of comprehensive and well-annotated datasets correlating the physicochemical properties of NPs with therapeutic outcomes [492, 493]. Much of the current data are scattered throughout numerous studies, show heterogeneity in experimental methods, and often lack the necessary detail to enable the training of predictive or generative models [494]. For instance, while databases like PubChem and ChEMBL offer detailed molecular-level annotations, very few databases comprehensively document NP-specific properties, such as the zeta potential, polydispersity, and in vivo biodistribution under controlled conditions [495, 496]. In addition, adverse outcomes vital to the prevention of spurious correlations and the enabling of strong model training are insufficiently represented within the current literature, leading to survivorship bias. This underrepresentation poses substantial challenges to supervised learning [497, 498], where the effectiveness and diversity of labeled examples are intrinsically tied to model success. Researchers are exploring semi-supervised and transfer learning strategies as potential solutions to these challenges [499, 500]. However, these methods are equally reliant on the consistency of domain-specific traits, which remains insufficiently standardized within the field of nanomedicine.

## Bias and overfitting

Bias in training datasets can significantly compromise a model's capability for generalization and fairness [501, 502]. In nanomedicine, datasets are usually derived from preclinical animal studies or small human cohorts that do not represent the full range of human physiological conditions, tumor types, or treatment regimens [503]. As a result, models trained under such limitations can demonstrate robust performance in silico or in comparison with chosen validation datasets, yet they often display poor performances when transferred into larger or different clinical settings. Overfitting is a common issue, often occurring in situations where high-dimensional NP descriptors are combined with small sample sizes [504, 505]. Without careful regularization or cross-validation, models can easily incorporate noise or spurious correlations, leading to inflated accuracy metrics that are not replicable in future research efforts [506, 507]. This problem is particularly troubling in the field of inverse design, where overfitted models can suggest NP structures that, while theoretically optimal, are biologically unrealistic.

## Lack of interpretability and clinical confidence

The absence of transparency that plagues many AI models, especially deep neural networks (DNNs), is a major hindrance to their use in clinical environments [508]. This is especially so in the arena of oncology, where the development of treatment plans requires transparency, traceability, and accuracy [509, 510]. Cryptic predictions of complex models are not likely to command the trust of practicing clinicians or gain regulatory approval. For instance, with a model that suggests a particular NP design for a glioblastoma patient [511], oncologists need to understand the underlying rationale: which input variables took precedence, if the suggestion was based on similar past cases, and what the expected therapeutic implications might be [512, 513]. Explainable AI (XAI) initiatives, such as SHAP (SHapley Additive exPlanations) [514], LIME (Local Interpretable Model-Agnostic Explanations) [515], and saliency maps, have shown promise in the fields of molecular studies and imaging [516, 517]. However, their application in the interdisciplinary field of nanomedicine, with its wide range of data types covering structural chemistry, imaging, and patient-specific omics, is still underexplored. In addition, the existence of explainability does not necessarily provide scientific rigor, especially when models uncover associations that lack a mechanistic foundation [518].

## Validation gaps and reproducibility

Even when AI models appear promising in preclinical simulations, validation against robust clinical datasets remains sparse [519]. Regulatory-grade validation requires prospective multicenter trials and also model auditing, sensitivity analyses, and transparency in training processes, elements often missing in early-stage AI-nanomedicine studies [520]. Moreover, reproducibility is frequently undermined by a lack of standardization in NP characterization methods, inconsistent metadata reporting, and proprietary preprocessing pipelines that limit the reproducibility of published findings [521].

To bridge this gap, initiatives like the NanoCommons Knowledge Infrastructure [522] and the Minimum Information Reporting in Bio-Nano Experimental Literature (MIRIBEL) [523] guidelines are presently being promoted; however, their deployment at the global level remains limited.

### Governance challenges and ethical issues

The convergence of AI with nanomedicine embodies a new era in oncology while simultaneously being confronted with a fragmented and often obsolete regulatory landscape [524]. Regulators, such as the US Food and Drug Administration (FDA) and the European Medicines Agency (EMA), are actively involved in adapting regulatory frameworks to more effectively accommodate innovative therapeutic modalities and digital technologies [525]. Yet current guidelines remain insufficiently equipped to evaluate hybrid systems that combine nanoscale materials with AI-driven decision-making frameworks. The existence of these regulatory gaps, coupled with ongoing ethical challenges, presents significant challenges for the clinical translation of AI-augmented nanotheranostics [21].

## Ambiguities in nanomedicine regulation

The integration of AI into nanomedicine management or design paradigms adds further complexity. Adaptive AI models that adjust drug-release profiles or personalize dosing based on real-time signals are inherently dynamic, challenging traditional static approval paradigms. Under current frameworks, AI components used to guide diagnosis or therapy are generally classified as high-risk SaMD, which requires pre-specification of algorithm versions, traceability of training data and updates, and prospective validation on independent cohorts. For AI-guided nanomedicine, these software requirements must be satisfied in addition to the quality, safety, and pharmacokinetic standards applied to the nanoparticle product itself, effectively creating a combined SaMD-plus-drug/device regulatory pathway [526]. Their sophisticated structure, combining inorganic carriers, biologically targeted ligands, and therapeutic entities, mandates careful safety, efficacy, and PK evaluations. While over 10 years of experience with the regulation of NP-based medicines such as Doxil® and Abraxane® have accumulated, the regulatory framework for innovative nanotherapeutics remains reactive instead of proactive. To date, there is no full regulatory precedent for an AI-guided nanoparticle therapy protocol; existing approvals involve either nanomedicines without AI or AI tools without nanomedicine. Consequently, regulatory approval for AI-enabled nanotheranostics will depend on prospective, multi-center clinical studies that demonstrate improved outcomes over standard care, not on in silico simulation or rodent models alone. The integration of AI into management or design paradigms brings great complexity. For example, adaptive AI models utilized to improve drug-release kinetics or determine personalized dosing for patients are inherently dynamic, making them complex to verify by traditional static trial design practices. The regulatory standards defined by the US FDA for SaMD and their proposed Predetermined Change Control Plans (PCCP) for AI systems represent progress; however, these standards have not yet been fully defined in the context of AI-based nanomedicine, where simultaneous changes can be made in both material properties and algorithmic performance [527].

## The ‘Black Box’ problem and clinical accountability

An important ethical issue relates to the opacity inherent in many AI architectures, particularly DNNs, that often behave like "black boxes" with limited interpretability [528]. In clinical oncology, where decisions can greatly influence life or death, an array of stakeholders, including oncologists, patients, and regulatory agencies, demands transparency and understandability of the outputs generated. If an AI system suggests a specific nanotherapeutic formulation or predicts a patient's prognosis from multi-omics information, it is essential that clinicians examine that suggestion: evaluating the underlying reasons, comparing it to prior cases, and elucidating the uncertainties involved. With no interpretability, the notion of accountability becomes vague. If a patient suffers adverse effects due to an AI-supported treatment, it becomes very difficult to determine liability whether of the doctor, software engineer, or nanomaterials manufacturer from both a legal and ethical point of view. Such complexities highlight the need for intrinsic explainability and traceability capabilities in any AI product used for theranostic therapy.

## Data protection, authorization, and regulatory frameworks

The effectiveness of AI-based personalized nanomedicine largely depends on the availability of extensive, high-resolution patient-derived datasets that include genomic, imaging, PK, and real-time physiological data [529, 530]. However, the processes associated with the acquisition, storage, and use of such sensitive data raise considerable ethical challenges. Even if datasets are de-identified, the integration of multiple modalities can potentially enable re-identification of patients, especially in cases of rare cancers or small population groups [530, 531].

Regulatory frameworks like the General Data Protection Regulation (GDPR) in the European Union [532] and the *Health Insurance Portability and Accountability Act* (HIPAA) in the US set basic protocols [533], but their application is often uneven, and the transnational flow of data remains murky. In addition, procedures for informed consent have fallen behind the evolution of AI technologies. Patients are often unaware of the use of their data for model training, the degree of autonomy ceded to these models within the framework of their treatment, or the commercial uses that can be derived from their data. Limits on accessing and linking data impact personalization. If detailed genomic and health data come from few places, AI models might not accurately represent patients with unusual biology or complex medical histories. These are the patients who would gain the most from custom treatments. Therefore, protecting privacy, managing data, and ensuring fair data collection are vital for AI to achieve truly personalized cancer care instead of just group predictions. [534, 535].

## Promoting ethical-by-design nanomedicine

Addressing these barriers will require coordinated efforts across regulatory bodies, developers, clinicians, and ethicists. Ethical-by-design principles must be embedded early in the development lifecycle of AI-integrated nanotheranostics [536]. This includes transparency in algorithm development, clear data provenance, privacy-preserving computations (e.g., federated learning), bias auditing, and post-market monitoring of both AI and nanomaterial performances. Algorithmic discrimination should be a primary safety concern, not a secondary one. AI training data for nanotheranostics often lacks representation from diverse groups, tumor types, and care environments. This leads to poorer performance in those populations, despite good overall results. Variations in scanners, protocols, or treatments can worsen these inequalities if models are mainly trained on data from large centers. Therefore, ethical development needs regular reporting of subgroup performance, bias checks, and fixes like re-weighting or domain adaptation, before clinical use. These concerns are echoed in our SWOT (Strengths, Weaknesses, Opportunities and Threats) analysis (Table 4, threats T2–T4), where data-sharing constraints, reproducibility issues, and equity gaps are identified as central governance challenges for AI-enabled cancer diagnosis and treatment. [537]. Also, ethical nanotheranostic AI governance needs continuous lifecycle management, not just single deployments. This includes retraining when guidelines or protocols change, routine data checks, and safe defaults when the AI isn't sure or data is unusual. High-risk advice should still be checked by a doctor, with the AI showing uncertainties and limits to avoid bad suggestions. In addition, coordinating international regulatory frameworks and creating settings for the testing of AI-nano systems in regulated clinical simulations can accelerate responsible innovations while maintaining public trust [538].

**Table 4:** SWOT analysis of AI-driven nanotheranostics

Strengths	Weaknesses	Opportunities	Threats
11. TDMM framework tying tasks to decision-relevant endpoints; 21. worked exemplars with datasets, baselines, and quantitative metrics; 31. translation focus on SaMD co-diagnostics, monitoring, and explainability; 41. Emphasis on uncertainty calibration and decision-curve (net-benefit) analysis; 51. Policy-compliant, technically corrected vector figures and standardized typography 61. reporting checklists/model cards to improve reproducibility	11. Heterogeneous evidence base (assays, scanners, protocols) complicates synthesis 21. Limited multi-site clinical validation 31. Many results are preclinical or single-site 41. sparse, non-standardized nano descriptors (e.g., protein corona features) 51. Few head-to-head benchmarks across common manifests; 61. inverse-design results often lack prospective laboratory validation 71. Drug-side regulatory pathway for AI-designed nano products remains unclear	11. Federated/privacy-preserving learning across centers for imaging/PK/digital twins 21. Standardized nano-informatics schemas and precompetitive benchmark challenges 31. Hybrid PBPK–ML models and physics-informed surrogates for PTT/PDT dose planning 41. High-throughput microfluidics with Bayesian optimization to accelerate inverse design 51. Multimodal foundation models (WSI/MRI/PET/PAI + omics/ctDNA) for richer signals 61. Adaptive trials using MRD/ctDNA and imaging to personalize dose/schedule	11. Regulatory tightening on adaptive AI (post-market change control, drift limits) 21. Data-sharing/privacy constraints and cross-border restrictions 31. Reproducibility risks from assay/scanner drift and manufacturing/scale-up variance 41. Bias and equity gaps if subgroup performance is not measured and reported 51. GMP/scale-up variability that breaks model assumptions between R&D and production 71. Hype backlash if claims outpace prospective validation or policy compliance

### SWOT analysis of AI-driven nanotheranostics

This SWOT synthesis (Table 4) positions our framework within the current translational landscape. Strengths include the TDMM structure that maps each algorithm to a decision-relevant endpoint, worked exemplars with baselines and calibrated metrics, and a clear SaMD-oriented translation path with monitoring and explainability. Weaknesses center on heterogeneous evidence (assays, scanners, protocols), limited multi-site clinical validation, sparse standardized nano descriptors (e.g., corona features), scarce head-to-head benchmarks, and unclear drug-side regulatory routes for AI-designed products. Opportunities lie in federated learning across institutions, standardized nano-informatics and shared manifests, hybrid PBPK–ML and physics-informed surrogates for dose planning, high-throughput microfluidics with Bayesian optimization for inverse design, multimodal foundation models, and adaptive trials using imaging and ctDNA. Threats include tightening regulation of adaptive AI, data-sharing constraints, assay/scanner drift and manufacturing variability that erode reproducibility, equity gaps without subgroup reporting, and reputational risk if claims outpace prospective validation. Together, this analysis motivates the concrete measures we propose-reporting checklists, external validation, drift sentinels and change control, subgroup/bias audits, and prospective lab validations for inverse-design claims which we operationalize in the subsequent Future Directions section.

## Future directions and opportunities

The confluence of nanomedicine and AI promises a revolution in cancer therapy through the possibility of developing AI-augmented theranostics, thus progressing toward a new paradigm of predictive, preventive, and precision oncology [539]. However, beyond algorithmic excellence and the development of novel nanomaterials, deeper disruptive breakthroughs are needed across the realms of science, clinical evaluation, regulation, and ethics to realize this great potential [15, 540]. The increasingly widespread availability of high-resolution multi-omics data, combined with advancements in in silico modeling platforms and the development of scalable manufacturing methods, is increasingly setting the stage for complex and adaptive theranostic systems [541, 542].

**Fig. 14:**
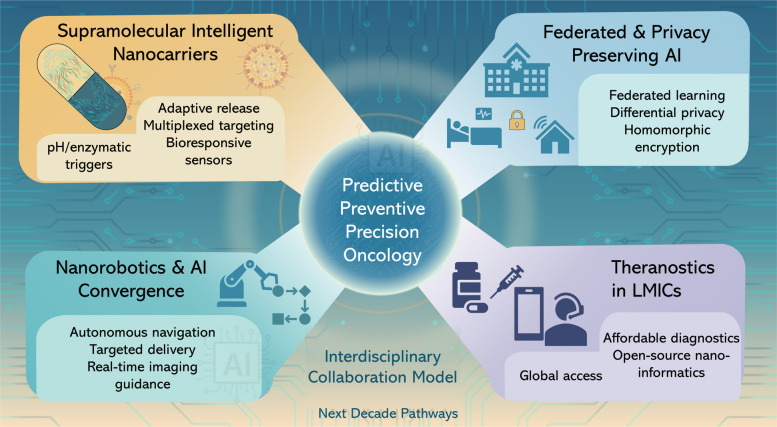
Future directions of AI-integrated nanotheranostics. Next-decade pathways emphasize interdisciplinary collaboration to achieve predictive, preventive, and precision oncology. Key innovations include supramolecular nanocarriers with adaptive and bio-responsive release, convergence of nanorobotics and AI for real-time guided delivery, federated and privacy-preserving AI to enable secure global data sharing, and expanding theranostic access in low- and middle-income countries through affordable diagnostics and open-source nano-informatics

Future directions Fig. 14, need to address particular shortcomings identified in the current translational pipeline. These shortcomings include the development of XAI architectures tailored for nanomedical use, robust data infrastructure to support sustained patient monitoring, and sophisticated nanocarriers with multifunctional properties to cater to various TMEs [543, 544]. Furthermore, the emergence of digital twins, the adoption of federated learning to support privacy-aware model training, and the ability to develop 4D-bioprinted theranostic platforms indicate a near-future transition towards autonomous, personalized treatment cycles [545, 546].

In this section, we outline the most promising technological and clinical areas expected to define the next decade of AI-based nanomedicine. It provides a strategic map for researchers, developers, and policymakers to work together in creating a more agile, equitable, and efficient era of cancer care.

### Supramolecular Intelligent Nanocarriers

The next breakthrough in nanotheranostics lies in the design of smart nanocarriers with the ability to dynamically respond to the TME while enabling real-time control over therapeutic interventions [547, 548]. Such "smart" systems integrate AI-powered feedback loops to sense, process, and respond to molecular cues in situ thus controlling drug release based on conditions such as pH gradients, enzyme activities, or biomarker levels [549, 550]. For instance, the use of deep reinforcement learning was proposed to optimize release kinetics by simulating diverse PK environments [551]. In addition, multiplexed targeting whereby multiple ligands are used to navigate complex tumor heterogeneity can benefit from AI algorithms that optimally mix parameters of affinity, avidity, and immune evasion in silico [552]. Finally, the use of onboard biosensors and bioresponsive elements, such as thermo-triggered or magnetically triggered release gates, offers a degree of spatial and temporal specificity that is unmatched by conventional drug-delivery systems [553, 554]. Realization of such autonomous nanodevices will require advances in the design of embedded nanosensors, low-power computation platforms, and AI models trained on empirical biodistribution data.

### Federated and privacy-preserving AI models

One of the main barriers to AI integration in nanomedicine is the fragmentation of high-quality clinical data due to privacy concerns, institutional constraints, and regulatory restrictions [527, 555]. Federated learning as a decentralized AI paradigm offers a revolutionary solution. With this approach, models are locally trained on data drawn from different hospitals or research centers while raw data are not shared, hence protecting patient confidentiality and enhancing generalizability [556–558]. Federated AI frameworks have a special importance in oncology, where individuality of patients frequently hinders the efficacy of AI generalization in the conventional sense. In addition, when working in conjunction with methods like differential privacy and homomorphic encryption, international collaboration in developing and evaluating nanotherapeutic AI models can be enabled without compromising data sovereignty.

Cancer AI systems can face data leakage in several ways, even with removed identifiers. Modalities like whole-slide images and multi-omics profiles can still reveal patient identities, especially when combined with external data. Model-level attacks can also expose sensitive information about a patient's involvement in model training or training samples from model outputs. Federated learning and similar methods such as differential privacy, and homomorphic encryption lower these risks by limiting data movement and information leakage. Still, they don't eliminate them fully and may affect model utility and system complexity. Thus, using AI in oncology requires careful threat modeling and security checks, instead of relying only on technical safeguards to ensure privacy [558, 559].

### Nanorobotics and AI convergence

Concepts such as AI-integrated nanorobots are often cited as a visionary end point of nanomedicine, but they should currently be regarded as long-term, preclinical research directions rather than foreseeable clinical technologies [560]. By employing swarm intelligence, autonomous navigation strategies, and real-time image guidance feedback, nanorobots ensconced with AI would have the ability to execute complex tasks, such as navigating vascular spaces, delivering drugs in situ, or removing biospecimens from the TME [560]. Albeit currently a largely theoretical concept, advances in magnetically actuated nano-swimmers and stimulus-responsive nanomachines are creating a basis for such developments [561, 562]. The function of AI is central to assuring real-time decision-making in the context of uncertain physiological states that balance exploratory and exploitative actions while adjusting behaviors according to nascent pathological indicators [563–565]. However, there are no AI-guided nanorobotic cancer therapies in clinical use or in human trials, and multiple fundamental barriers remain unresolved: biocompatible and scalable energy sources, robust communication and localization deep in tissue, safe degradation or retrieval, reliable behavior control in complex hemodynamic and immunological environments, and an appropriate regulatory framework. Accordingly, nanorobotics is discussed here only as a speculative convergence area at the interface of AI and nanomedicine; it should not be interpreted as a near-term route to autonomous personalized nanoscale interventions in oncology. [566].

### Theranostics in resource-limited countries

In order to enhance global fairness in cancer therapy, it is necessary to focus on developing economically sustainable nanotheranostics that are tailored to low- and middle-income countries (LMICs) [567, 568]. The use of AI-augmented lateral flow assays, smartphone-facilitated biosensing systems, and cost-effective NP fabrication methods holds great promise in overcoming limitations to early cancer diagnoses [569, 570]. For example, mobile imaging systems with embedded ML algorithms have demonstrated high sensitivity in analyzing NP-enhanced fluorescence or colorimetric signals even in low-resource environments [571]. Additionally, the use of open-source nano-informatics tools and communal data aggregation frameworks can enhance access to precision cancer therapies in disadvantaged regions. Global health agencies, local biotech companies, and academic investigators must work together to tailor AI-nanomedicine advances to the unique requirements of different environments [572].

### Interdisciplinary collaboration models

Successful deployment of AI-integrated nanotheranostics in the clinic relies on creating sustainable ecosystems that engage multiple stakeholders [573]. The academic community brings forth advances in materials science and algorithmic development, the industrial community contributes expertise in large-scale production, regulatory compliance, and commercialization routes, and government agencies are essential for infrastructure, policy development, and ethical regulation [574, 575]. Collaborative frameworks, such as public–private translational consortia, cross-institutional innovation hubs, and shared data repositories, can accelerate the laboratory-to-clinic journey [576]. Prominent programs like the Cancer Nanotechnology Plan [577] in the US and the European Nanomedicine Translation Hub [578], demonstrate such collaborative structures [482, 579]. Additionally, developing standardization protocols, open-access platforms, and global benchmarking datasets will augment reproducibility and credibility in this rapidly evolving field [580].

## Conclusions

AI's arrival significantly changes how we approach cancer diagnosis and treatment in nanomedicine, creating a blend of precision at the nanoscale and patient-specific data use. AI-supported nanotheranostics offer great control in how drugs are given, how images are produced, and how the body reacts to treatment. This helps create multi-use nanoparticles designed for each patient's specific biological traits. These range from intelligent drug release to forecasting models for treatment results. The fusion of machine learning and designed nanomaterials marks a clear shift from typical, one-size-fits-all cancer treatments. This link goes past simple tech progress, holding considerable potential for direct practical use. Currently, many AI-driven nanoparticle systems are in early testing phases. Approved nanomedicines offer vital primary data, which help integrate AI into safety and efficiency evaluations. As this integration becomes more common in clinics, strict rules are a must. These rules should address not only the complex nature of nanomaterials physically and biologically but also the moral, legal, and computational parts of using AI in critical treatments. Currently, AI-enabled nanotheranostics should be positioned as a means to refine risk stratification, trial design, and treatment planning, while leaving ultimate decisions to clinicians and patients; AI cannot replace real-time symptom assessment, holistic evaluation of comorbidities and preferences, or the ethical responsibility inherent in clinical judgment. For the future, using clear, repeatable, and morally sound AI models across the board will be key to building trust among doctors, supervisors, and patients. Joint work between universities, businesses, and governments must focus on making compatible data standards, spread out AI training networks, and available platforms for model checking. Only through teamwork can we fully understand what AI-enhanced nanomedicine can do, transforming it from a specialized item into a major and fair power in the global fight against cancer.

## Supplementary Information

Supplementary Material 1.

## Data Availability

No datasets were generated or analysed during the current study.

## Abbreviations

ABC: Accelerated blood clearance
AI: Artificial intelligence
AuNP: Gold nanoparticle
CNN: Convolutional neural network
CT: Computed tomography
ctDNA: Circulating tumor DNA
CVAE: Conditional variational autoencoder
DL: Deep learning
DLT: Dose-limiting toxicity
EMA: European Medicines Agency
EPR: Enhanced permeability and retention (effect)
FAIR: Findable, accessible, interoperable, reusable
GAN: Generative adversarial network
GDPR: General Data Protection Regulation
GLP: Good laboratory practice
GMP: Good manufacturing practice
GNN: Graph neural network
HIPAA: Health Insurance Portability and Accountability Act
IVIVC: In vitro-in vivo correlation
LNP: Lipid nanoparticle
ML: Machine learning
MIRIBEL: Minimum Information Reporting in Bio-Nano Experimental Literature
MRI: Magnetic resonance imaging
MPS: Mononuclear phagocyte system
NIR: Near infrared
NIRF: Near-infrared fluorescence
NP: Nanoparticle
PAI: Photoacoustic imaging
PBPK: Physiologically based pharmacokinetic
PCL: Polycaprolactone
PDT: Photodynamic therapy
PEG: Polyethylene glycol
PET: Positron emission tomography
PKs: Pharmacokinetics
PLGA: Poly(lactic-co-glycolic acid)
PTT: Photothermal therapy
QD: Quantum dot
RL: Reinforcement learning
ROS: Reactive oxygen species
SaMD: Software as a Medical Device
SAR: Structure-activity relationship
SEM: Scanning electron microscopy
SERS: Surface-enhanced Raman scattering
SPR: Surface plasmon resonance
TEM: Transmission electron microscopy
TME: Tumor microenvironment
UCNP: Upconversion nanoparticle
US FDA: United States Food and Drug Administration
VAE: Variational autoencoder
XAI: Explainable artificial intelligence
