# Supplementary material for: AI-driven nanomedicine for cancer theranostics
Source: Mol Cancer. 2026 Feb 13;25:78. doi: 10.1186/s12943-025-02563-9 (PMC13005573; doi:10.1186/s12943-025-02563-9)
Supplement: Supplementary file 1 — Supplementary Material 1. [file 12943_2025_2563_MOESM1_ESM.pdf]

AI-Driven Nanomedicine for Cancer Theranostics

Ashutosh Tiwari[1], Widodo[2], Dyah Ika Krisnawati[3], Chih-Yu Chen[1,4,5,*], Tsung-Rong Kuo[1,6,7,*]

[1] International Ph.D. Program in Biomedical Engineering, College of Biomedical Engineering, Taipei Medical University, Taipei 11031, Taiwan.
[2] Institut Teknologi AI-Mahrusiyah, Kediri, 64112, East Java, Indonesia
[3] Department of Nursing, Faculty of Nursing and Minwifery, Universitas Nahdlatul Ulama Surabaya, Surabaya, 60237, Indonesia
[4] Department of Orthopedics, Shuang Ho Hospital, Taipei Medical University, 291, Zhongzheng Road, Zhonghe District, New Taipei City 23561, Taiwan
[5] School of Biomedical Engineering, Taipei Medical University, Taipei 11031, Taiwan
[6] Graduate Institute of Nanomedicine and Medical Engineering, College of Biomedical Engineering, Taipei Medical University, Taipei 11031, Taiwan.
[7] Precision Medicine and Translational Cancer Research Center, Taipei Medical University Hospital, Taipei 11031, Taiwan

* Corresponding author.
E-mail: aleckc2424@tmu.edu.tw(C.-Y.C); trkuo@tmu.edu.tw (T.-R.K.)

Supplementary Table 1. Nanomedicines Developed for Cancer Treatments

Name	Formulation / Composition	Indication / Application	Approval / Clinical Trial	New Cancer Types Being Explored	References
Doxil/Caelyx	PEGylated liposomal doxorubicin	Ovarian cancer, breast cancer, Kaposi's sarcoma, multiple myeloma	USA (1995), Europe (1996)	Glioblastoma, endometrial cancer	[1–6]
DaunoXome	Liposomal daunorubicin	Kaposi's sarcoma	USA (1996)	Leukemia variants, aggressive lymphomas	[7–13]
DepoCyt(e)	Liposomal cytarabine	Leptomeningeal metastasis	USA (1999), Europe (2001)	CNS leukemia	[14–21]
Myocet	Liposomal doxorubicin	Metastatic breast cancer	Europe/Canada (2000)	Lung cancer, sarcoma	[22–27]
Lipusu	Liposomal paclitaxel	NSCLC, ovarian, breast cancers	China (2003)	Gastric, pancreatic, cervical cancers	[28–32]
Abraxane	Albumin-bound paclitaxel	Breast cancer, pancreatic cancer, NSCLC	USA (2005), Europe (2008)	Bladder, esophageal, head and neck cancers	[33–36]
Nanoxel	Micellar paclitaxel	Breast, ovarian, NSCLC, Kaposi's sarcoma	India (2006)	Cervical, hepatocellular carcinoma (HCC)	[37–41]
Oncaspar	PEGylated L-asparaginase	Acute lymphoblastic leukemia (ALL)	USA (2006), Europe (2016)	Lymphoblastic lymphoma	[42–44]
Genexol PM	Micellar paclitaxel	Breast cancer, NSCLC	South Korea (2007)	Gastric, cervical cancers	[45–49]
Mepact	Liposomal mifamurtide	High-grade osteogenic sarcoma	Europe (2009)	Refractory bone cancers	[50–52]
NanoTherm	Iron oxide nanoparticles (NPs) (aminosilane-coated)	Glioblastoma (magnetic hyperthermia)	Europe (2011)	Prostate, head and neck cancers	[53–56]
Marqibo	Liposomal vincristine	Relapsed/Refractory ALL	USA (2012)	Aggressive lymphoid malignancies	[57–61]
Onivyde	Liposomal irinotecan	Metastatic pancreatic cancer	USA (2015), Europe (2016)	Colorectal (CRC), gastric cancers	[62–65]
Liporaxel	Lipid nanodroplets with paclitaxel	Breast, gastric, ovarian cancers	South Korea (2016), China (2024)	Triple-negative breast cancer (TNBC)	[66–68]
Vyxeos	Liposomal daunorubicin + cytarabine	Secondary AML	USA (2017), Europe (2018)	High-risk myeloid malignancies	[69–73]

Apealea	Micellar paclitaxel (XR17)	Ovarian cancer	Europe (2018)	Peritoneal carcinomatosis	[74–76]
Hensify	Hafnium oxide NPs	Soft-tissue sarcoma (radiotherapy enhancer)	Europe (2019)	Cervical cancer, head and neck cancers	[77–81]
ThermoDox	Thermosensitive liposomal doxorubicin	Liver, breast, pancreatic cancers (with thermal activation)	Multiple trials (2004–2021)	Bone metastases, HCC	[82–86]
AGuIX	Gadolinium-bound polysiloxane NPs	Radiosensitizer for glioblastoma, brain metastases	Phases I–II (Europe, 2016–2024)	Lung, head and neck cancers	[87–90]
FixVac (BNT111–116)	Lipid NPs with mRNA encoding tumor antigens	Melanoma, NSCLC, prostate, head and neck cancers (as therapeutic cancer vaccine)	Phases I–II (2015–2023)	HPV-positive cancers, gastric cancer	[91–96]
iNeST (BNT122 / RO7198457)	Personalized mRNA-loaded lipid NPs	Melanoma, pancreatic, NSCLC, CRC	Phases I–II (2017–2024)	High-risk urothelial carcinoma	[97–100]
V940 (mRNA-4157)	mRNA-loaded lipid NPs (Moderna–Merck)	Melanoma, NSCLC, bladder, renal cell carcinoma	Phases II–III (2017–2025)	HPV-associated cancers	[101–103]
CALAA-01	Transferrin-targeted polymeric NPs with siRNA	Refractory solid tumors	Phase I (2008, terminated)	KRAS-driven tumors	[104–106]
AuroShell	Silica-gold core-shell NPs for photothermal therapy	Head & neck tumors, prostate cancer (thermal ablation)	Phase I–II (2008–2020)	Bladder cancer	[107–110]
MM-302	HER2-targeted liposomal doxorubicin	HER2+ metastatic breast cancer	Phases I–II (2011–2014)	HER2+ gastric cancer	[111–114]
64Cu-MM-302	Radio-labeled HER2-targeted liposomal doxorubicin	HER2+ breast cancer (theranostics & imaging)	Phase I (2011–2016)	Theranostics for HER2+ cancers	[115–118]
Nab-rapamycin (ABI-009)	Albumin-bound rapamycin NPs	Advanced perivascular epithelioid cell tumors (PEComas)	Approved in USA, FYARRO	Metastatic malignant perivascular epithelioid cell tumor	[119]
CRLX101	Cyclodextrin-based NPs carrying camptothecin	NSCLC, renal cell carcinoma, rectal cancer	Phase II trials	Ovarian cancer, endometrial cancer	[120–123]
BIND-014	PSMA-targeted polymeric nanoparticles with docetaxel	Prostate cancer, NSCLC, breast cancer, HNSCC	Phases I–II (terminated)	KRAS-mutated CRC	[124–127]

NU-0129	Gold NPs loaded with siRNA (BCL2L12)	Glioblastoma	Phase 0 (first-in-human, 2019)	Brain metastases	[128–130]
MagForce NanoTherm	Superparamagnetic iron oxide NPs	Glioblastoma (magnetic field-induced hyperthermia)	Europe (2011)	Prostate cancer (clinical use in Germany)	[131–134]
Aptamer-conjugated NPs	Polymeric/lipid-based NPs conjugated with aptamers	Tumor-targeted drug delivery (preclinical)	Preclinical	Breast, pancreatic cancers, leukemia	[135–137]
SPION-Doxorubicin	Superparamagnetic iron oxide NPs (SPION) with doxorubicin	Localized breast cancer hyperthermia and chemo combo	Experimental (animal/human trials)	Cervical, liver tumors	[138–141]
Polymer micelle cisplatin (NC-6004)	PEG-polyglutamate micelles carrying cisplatin	Head and neck, pancreatic, bile duct cancers	Phase III	Mesothelioma	[142–144]
SNAs (spherical nucleic acids)	Gold NP core with DNA shell	Gene silencing, mRNA delivery, glioblastoma	Phases I–IIa (2017–2022, NU-0129 trial)	Multiple solid tumors	[145–149]
PEGylated SN-38 nanoliposomes (MM-398/Onivyde)	Liposomal irinotecan	Pancreatic cancer (approved)	Approved in USA/EU	Glioma, gastric cancers, CRC	[150–153]
HPMA copolymer–doxorubicin conjugate (PK1)	Water-soluble polymer-drug conjugate	Breast cancer, CRC	Phases I/II	Ovarian, head and neck cancers	[154–156]
ExoASO-STAT6	Exosomes loaded with ASO targeting STAT6	CRC, gastric cancer	Phase I (2022, terminated)	Immunotherapy targets for solid tumors	[157–159]
iExosomes	Exosomes carrying siRNA targeting KRASG12D	Pancreatic ductal adenocarcinoma (PDAC)	Phase I (ongoing)	NSCLC, colorectal cancers with KRAS mutation	[160–163]
THE001	Thermosensitive liposomal doxorubicin	Soft-tissue sarcoma	Phase I (2023), Orphan Drug Designation (2025)	Liposarcoma, leiomyosarcoma	[164–166]
NanoTax	Unbound paclitaxel NPs for intraperitoneal use	Ovarian cancer (locoregional delivery)	Phases I/II	Peritoneal metastases from gastric/colon cancers	[167–171]
TargomiRs	Bacterially derived minicells delivering microRNA mimics	Malignant pleural mesothelioma, NSCLC	Phase I (2016, Australia)	Pancreatic cancer, melanoma	[172–175]
DCR-MYC	Lipid NP siRNA targeting MYC	Solid tumors, lymphoma	Phase I (terminated)	Prostate cancer	[176–179]

Aurimune (CYT-6091)	PEGylated colloidal gold-TNF-alpha conjugate	Solid tumors (TNF delivery)	Phase I	Pancreatic, breast, head and neck cancers	[180–184]
MRX34	Liposomal miR-34a mimic	Liver, lung, lymphoma (first miRNA therapy in humans)	Phase I (terminated)	CRC, glioblastoma	[185–188]
BIND-510	PSMA-targeted docetaxel polymeric NP with kinase inhibitor	Castration-resistant prostate cancer	Preclinical	Brain metastases	[189–191]
HER2-targeted SNAs	Spherical nucleic acids (gold NP + DNA shell)	HER2+ breast cancer	Preclinical / Exploratory Trials	HER2+ gastric cancer	[192–194]
SP1049C	Pluronic block copolymer with doxorubicin	Esophageal carcinoma, gastric cancer	Phase II	Breast, testicular cancers	[195–198]
CPT-11-loaded H-ferritin NPs	Ferritin-based self-assembling carriers for irinotecan	Colon cancer	Preclinical	Pancreatic, lung, CNS cancers	[199–203]
NanoMBGs	Mesoporous bioactive glass (MBG) NPs	Bone cancer, osteosarcoma	Preclinical	Local chemotherapy for bone tumors	[204–206]
TfR-targeted carbon dots	Transferrin receptor (TfR)-targeted carbon-based nanodots	Glioma imaging and drug delivery	Preclinical	CNS tumors, metastatic brain cancer	[207–210]
C225-conjugated liposomes	EGFR-targeted liposomal carriers	Head and neck cancer	Preclinical / Phase I	EGFR+ colorectal, glioblastoma	[211–213]
Polymer-lipid hybrid NPs	Polymer core + lipid shell (e.g., PLGA-lipid hybrids)	Broad applications: breast, colon, ovarian cancers	Extensive preclinical use	Combination chemo-immunotherapy	[214–217]
Chitosan NPs with curcumin	Biopolymer nanocarriers	Chemo-sensitization in drug-resistant cancers	Preclinical	Oral, cervical, liver cancers	[218–223]
siRNA-PLGA-PEG NPs	Targeted gene silencing (e.g., VEGF, survivin)	Anti-angiogenic therapy for solid tumors	Preclinical	Ovarian cancer, melanoma	[224–226]
NK cell-loaded NPs	Nanocarriers functionalized with natural killer (NK) cells	Immunotherapy delivery to solid tumors	Proof-of-concept	TNBC, lung carcinoma	[227–230]
NU-0129 (SNA-based)	Gold NP + siRNA targeting BCL2L12	Glioblastoma multiforme (GBM)	Phase 0 (first-in-human)	Pediatric gliomas, brain metastases	[231,232]
DOXIL-BioMOF	Doxorubicin-loaded metal-organic framework (MOF)	Chemotherapy-loaded nanosystem for deep-tissue penetration	Preclinical	Hypoxic tumors (e.g., colon cancer), TNBC	[233,234]

AS1411 Aptamer–DOX NPs	DNA aptamer conjugated to doxorubicin-loaded NPs	Nucleolin-targeted delivery in breast, renal, colon cancers	Preclinical	AML	[235–238]
NanoHER2-TKI	HER2-targeted NP loaded with tyrosine kinase inhibitor (TKI)	HER2+ breast and gastric cancers	Preclinical	HER2-amplified NSCLC, bladder cancer	[239–245]
NanoGemcitabine (Gemcitabine-PLGA NPs)	PLGA-based controlled release of gemcitabine	Pancreatic, lung, bladder cancers	Preclinical	Intravesical bladder cancer, HCC	[246–248]
NOX66 (Idronoxil in NP form)	Radiosensitizer formulation for prostate/rectal cancer	Synergizes with radiation or checkpoint inhibitors	Phase II (NOX66-2, Veyonda trial)	Metastatic castration-resistant prostate cancer (mCRPC)	[249–252]
Polymersomes (oxaliplatin-loaded)	Amphiphilic polymer vesicles with platinum drug	CRC	Preclinical	Liver metastases of CRC	[253–255]
NanoCurcumin (polymeric)	PLGA or chitosan-encapsulated curcumin	Breast cancer, pulmonary metastasis, pancreatic cancer, human HCC	Investigational	Oral cancers, nasopharyngeal cancer, esophageal adenocarcinoma (EAC)	[256–261]
Iron oxide–paclitaxel (SPIONs)	Superparamagnetic iron oxide with paclitaxel	Localized chemo and magnetic hyperthermia, fibrosarcoma, NSCLC	Preclinical	Liver, breast tumors osteosarcoma	[262–266]
PNIPAM-based NPs	Thermoresponsive poly(N-isopropylacrylamide) micelle (PNiPAM), etoposide	Controlled drug release under mild hyperthermia	Experimental	Recurrent skin, prostate tumors	[267–269]
PEG-PLA-cisplatin NPs	Polyethylene glycol (PEG)-polylactic acid (PLA) loaded with cisplatin	Ovarian, cervical cancers	Preclinical	Radiation sensitization for head and neck cancers	[270–274]
DOX-SWCNTs	Doxorubicin (DOX)-loaded single-walled carbon nanotubes (SWCNTs)	High-dose localized chemotherapy with minimal systemic toxicity	Preclinical	Bone tumors, breast cancer	[275–277]
Nano-PDT (photodynamic NPs)	Photosensitizer-loaded NPs for light-triggered therapy	Head and neck, cervical, bladder cancers	Preclinical	Cutaneous malignancies, esophageal cancer	[278–280]
MSNs (mesoporous silica NPs)	Tunable nanopores carrying multiple drugs	Multidrug delivery for breast and colon cancer	Preclinical	Metastatic multidrug-resistant cancers	[281–285]

AML, acute myeloid leukemia; ALL, acute lymphoblastic leukemia; ASO, antisense oligonucleotide; CRC, colorectal cancer; CNS, central nervous system; DOX, doxorubicin; EAC, esophageal adenocarcinoma; EGFR, epidermal growth factor receptor; FDA, Food and Drug Administration; GBM, glioblastoma multiforme; HCC, hepatocellular carcinoma; HER2, human epidermal growth factor receptor 2; HNSCC, head and neck squamous cell carcinoma; HPV, human papillomavirus; LNP, lipid nanoparticle; mCRPC, metastatic castration-resistant prostate cancer; miRNA, microRNA; MOF, metal–organic framework; mRNA, messenger RNA; mTOR, mechanistic target of rapamycin; NK, natural killer (cell); NP, nanoparticle; NSCLC, non-small cell lung cancer; PDAC, pancreatic ductal adenocarcinoma; PEG, polyethylene glycol; PEGylated, surface-modified with polyethylene glycol; PEComa, perivascular epithelioid cell tumor; PK, pharmacokinetics; PLA, polylactic acid; PLGA, poly(lactic-co-glycolic acid); PSMA, prostate-specific membrane antigen; rRNA, ribosomal RNA; siRNA, small interfering RNA; SNA, spherical nucleic acid; SPION, superparamagnetic iron oxide nanoparticle; STAT6, signal transducer and activator of transcription 6; SWCNT, single-walled carbon nanotube; TfR, transferrin receptor; TKI, tyrosine kinase inhibitor; TNBC, triple-negative breast cancer.

Supplementary Table 2. High-profile cancer nanomedicine failures: proximate and systemic reasons

Product / platform	Primary indication(s) targeted	Phase where stalled or constrained	Proximate reason for failure / limited success	Underlying systemic barrier(s) illustrated	References
Doxil/Caelyx (PEGylated liposomal doxorubicin)	Metastatic breast, ovarian cancer, HIV-KS, others	Approved; multiple Phase III trials in new settings non-superior	Reduced cardiotoxicity but no consistent OS/PFS superiority vs conventional doxorubicin; benefits largely confined to tolerability and niche risk groups	Cost–benefit misalignment in health-technology assessments; regulators and payers prioritize hard survival/PRO gains over PK improvements alone; strong generic competition	[1–6]
Myocet (non-PEGylated liposomal doxorubicin)	Metastatic breast cancer	Approved/used in limited geographies; no broad displacement of SOC	Similar response and survival outcomes to generic doxorubicin with reduced cardiotoxicity but no transformative efficacy	Incremental benefit insufficient to drive global uptake; reimbursement constraints and entrenched familiarity with conventional anthracyclines	[22–27]
NC-6004 (polymer micelle cisplatin)	Solid tumors (e.g. lung, pancreatic)	Phase II/III – programs not taken to	Reduced nephrotoxicity but no compelling,	Crowded indications with evolving IO/chemo	[142–144]

Product / platform	Primary indication(s) targeted	Phase where stalled or constrained	Proximate reason for failure / limited success	Underlying systemic barrier(s) illustrated	References
		successful registration	practice-changing efficacy signal vs standard cisplatin regimens	combinations; incremental gains inadequate versus cheap generics; difficulty justifying premium pricing without survival advantage	
SP1049C, CPX-1 and related nano-platinum / nano-doxorubicin conjugates	Various solid tumors	Phase II/III – mostly negative or equivocal	Modest response rates and limited survival gains in small phase II studies; no subsequent positive Phase III data to support registration	Legacy cytotoxic repackaging in an era increasingly dominated by IO and targeted therapies; lack of biomarker-guided enrichment; payer skepticism about “me-too” nano-chemotherapy	[195–198]
MM-302 / ^{64}Cu-MM-302 (HER2-targeted liposomal doxorubicin)	HER2-positive metastatic breast cancer	Phase II – development halted after negative trial	Heterogeneous tumor uptake on ^{64}Cu-PET, diluted efficacy in unselected, heavily pretreated population; no survival advantage over standard HER2-targeted regimens	Overreliance on EPR/targeted delivery without imaging-based enrichment; trialing in late-line, heterogeneous cohorts; competition from highly effective HER2-directed antibody and TKI combinations	[111–118]
BIND-014 (PSMA-targeted docetaxel NP)	mCRPC, NSCLC and other solid tumors	Phase II – no further late-stage development; company bankruptcy	Variable tumor accumulation and no consistent superiority in response or PFS vs docetaxel; program terminated amid corporate collapse	Limits of “active targeting” when target expression and vascular access are heterogeneous; crowded indications; vulnerability of complex nano-platforms to	[124–127]

Product / platform	Primary indication(s) targeted	Phase where stalled or constrained	Proximate reason for failure / limited success	Underlying systemic barrier(s) illustrated	References
				financial and commercial shocks	
CRLX101 (cyclodextrin–camptothecin nanopharmaceutical)	RCC, NSCLC, rectal cancer and others	Multiple Phase II trials; no successful Phase III registration	Modest response and survival gains; toxicity (myelosuppression, GI) not dramatically better than SOC; inconclusive add-on benefit to chemoradiation	Testing in unselected populations with aggressive backbones; rapidly shifting standards of care towards IO + targeted combinations; failure to deliver a clear therapeutic index advantage	[120–123]
ThermoDox (lyso-thermosensitive liposomal doxorubicin)	HCC (with RFA), early-stage breast cancer (with hyperthermia)	Phase III (HEAT, OPTIMA and others) – largely negative	Negative primary endpoints attributed to inconsistent thermal dosing, heterogeneous heat coverage and suboptimal timing of drug release; benefit only in post-hoc subgroups	Operational complexity of drug–device combinations; difficulty standardizing locoregional hyperthermia in real-world practice; absence of prospective biomarker/parameter stratification	[82–86]
DepoCyt (liposomal cytarabine for intrathecal use)	Lymphomatous meningitis	Approved but later withdrawn in some regions	High rates of chemical arachnoiditis and neurotoxicity despite improved CSF exposure; burdensome intrathecal administration and monitoring requirements	Narrow therapeutic window; logistical complexity limiting real-world adoption; emerging alternative systemic and intrathecal regimens eroding perceived added value	[14–21]
MRX34 (liposomal miR-34a mimic)	Advanced solid tumors and	Phase I – program terminated early	Severe immune-mediated adverse events, including cytokine-mediated	Incomplete understanding of RNA–NP immunobiology;	[185–188]

Product / platform	Primary indication(s) targeted	Phase where stalled or constrained	Proximate reason for failure / limited success	Underlying systemic barrier(s) illustrated	References
	hematologic malignancies		reactions and fatalities; no safe dose achieving robust miR-34a modulation	nano-platform introducing new systemic toxicities; heightened regulatory caution for miRNA nanotherapeutics after early safety signals	
DCR-MYC (lipid NP siRNA targeting MYC)	Advanced solid tumors and lymphoma	Phase I – early termination	Insufficient MYC knockdown and anti-tumor activity at tolerable doses; infusion-related reactions typical of some lipid NPs	Challenges achieving deep intracellular target engagement in solid tumors; platform-specific immunogenicity; emergence of alternative MYC-directed approaches (small molecules, PROTACs)	[176–179]
CALAA-01 (cyclodextrin-based, transferrin-targeted siRNA NP)	Advanced solid tumors	Phase I – development discontinued	Proof-of-concept for targeted siRNA delivery with acceptable safety but limited tumor responses; highly complex architecture and demanding CMC profile	Non-scalable, expensive manufacturing; sensitivity of PK/PD to subtle process changes; rapid obsolescence once simpler, scalable LNP platforms became available	[104–106]
ExoASO-STAT6, iExosomes and related exosome-based platforms	Solid tumors (e.g. liver, PDAC)	Early-phase or preclinical; no advanced clinical read-outs	Slow recruitment, CMC and batch-release hurdles; difficulty demonstrating consistent product quality and potency	Extreme challenges in scalable, reproducible exosome production; heterogeneous vesicle populations; unclear regulatory frameworks for definition and	[157–159]

Product / platform	Primary indication(s) targeted	Phase where stalled or constrained	Proximate reason for failure / limited success	Underlying systemic barrier(s) illustrated	References
				control of “exosome drugs”	
NanoTherm / MagForce SPION hyperthermia	Glioblastoma, prostate cancer	CE-marked but minimal uptake; company later insolvent	Complex procedure requiring stereotactic NP injection plus dedicated magnetic field equipment; small, mixed clinical datasets without definitive Phase III evidence; regulatory and operational issues	Site- and operator-dependence of sophisticated nano-device procedures; difficulty convincing payers and clinicians with limited, non-randomized data; financial fragility of device-drug companies	[131–134]
Liposomal anthracyclines and taxanes as a class (DaunoXome, Lipusu, Abraxane, Nanoxel, Genexol-PM, etc.)	Breast, lung, gastric, ovarian and other solid tumors	Multiple Phase II/III trials in various lines and combinations	Frequently failed to demonstrate OS/PFS superiority versus solvent-based comparators, even when tolerability improved; benefits often restricted to specific subgroups	Health-system insistence on cost-effectiveness; incremental chemotherapy optimization overshadowed by IO, ADCs and targeted agents; lack of biomarker-guided positioning and clear niche definition	[7–13],[28–32], [33–36], [37–41], [45–49]

Supplementary Table 3. Artificial intelligence (AI) Models for Nanomedicine in Cancer Theranostics

AI Model	Possible/Current Use Case	Advantages	Limitations	Data Requirements	Resource Requirements	XAI Level	References
CNNs (convolutional neural networks)	SEM/histopathology image classification	High accuracy in image tasks; spatial feature extraction	Requires large, labeled datasets; black-box	Annotated imaging (SEM, MRI, pathology)	High (GPU/TPU)	Low–Medium (Grad-CAM, saliency maps)	[286–289]
RNNs / LSTM	Time-series NP behavior, drug kinetics	Captures temporal dependencies	Vanishing gradients; not ideal	Longitudinal biosensor/drug data	Moderate	Low	[290–292]

			for long sequences				
Transformers (BERT, T5, GPT)	Inverse synthesis, biomedical text mining	Handles long dependencies; strong NLP modeling	Requires huge datasets; expensive	Large text/structured synthesis corpora	Very high (TPU or A100)	Low (attention maps only)	[293–296]
Random forests	Drug response, efficacy prediction	Easy to train; interpretable; robust	Less suitable for imaging or time-series	Tabular clinical/biological data	Low-moderate	High (feature importance, SHAP)	[297–300]
SVMs (support vector machines)	Binary classification (toxicity, response)	Works on small high-dimensional data	Kernel choice sensitive; slow on large datasets	Mid-sized feature datasets	Low-moderate	Medium	[301–304]
GNNs (graph neural networks)	Molecular interaction networks, ligand design	Captures graph structure; ideal for molecule modeling	Complex to implement and interpret	Graph-structured molecule/interaction data	High	Medium	[305–308]
VAEs (variational autoencoders)	Latent embedding of morphology; synthetic design	Learns unsupervised representations	Blurry image outputs; hard to control	Unlabeled NP SEM data	Moderate-high	Low	[309–313]
GANs (generative adversarial networks)	SEM image synthesis, augmentation	High-quality synthetic data generation	Training instability; non-interpretable	High-quality training images	High (GPU required)	Very low	[314–316]
BNNs (Bayesian neural networks)	Uncertainty-aware prediction (e.g. toxicity)	Quantifies uncertainty; safety-critical applications	Slow; complex posterior estimation	Any supervised data with uncertainty needs	High	High	[317–320]
Reinforcement learning (RL)	Optimizing NP synthesis/formulation	Learns from trial-and-error; suitable for design space exploration	Data-hungry; hard to tune reward	Simulated or real reward-based systems	High	Low (policy trace only)	[321–323]
k-NN (k-nearest neighbor)	Basic NP classification	Simple; intuitive; no training time	Poor scalability	Small tabular/imag	Low	High	[324–327]

			; sensitive to noise	e embeddings			
Decision tree	Initial clinical rules for nano-bio interactions	Highly interpretable ; fast	Prone to overfittin g; limited generaliza tion	Tabular, categorical clinical/biol ogical data	Very low	Very high	[328–330]
Logistic regression	Baseline binary tasks (toxicity, targeting)	Interpretable coefficients	Limited expressiv eness for complex tasks	Small tabular datasets	Very low	Very high	[331–333]
AutoML (TPOT, AutoKeras, H2O)	Rapid model development for nano-bio pipelines	Speeds up pipeline optimization ; low barrier	Black-box automatio n; may miss domain logic	Structured biological datasets	Moderate -high	Medium (depends on selected models)	[334–336]
Multi-task learning (MTL)	Simultaneou s prediction of toxicity, size, targeting	Leverages shared knowledge across tasks	Requires careful task weighting	Multi-label NP datasets	High	Medium	[337–340]
Ensemble methods (stacked models, voting)	Combining multiple models for drug efficacy/toxi city	Improves accuracy and robustness	Computat ionally intensive; interpreta bility reduced	Diverse structured datasets	Moderate –high	Medium –high	[341–344]
XGBoost/LightGB M/CatBoost	Drug synergy, MIC prediction, patient stratification	Fast training, interpretable SHAP values	May overfit small/nois y data	Clean tabular features from experiments	Moderate	High (native SHAP support)	[345–347]
Self-supervised learning (e.g., SimCLR, SimCLIC)	Learning NP image features without labels	Requires no annotation; boosts downstream tasks	Needs large batch sizes and augmenta tions	Raw SEM images or sensor signals	High	Low–medium	[348–351]
Few-shot/meta-learning models	Low-sample regime (rare particle types, rare drugs)	Generalizes from few examples	Training instability ; requires tailored architectu res	Few annotated examples, synthetic data	High	Medium	[352–355]
Deep belief networks (DBNs)	Drug release pattern modeling	Layer-wise pretraining	Outdated vs. modern	Numerical time-series or	Moderate	Low	[356–358]

		helps convergence	DNNs; hard to tune	physicochemical data			
Capsule networks	Morphology-aware NP classification	Retains spatial hierarchies; better equivariance	Hard to train; limited adoption	Annotated NP images	High	Medium	[359–362]
Neural ODEs (ordinary differential equations)	Modeling NP diffusion and drug kinetics	Models continuous dynamics; interpretable mathematically	Difficult to scale; unstable gradients	Continuous system data (e.g., drug diffusion profiles)	High	Medium	[363–365]
Physics-informed neural networks (PINNs)	Modeling NP transport in TME	Integrates domain physics into training	Complex PDE loss terms; hard to optimize	Biophysical models + experimental validation data	High	Medium–high	[366–369]
Federated learning (FL)	Multi-institutional nanomedicine prediction models	Data privacy preserved across hospitals	Communication overhead; heterogeneous models	Distributed clinical/NP datasets	Very high (across devices/servers)	Medium	[370–373]
Neural radiance fields (NeRFs)	3D reconstruction of NP surfaces or tumors	Generates high-fidelity 3D structures	Very slow to train/infer; early-stage	Multiview SEM/CT image data	Very high	Low	[374–377]
Contrastive learning (SimCLR, MoCo)	Self-supervised NP representation learning	No annotation required; downstream performance boost	Needs heavy augmentation; not very interpretable	Raw images or spectrometry signals	High	Medium	[378,379]
Diffusion models (DDPMs)	Generative modeling of NP morphology or drug design	High-resolution generation; controllable noise	Long training; interpretability unclear	Large high-quality NP images or molecules	Very high	Low	[380–383]
Equivariant neural networks (e.g., SE(3)-Transformers)	Molecular interaction modeling respecting symmetries	Captures 3D rotations/translations correctly	Limited toolkits; emerging field	Molecular 3D structures	High	Medium	[384–386]
Attention-based graph models	NP-cell interaction graphs	Learn important nodes/edges	Sensitive to topology;	Graphs of biological interactions	High	Medium–high	[387–390]

(GAT, Graphormer)		with attention	harder to debug				
Sparse Bayesian models (e.g., relevance vector machines)	Interpretable prediction of response with uncertainty	Compact model; probabilistic outputs	Slow training; not scalable	Small to medium tabular data	Moderate	High	[391–393]
HyperNetworks	Generates task-specific networks for different NP targets	Task conditioning for flexibility	Hard to debug; black-box	Paired NP-task data	High	Low	[394–397]
Zero-shot and few-shot transformers (e.g., T5, FLAN-UL2)	Morphology prediction or synthesis from prompt	No fine-tuning needed; flexible	Prompt sensitivity; hallucination risk	Prompt-response pairs or synthetic description datasets	Very high	Medium (via prompt interpretability)	[398,399]
Multi-modal transformers (e.g., CLIP, GIT, Flamingo)	Joint modeling of SEM images + textual synthesis protocols	Learn from vision-language pairs	Needs aligned multimodal datasets	Image-text pairs (e.g., SEM + synthesis)	Very high	Medium	[400–403]
Graph diffusion models	Molecular generation with controlled topologies	Better validity than GANs for molecules	Less mature toolkits; requires graphs	Molecular graphs (SMILES, PDB, etc.)	High	Medium	[404–407]
BioBERT / SciBERT	Literature mining for nanomedicine discovery	Domain-specific knowledge of biomedical corpora	Static embeddings; pretraining limits	PubMed, Scopus, Elsevier articles	Medium	Medium	[408–412]
SHAP/LIME/Integrated gradients (post-hoc explainability)	Explains black-box nanomedicine models	Intuitive explanations; model-agnostic	May mislead for deep models	Any ML model output	Low (adds interpretability to others)	Very high	[413–416]

CNN, convolutional neural network; SEM, scanning electron microscopy; MRI, magnetic resonance imaging; RNN, recurrent neural network; LSTM, long short-term memory; BERT, bidirectional encoder representations from transformers; T5, text-to-text transfer transformer; GPT, generative pre-trained transformer; NLP, natural language processing; TPU, tensor processing unit; GPU, graphics processing unit; SHAP, SHapley additive explanations; SVM, support vector machine; GNN, graph neural network; VAE, variational autoencoder; GAN, generative adversarial network; BNN, Bayesian neural network; RL, reinforcement learning; k-NN, k-nearest neighbor; DBN, deep belief network; ODE, ordinary differential equation; PINN, physics-informed neural network; PDE, partial differential equation; FL, federated learning;

NeRF, neural radiance field; SimCLR, simple framework for contrastive learning of visual representations; MoCo, momentum contrast; DDPM, denoising diffusion probabilistic model; SE(3), special Euclidean group in 3D; GAT, graph attention network; RVM, relevance vector machine; CLIP, contrastive language–image pretraining; GIT, generative image-to-text; SMILES, simplified molecular-input line-entry system; PDB, protein data bank; BioBERT, biomedical BERT; SciBERT, scientific BERT; LIME, local interpretable model-agnostic explanations.

References:

1. Gabizon AA, Gabizon-Peretz S, Modaresahmadi S, La-Beck NM. Thirty years from FDA approval of pegylated liposomal doxorubicin (Doxil/Caelyx): an updated analysis and future perspective. BMJ Oncol [Internet]. 2025 [cited 2025 July 12];4:e000573. https://doi.org/10.1136/bmjonc-2024-000573
2. Li Y, Qi L, Wang Y, Li Y, Lei C, Zhang Y, et al. A multicenter randomized trials to compare the bioequivalence and safety of a generic doxorubicin hydrochloride liposome injection with Caelyx ® in advanced breast cancer. Front Oncol [Internet]. 2022 [cited 2025 July 12];12:1070001. https://doi.org/10.3389/fonc.2022.1070001
3. O'Brien MER, Wigler N, Inbar M, Rosso R, Grischke E, Santoro A, et al. Reduced cardiotoxicity and comparable efficacy in a phase IIItrial of pegylated liposomal doxorubicin HCl(CAELYX™/Doxil®) versus conventional doxorubicin forfirst-line treatment of metastatic breast cancer. Annals of Oncology [Internet]. 2004 [cited 2025 July 12];15:440–9. https://doi.org/10.1093/annonc/mdh097
4. Harrington KJ, Lewanski C, Northcote AD, Whittaker J, Peters AM, Vile RG, et al. Phase II study of pegylated liposomal doxorubicin (Caelyx™) as induction chemotherapy for patients with squamous cell cancer of the head and neck. European Journal of Cancer [Internet]. Elsevier; 2001 [cited 2025 July 12];37:2015–22. https://doi.org/10.1016/S0959-8049(01)00216-7
5. Judson I, Radford JA, Harris M, Blay J-Y, Hoesel Q van, Cesne A le, et al. Randomised phase II trial of pegylated liposomal doxorubicin (DOXIL®/CAELYX®) versus doxorubicin in the treatment of advanced or metastatic soft tissue sarcoma: a study by the EORTC Soft Tissue and Bone Sarcoma Group. European Journal of Cancer [Internet]. Elsevier; 2001 [cited 2025 July 12];37:870–7. https://doi.org/10.1016/S0959-8049(01)00050-8
6. Gibbs DD, Pyle L, Allen M, Vaughan M, Webb A, Johnston SRD, et al. A phase I dose-finding study of a combination of pegylated liposomal doxorubicin (Doxil), carboplatin and paclitaxel in ovarian cancer. Br J Cancer [Internet]. Nature Publishing Group; 2002 [cited 2025 July 12];86:1379–84. https://doi.org/10.1038/sj.bjc.6600250
7. Petre CE, Dittmer DP. Liposomal daunorubicin as treatment for Kaposi's sarcoma. Int J Nanomedicine [Internet]. 2007 [cited 2025 Aug 1];2:277–88. https://www.ncbi.nlm.nih.gov/pmc/articles/PMC2676651/. Accessed 1 Aug 2025
8. Mohrbacher AF, Gregory SA, Gabriel DA, Rusk JM, Giles FJ. Liposomal daunorubicin (DaunoXome) plus dexamethasone for patients with multiple myeloma. Cancer [Internet]. 2002 [cited 2025 Aug 1];94:2645–52. https://doi.org/10.1002/cncr.10561
9. Money-Kyrle JF, Bates F, Ready J, Gazzard BG, Phillips RH, Boag FC. Liposomal daunorubicin in advanced Kaposi's sarcoma: a phase II study. Clin Oncol (R Coll Radiol). 1993;5:367–71. https://doi.org/10.1016/s0936-6555(05)80088-3
10. Flinn IW, Goodman SN, Post L, Jamison J, Miller CB, Gore S, et al. A dose-finding study of liposomal daunorubicin with CVP (COP-X) in advanced NHL. Ann Oncol. 2000;11:691–5. https://doi.org/10.1023/a:1008361914894
11. Fassas A, Anagnostopoulos A. The use of liposomal daunorubicin (DaunoXome) in acute myeloid leukemia. Leukemia & Lymphoma [Internet]. Taylor & Francis; 2005 [cited 2025 Aug 19]; https://doi.org/10.1080/10428190500052438
12. Mukwaya G, Forssen EA, Schmidt P, Ross M. DaunoXome® (Liposomal Daunorubicin) for First-Line Treatment of Advanced, HIV-Related Kaposi's Sarcoma. In: Woodle MC, Storm G, editors. Long Circulating Liposomes: Old Drugs, New Therapeutics [Internet]. Berlin, Heidelberg:

Springer Berlin Heidelberg; 1998 [cited 2025 Aug 19]. p. 147–63. https://doi.org/10.1007/978-3-662-22115-0_10
13. Rosenthal E, Poizot-Martin I, Saint-Marc T, Spano J-P, Cacoub P, Group the DS. Phase IV Study of Liposomal Daunorubicin (DaunoXome) in AIDS-Related Kaposi Sarcoma. American Journal of Clinical Oncology. 2002;25:57.
14. Salehi B, Selamoglu Z, S. Mileski K, Pezzani R, Redaelli M, C. Cho W, et al. Liposomal Cytarabine as Cancer Therapy: From Chemistry to Medicine. Biomolecules [Internet]. 2019 [cited 2025 Aug 1];9:773. https://doi.org/10.3390/biom9120773
15. Arshad N, Biswas N, Gill J, Kesari S, Ashili S. Drug delivery in leptomeningeal disease: Navigating barriers and beyond. Drug Delivery [Internet]. Taylor & Francis; 2024 [cited 2025 Aug 1];31:2375521. https://doi.org/10.1080/10717544.2024.2375521
16. Barbour AB, Kotecha R, Lazarev S, Palmer JD, Robinson T, Yerramilli D, et al. Radiation Therapy in the Management of Leptomeningeal Disease From Solid Tumors. Advances in Radiation Oncology [Internet]. Elsevier; 2024 [cited 2025 Aug 1];9. https://doi.org/10.1016/j.adro.2023.101377
17. Wilcox JA, Li MJ, Boire AA. Leptomeningeal Metastases: New Opportunities in the Modern Era. Neurotherapeutics [Internet]. 2022 [cited 2025 Aug 1];19:1782–98. https://doi.org/10.1007/s13311-022-01261-4
18. Glantz MJ, Jaeckle KA, Chamberlain MC, Phuphanich S, Recht L, Swinnen LJ, et al. A randomized controlled trial comparing intrathecal sustained-release cytarabine (DepoCyt) to intrathecal methotrexate in patients with neoplastic meningitis from solid tumors. Clin Cancer Res. 1999;5:3394–402.
19. Demopoulos A. Leptomeningeal metastases. Curr Neurol Neurosci Rep. 2004;4:196–204. https://doi.org/10.1007/s11910-004-0039-z
20. DeAngelis LM, Boutros D. Leptomeningeal Metastasis. Cancer Investigation. Taylor & Francis; 2005;23:145–54. https://doi.org/10.1081/CNV-50458
21. Camera A, Cerciello G, Perna F, Rinaldi CR, Michele E, Ferrari S, et al. Liposomal Cytarabine (Depocyte®) for the Treatment of Meningeal or CNS Disease in Acute Leukemias (AL) and Non-Hodgkin Lymphomas (NHL): A Single Centre Experience. Blood. 2006;108:4540. https://doi.org/10.1182/blood.V108.11.4540.4540
22. Kemp JA, Kwon YJ. Cancer nanotechnology: current status and perspectives. Nano Convergence [Internet]. 2021 [cited 2025 Aug 1];8:34. https://doi.org/10.1186/s40580-021-00282-7
23. Leonard RCF, Williams S, Tulpule A, Levine AM, Oliveros S. Improving the therapeutic index of anthracycline chemotherapy: Focus on liposomal doxorubicin (Myocet™). The Breast. 2009;18:218–24. https://doi.org/10.1016/j.breast.2009.05.004
24. Batist G, Barton J, Chaikin P, Swenson C, Welles L. Myocet (liposome-encapsulated doxorubicin citrate): a new approach in breast cancer therapy. Expert Opinion on Pharmacotherapy. Taylor & Francis; 2002;3:1739–51. https://doi.org/10.1517/14656566.3.12.1739
25. Akter H. Comparison of conventional doxorubicin with liposome encapsulated doxorubicin as a treatment of breast cancer-a review [Internet] [Thesis]. Brac University; 2023 [cited 2025 Aug 19]. https://dspace.bracu.ac.bd:8443/xmlui/handle/10361/23619. Accessed 19 Aug 2025
26. Buonadonna A, Scalone S, Lombardi D, Fumagalli A, Guglielmi A, Lestuzzi C, et al. Prospective, Multicenter Phase II Trial of Non-Pegylated Liposomal Doxorubicin Combined with Ifosfamide in First-Line Treatment of Advanced/Metastatic Soft Tissue Sarcomas. Cancers.

Multidisciplinary Digital Publishing Institute; 2023;15:5036. https://doi.org/10.3390/cancers15205036
27. Li Z. Adverse events pro les of liposomal and conventional doxorubicins: An updated comprehensive analysis of the FDA adverse event reporting system.
28. Li L, Zhan Q, Yi K, Chen N, Li X, Yang S, et al. Engineering Lipusu with lysophosphatidylcholine for improved tumor cellular uptake and anticancer efficacy. J Mater Chem B [Internet]. The Royal Society of Chemistry; 2022 [cited 2025 Aug 9];10:1833–42. https://doi.org/10.1039/D1TB02823E
29. Koudelka Š, Turánek J. Liposomal paclitaxel formulations. Journal of Controlled Release. 2012;163:322–34. https://doi.org/10.1016/j.jconrel.2012.09.006
30. Xu X, Wang L, Xu H-Q, Huang X-E, Qian Y-D, Xiang J. Clinical Comparison between Paclitaxel Liposome (Lipusu®) and Paclitaxel for Treatment of Patients with Metastatic Gastric Cancer. Asian Pacific Journal of Cancer Prevention. 2013;14:2591–4. https://doi.org/10.7314/APJCP.2013.14.4.2591
31. Kumar S, Arora A, Pant V, Guchhait S, Kumar R, Mathur D, et al. Advances in Drug Delivery Systems for Lipophilic Drug Paclitaxel: Developments, Challenges, and Opportunities (A Review). Russ J Bioorg Chem. 2024;50:1752–82. https://doi.org/10.1134/S106816202405011X
32. Muley A, Navale A, Gupta R, Tekade RK. “Nano-Paclitaxel” Unlocking Potential and Redefining Cancer Chemotherapy. ACS Omega. 2025;10:28670–90. https://doi.org/10.1021/acsomega.5c02916
33. Svenson S. Clinical translation of nanomedicines. Current Opinion in Solid State and Materials Science. 2012;16:287–94. https://doi.org/10.1016/j.cossms.2012.10.001
34. Tian Z, Yao W. Albumin-Bound Paclitaxel: Worthy of Further Study in Sarcomas. Front Oncol [Internet]. Frontiers; 2022 [cited 2025 Aug 19];12. https://doi.org/10.3389/fonc.2022.815900
35. Yared JA, Tkaczuk KH. Update on taxane development: new analogs and new formulations. Drug Design, Development and Therapy. Dove Medical Press; 2012;6:371–84. https://doi.org/10.2147/DDDT.S28997
36. Parodi A, Kolesova EP, Voronina MV, Frolova AS, Kostyushev D, Trushina DB, et al. Anticancer Nanotherapeutics in Clinical Trials: The Work behind Clinical Translation of Nanomedicine. International Journal of Molecular Sciences. Multidisciplinary Digital Publishing Institute; 2022;23:13368. https://doi.org/10.3390/ijms232113368
37. Serras A, Faustino C, Pinheiro L. Functionalized Polymeric Micelles for Targeted Cancer Therapy: Steps from Conceptualization to Clinical Trials. Pharmaceutics. Multidisciplinary Digital Publishing Institute; 2024;16:1047. https://doi.org/10.3390/pharmaceutics16081047
38. Mahajan M, Khurana RK, Sahajpal NS, Utreja P, Sankar R, Singh B, et al. Emerging Strategies and Challenges for Controlled Delivery of Taxanes: A Comprehensive Review. Current Drug Metabolism. Bentham Science Publishers; 2015;16:453–73. https://doi.org/10.2174/1389200216666150812123414
39. Dhanabalan D, Shanmugam N. Nanomedicine in cancer treatment - an overview. Adv Nat Sci: Nanosci Nanotechnol. IOP Publishing; 2024;16:013001. https://doi.org/10.1088/2043-6262/ad9f49
40. Gwak G, Chung MS, Kim TH, Park I, Kim J, Um EH, et al. A Multi-Center Trial to Evaluate the Safety and Toxicity of Nanoxel®-M in Breast Cancer Patients. Journal of Breast Disease. 한국유방암학회; 2021;9:45–55. https://doi.org/10.14449/jbd.2021.9.2.45
41. Martín-Sabroso C, Fraguas-Sánchez AI, Raposo-González R, Torres-Suárez AI. Perspectives in Breast and Ovarian Cancer Chemotherapy by Nanomedicine Approach: Nanoformulations in

Clinical Research. Current Medicinal Chemistry. 2021;28:3271–86. https://doi.org/10.2174/0929867327666200819115403
42. Heo Y-A, Syed YY, Keam SJ. Pegaspargase: A Review in Acute Lymphoblastic Leukaemia. Drugs. 2019;79:767–77. https://doi.org/10.1007/s40265-019-01120-1
43. Radadiya A, Zhu W, Coricello A, Alcaro S, Richards NGJ. Improving the Treatment of Acute Lymphoblastic Leukemia. Biochemistry. American Chemical Society; 2020;59:3193–200. https://doi.org/10.1021/acs.biochem.0c00354
44. Tosta Pérez M, Herrera Belén L, Letelier P, Calle Y, Pessoa A, Farías JG. l-Asparaginase as the gold standard in the treatment of acute lymphoblastic leukemia: a comprehensive review. Med Oncol. 2023;40:150. https://doi.org/10.1007/s12032-023-02014-9
45. Keam B, Lee K-W, Lee S-H, Kim J-S, Kim JH, Wu H-G, et al. A Phase II Study of Genexol-PM and Cisplatin as Induction Chemotherapy in Locally Advanced Head and Neck Squamous Cell Carcinoma. The Oncologist. 2019;24:751-e231. https://doi.org/10.1634/theoncologist.2019-0070
46. Serras A, Faustino C, Pinheiro L. Functionalized Polymeric Micelles for Targeted Cancer Therapy: Steps from Conceptualization to Clinical Trials. Pharmaceutics. Multidisciplinary Digital Publishing Institute; 2024;16:1047. https://doi.org/10.3390/pharmaceutics16081047
47. Kim D-W, Kim S-Y, Kim H-K, Kim S-W, Shin SW, Kim JS, et al. Multicenter phase II trial of Genexol-PM, a novel Cremophor-free, polymeric micelle formulation of paclitaxel, with cisplatin in patients with advanced non-small-cell lung cancer. Annals of Oncology. 2007;18:2009–14. https://doi.org/10.1093/annonc/mdm374
48. Li H, Fang Y, Li X, Tu L, Xu G, Jin Y, et al. Evaluation of novel paclitaxel-loaded NO-donating polymeric micelles for an improved therapy for gastroenteric tumor. New J Chem. The Royal Society of Chemistry; 2021;45:13763–74. https://doi.org/10.1039/D1NJ00979F
49. Werner ME, Cummings ND, Sethi M, Wang EC, Sukumar R, Moore DT, et al. Preclinical Evaluation of Genexol-PM, a Nanoparticle Formulation of Paclitaxel, as a Novel Radiosensitizer for the Treatment of Non-Small Cell Lung Cancer. International Journal of Radiation Oncology*Biology*Physics. 2013;86:463–8. https://doi.org/10.1016/j.ijrobp.2013.02.009
50. Biteau K, Guiho R, Chatelais M, Taurelle J, Chesneau J, Corradini N, et al. L-MTP-PE and zoledronic acid combination in osteosarcoma: preclinical evidence of positive therapeutic combination for clinical transfer. Am J Cancer Res. 2016;6:677–89.
51. Hattinger CM, Patrizio MP, Magagnoli F, Luppi S, Serra M. An update on emerging drugs in osteosarcoma: towards tailored therapies? Expert Opinion on Emerging Drugs. Taylor & Francis; 2019;24:153–71. https://doi.org/10.1080/14728214.2019.1654455
52. Kaniklidis C. Osteosarcoma: Emerging Therapies.
53. Mahmoudi K, Bouras A, Bozec D, Ivkov R, Hadjipanayis C. Magnetic hyperthermia therapy for the treatment of glioblastoma: a review of the therapy's history, efficacy and application in humans. International Journal of Hyperthermia. Taylor & Francis; 2018;34:1316–28. https://doi.org/10.1080/02656736.2018.1430867
54. Maheshwari S, Singh A, Verma A, Shariq M, Akhtar J, Alsaidan OA, et al. Superparamagnetic iron oxide nanoparticles (SPIONs) in targeting brain tumors: advances and challenges. Med Oncol. 2025;42:338. https://doi.org/10.1007/s12032-025-02913-z
55. Verma J, Lal S, Van Noorden CJ. Nanoparticles for hyperthermic therapy: synthesis strategies and applications in glioblastoma. International Journal of Nanomedicine. Dove Medical Press; 2014;9:2863–77. https://doi.org/10.2147/IJN.S57501
56. Manescu (Paltanea) V, Antoniac I, Paltanea G, Nemoianu IV, Mohan AG, Antoniac A, et al. Magnetic Hyperthermia in Glioblastoma Multiforme Treatment. International Journal of

Molecular Sciences. Multidisciplinary Digital Publishing Institute; 2024;25:10065. https://doi.org/10.3390/ijms251810065
57. Douer D. Efficacy and Safety of Vincristine Sulfate Liposome Injection in the Treatment of Adult Acute Lymphocytic Leukemia. Oncologist. 2016;21:840–7. https://doi.org/10.1634/theoncologist.2015-0391
58. Pathak P, Hess R, Weiss MA. Liposomal vincristine for relapsed or refractory Ph-negative acute lymphoblastic leukemia: a review of literature. Therapeutic Advances in Hematology. SAGE Publications; 2014;5:18–24. https://doi.org/10.1177/2040620713519016
59. Said R, Tsimberidou AM. Pharmacokinetic evaluation of vincristine for the treatment of lymphoid malignancies. Expert Opinion on Drug Metabolism & Toxicology. Taylor & Francis; 2014;10:483–94. https://doi.org/10.1517/17425255.2014.885016
60. Seegars MB, Woods R, Ellis LR, Bhave RR, Howard DS, Manuel M, et al. A Pilot Phase II Study of the Feasibility and Efficacy of Vincristine Sulfate Liposome Injection in Patients With Relapsed or Refractory Acute Myeloid Leukemia. J Hematol. 2021;10:1–7. https://doi.org/10.14740/jh771
61. Terwilliger T, Abdul-Hay M. Acute lymphoblastic leukemia: a comprehensive review and 2017 update. Blood Cancer J. Nature Publishing Group; 2017;7:e577–e577. https://doi.org/10.1038/bcj.2017.53
62. Lamb YN, Scott LJ. Liposomal Irinotecan: A Review in Metastatic Pancreatic Adenocarcinoma. Drugs. 2017;77:785–92. https://doi.org/10.1007/s40265-017-0741-1
63. Melisi D, Casalino S, Pietrobono S, Quinzii A, Zecchetto C, Merz V. Integration of liposomal irinotecan in the first-line treatment of metastatic pancreatic cancer: try to do not think about the white bear. Ther Adv Med Oncol. SAGE Publications Ltd STM; 2024;16:17588359241234487. https://doi.org/10.1177/17588359241234487
64. Bernards N, Ventura M, Fricke IB, Hendriks BS, Fitzgerald J, Lee H, et al. Liposomal Irinotecan Achieves Significant Survival and Tumor Burden Control in a Triple Negative Breast Cancer Model of Spontaneous Metastasis. Mol Pharmaceutics. American Chemical Society; 2018;15:4132–8. https://doi.org/10.1021/acs.molpharmaceut.8b00540
65. Grapsa D, Syrigos K, Saif MW. Nanoliposomal irinotecan for treating pancreatic cancer. Expert Opinion on Orphan Drugs. Taylor & Francis; 2016;4:541–7. https://doi.org/10.1517/21678707.2016.1169171
66. Chaudhuri A, Kumar DN, Shaik RA, Eid BG, Abdel-Naim AB, Md S, et al. Lipid-Based Nanoparticles as a Pivotal Delivery Approach in Triple Negative Breast Cancer (TNBC) Therapy. International Journal of Molecular Sciences. Multidisciplinary Digital Publishing Institute; 2022;23:10068. https://doi.org/10.3390/ijms231710068
67. Wang J, Li X, Wu W, Xu X-M, Xu H, Zhang T. Recent Progress of Paclitaxel Delivery Systems: Covalent and Noncovalent Approaches. Advanced Therapeutics. 2023;6:2200281. https://doi.org/10.1002/adtp.202200281
68. Jha P, Anjum V, Choudhary R, Kadi A, Ali F, Potoroko I. Nanosomal-mediated Lipid Suspension Delivery of Docetaxel as a PromisingApproach to Enhance Therapeutic Potential in Triple-Negative Breast Cancer. ACAMC [Internet]. 2025 [cited 2025 Aug 21];25. https://doi.org/10.2174/0118715206366378250519105734
69. Tzogani K, Penttilä K, Lapveteläinen T, Hemmings R, Koenig J, Freire J, et al. EMA Review of Daunorubicin and Cytarabine Encapsulated in Liposomes (Vyxeos, CPX-351) for the Treatment of Adults with Newly Diagnosed, Therapy-Related Acute Myeloid Leukemia or Acute Myeloid

Leukemia with Myelodysplasia-Related Changes. Oncologist. 2020;25:e1414–20. https://doi.org/10.1634/theoncologist.2019-0785
70. Blair HA. Daunorubicin/Cytarabine Liposome: A Review in Acute Myeloid Leukaemia. Drugs. 2018;78:1903–10. https://doi.org/10.1007/s40265-018-1022-3
71. Kim M, Williams S. Daunorubicin and Cytarabine Liposome in Newly Diagnosed Therapy-Related Acute Myeloid Leukemia (AML) or AML With Myelodysplasia-Related Changes. Ann Pharmacother. SAGE Publications Inc; 2018;52:792–800. https://doi.org/10.1177/1060028018764923
72. Chen EC, Fathi AT, Brunner AM. Reformulating acute myeloid leukemia: liposomal cytarabine and daunorubicin (CPX-351) as an emerging therapy for secondary AML. OncoTargets and Therapy. Dove Medical Press; 2018;11:3425–34. https://doi.org/10.2147/OTT.S141212
73. Alzahrani AM, Alnuhait MA, Alqahtani T. The Clinical Safety and Efficacy of Cytarabine and Daunorubicin Liposome (CPX-351) in Acute Myeloid Leukemia Patients: A Systematic Review. Cancer Reports. 2025;8:e70199. https://doi.org/10.1002/cnr2.70199
74. Chambial P, Thakur N, Preethi L, Mehta P. Anti-Cancer Nanotherapeutics in Clinical Trials: The Work Behind Clinical Translation of Nanomedicine. Nanoparticles in Cancer Therapy. CRC Press; 2024.
75. Sarkar M, Wang Y, Ekpenyong O, Liang D, Xie H. Pharmacokinetic behaviors of soft nanoparticulate formulations of chemotherapeutics. WIREs Nanomedicine and Nanobiotechnology. 2023;15:e1846. https://doi.org/10.1002/wnan.1846
76. Liu Y, Zhao F, Wang Q, Zhao Q, Hou G, Meng Q. Current Perspectives on Paclitaxel: Focus on Its Production, Delivery and Combination Therapy. Mini Reviews in Medicinal Chemistry. Bentham Science Publishers; 2023;23:1780–96. https://doi.org/10.2174/1389557523666230210145150
77. Ding S, Chen L, Liao J, Huo Q, Wang Q, Tian G, et al. Harnessing Hafnium-Based Nanomaterials for Cancer Diagnosis and Therapy. Small. 2023;19:2300341. https://doi.org/10.1002/smll.202300341
78. Hoffmann C, Shen C, Le Tourneau C. Nanoparticle therapy for head and neck cancers. Current Opinion in Oncology. 2022;34:177. https://doi.org/10.1097/CCO.0000000000000828
79. Bilynsky C, Millot N, Papa A-L. Radiation nanosensitizers in cancer therapy—From preclinical discoveries to the outcomes of early clinical trials. Bioengineering & Translational Medicine. 2022;7:e10256. https://doi.org/10.1002/btm2.10256
80. Xiao S, Wang X, Chen B, Mu M, Han B, Chen N, et al. Enhancing tumor radiotherapy sensitivity through metal nanomaterials: A comprehensive review. Malignancy Spectrum. 2024;1:243–62. https://doi.org/10.1002/msp2.52
81. Cao Y, Ding S, Hu Y, Zeng L, Zhou J, Lin L, et al. An Immunocompetent Hafnium Oxide-Based STING Nanoagonist for Cancer Radio-immunotherapy. ACS Nano. American Chemical Society; 2024;18:4189–204. https://doi.org/10.1021/acsnano.3c09293
82. Lyon P, Carlisle R, Coussios CC. Triggered Temperature-Sensitive Liposome Release by Focused Ultrasound for Localised Drug Delivery. Image-guided Focused Ultrasound Therapy. CRC Press; 2024.
83. Yaramiri A, Asalh RA, Asalh MA, AlSawaftah N, Abuwatfa WH, Husseini GA. A Comprehensive Review of Smart Thermosensitive Nanocarriers for Precision Cancer Therapy. International Journal of Molecular Sciences. Multidisciplinary Digital Publishing Institute; 2025;26:7322. https://doi.org/10.3390/ijms26157322

84. Wang Y, Yin Z, Gao L, Ma B, Shi J, Chen H. Lipid Nanoparticles-Based Therapy in Liver Metastasis Management: From Tumor Cell-Directed Strategy to Liver Microenvironment-Directed Strategy. International Journal of Nanomedicine. Dove Medical Press; 2023;18:2939–54. https://doi.org/10.2147/IJN.S402821
85. Wang X, Allen C. Synergistic effects of thermosensitive liposomal doxorubicin, mild hyperthermia, and radiotherapy in breast cancer management: an orthotopic mouse model study. Drug Deliv and Transl Res. 2025;15:1011–22. https://doi.org/10.1007/s13346-024-01654-2
86. Chaudhry M, Lyon P, Coussios C, Carlisle R. Thermosensitive liposomes: a promising step toward localised chemotherapy. Expert Opinion on Drug Delivery. Taylor & Francis; 2022;19:899–912. https://doi.org/10.1080/17425247.2022.2099834
87. Huclier-Markai S, Ntsiba E, Thomas E, Alliot C, Cutler C, Lux F, et al. Multimodal AGuIX® Nanoparticles: Size Characterization by HF5 and Optimization of the Radiolabeling with Various SPECT/PET/Theranostic Tracers. IntJMedNano Res. 2019;6:027. https://doi.org/10.23937/2378-3664.1410027
88. Verry C, Dufort S, Villa J, Gavard M, Iriart C, Grand S, et al. Theranostic AGuIX nanoparticles as radiosensitizer: A phase I, dose-escalation study in patients with multiple brain metastases (NANO-RAD trial). Radiotherapy and Oncology. 2021;160:159–65. https://doi.org/10.1016/j.radonc.2021.04.021
89. Thivat E, Casile M, Moreau J, Molnar I, Dufort S, Seddik K, et al. Phase I/II study testing the combination of AGuIX nanoparticles with radiochemotherapy and concomitant temozolomide in patients with newly diagnosed glioblastoma (NANO-GBM trial protocol). BMC Cancer. 2023;23:344. https://doi.org/10.1186/s12885-023-10829-y
90. Aubrun C, Doussineau T, Carmès L, Meyzaud A, Boux F, Dufort S, et al. Mechanisms of Action of AGuIX as a Pan-Cancer Nano-Radiosensitizer: A Comprehensive Review. Pharmaceuticals. Multidisciplinary Digital Publishing Institute; 2025;18:519. https://doi.org/10.3390/ph18040519
91. Sittplangkoon C. mRNA vaccine encoding neoantigen for cancer immunotherapy using mouse melanoma as a model [Internet] [Doctoral Degree]. [Bangkok, Thailand]: Chulalongkorn University; 2021 [cited 2025 Aug 21]. https://doi.org/10.58837/CHULA.THE.2021.23
92. Sahin U, Oehm P, Derhovanessian E, Jabulowsky RA, Vormehr M, Gold M, et al. An RNA vaccine drives immunity in checkpoint-inhibitor-treated melanoma. Nature. Nature Publishing Group; 2020;585:107–12. https://doi.org/10.1038/s41586-020-2537-9
93. Fu Q, Zhao X, Hu J, Jiao Y, Yan Y, Pan X, et al. mRNA vaccines in the context of cancer treatment: from concept to application. J Transl Med. 2025;23:12. https://doi.org/10.1186/s12967-024-06033-6
94. Chen W, Zhu Y, He J, Sun X. Path towards mRNA delivery for cancer immunotherapy from bench to bedside. Theranostics. 2024;14:96–115. https://doi.org/10.7150/thno.89247
95. Seo H, Jeon L, Kwon J, Lee H. High-Precision Synthesis of RNA-Loaded Lipid Nanoparticles for Biomedical Applications. Advanced Healthcare Materials. 2023;12:2203033. https://doi.org/10.1002/adhm.202203033
96. Kemp JA, Kwon YJ. Cancer nanotechnology: current status and perspectives. Nano Convergence. 2021;8:34. https://doi.org/10.1186/s40580-021-00282-7
97. Huang S, Que H, Wang M, Wei X. mRNA vaccines as cancer therapies. Chinese Medical Journal. Chinese Medical Association Publishing House; 2024;137:2979–95. https://doi.org/10.1097/CM9.0000000000003455

98. Fu Q, Zhao X, Hu J, Jiao Y, Yan Y, Pan X, et al. mRNA vaccines in the context of cancer treatment: from concept to application. J Transl Med. 2025;23:12. https://doi.org/10.1186/s12967-024-06033-6
99. Laila UE, An W, Xu Z-X. Emerging prospects of mRNA cancer vaccines: mechanisms, formulations, and challenges in cancer immunotherapy. Front Immunol [Internet]. Frontiers; 2024 [cited 2025 Aug 21];15. https://doi.org/10.3389/fimmu.2024.1448489
100. Shebbo S, Binothman N, Darwaish M, Niaz HA, Abdulal RH, Borjac J, et al. Redefining the battle against colorectal cancer: a comprehensive review of emerging immunotherapies and their clinical efficacy. Front Immunol [Internet]. Frontiers; 2024 [cited 2025 Aug 21];15. https://doi.org/10.3389/fimmu.2024.1350208
101. Yaremenko AV, Khan MM, Zhen X, Tang Y, Tao W. Clinical advances of mRNA vaccines for cancer immunotherapy. Med [Internet]. Elsevier; 2025 [cited 2025 Aug 21];6. https://doi.org/10.1016/j.medj.2024.11.015
102. Chi W-Y, Hu Y, Huang H-C, Kuo H-H, Lin S-H, Kuo C-TJ, et al. Molecular targets and strategies in the development of nucleic acid cancer vaccines: from shared to personalized antigens. J Biomed Sci. 2024;31:94. https://doi.org/10.1186/s12929-024-01082-x
103. Xu S, Hu Z, Song F, Xu Y, Han X. Lipid nanoparticles: Composition, formulation, and application. Molecular Therapy Methods & Clinical Development [Internet]. Elsevier; 2025 [cited 2025 Aug 21];33. https://doi.org/10.1016/j.omtm.2025.101463
104. Swaminathan G, Shigna A, Kumar A, Byroju VV, Durgempudi VR, Dinesh Kumar L. RNA Interference and Nanotechnology: A Promising Alliance for Next Generation Cancer Therapeutics. Front Nanotechnol [Internet]. Frontiers; 2021 [cited 2025 Aug 21];3. https://doi.org/10.3389/fnano.2021.694838
105. Davis ME. The First Targeted Delivery of siRNA in Humans via a Self-Assembling, Cyclodextrin Polymer-Based Nanoparticle: From Concept to Clinic. Mol Pharmaceutics. American Chemical Society; 2009;6:659–68. https://doi.org/10.1021/mp900015y
106. López-Estévez AM, Lapuhs P, Pineiro-Alonso L, Alonso MJ. Personalized Cancer Nanomedicine: Overcoming Biological Barriers for Intracellular Delivery of Biopharmaceuticals. Advanced Materials. 2024;36:2309355. https://doi.org/10.1002/adma.202309355
107. Hoffmann C, Shen C, Le Tourneau C. Nanoparticle therapy for head and neck cancers. Current Opinion in Oncology. 2022;34:177. https://doi.org/10.1097/CCO.0000000000000828
108. Esa M, Kaewpaiboon S, Srichana T. Fabrication, biodistribution, and toxicological evaluation of mesoporous silica nanoparticles based on preclinical studies intended for cancer therapy: A review. J App Pharm Sci. 2025;15,:001–26. https://doi.org/10.7324/JAPS.2025.220858
109. Li Y, Si Y, Yin H. Nanomaterial-mediated photothermal therapy modulates tumor-associated macrophages: applications in cancer therapy. J Mater Chem B. The Royal Society of Chemistry; 2024;12:11867–86. https://doi.org/10.1039/D4TB01928H
110. Amendoeira A, García LR, Fernandes AR, Baptista PV. Light Irradiation of Gold Nanoparticles Toward Advanced Cancer Therapeutics. Advanced Therapeutics. 2020;3:1900153. https://doi.org/10.1002/adtp.201900153
111. Espelin CW, Leonard SC, Geretti E, Wickham TJ, Hendriks BS. Dual HER2 Targeting with Trastuzumab and Liposomal-Encapsulated Doxorubicin (MM-302) Demonstrates Synergistic Antitumor Activity in Breast and Gastric Cancer. Cancer Res. 2016;76:1517–27. https://doi.org/10.1158/0008-5472.CAN-15-1518
112. Munster P, Krop IE, LoRusso P, Ma C, Siegel BA, Shields AF, et al. Safety and pharmacokinetics of MM-302, a HER2-targeted antibody–liposomal doxorubicin conjugate, in

patients with advanced HER2-positive breast cancer: a phase 1 dose-escalation study. Br J Cancer. Nature Publishing Group; 2018;119:1086–93. https://doi.org/10.1038/s41416-018-0235-2
113. Munster PN, Miller K, Krop IE, Dhindsa N, Reynolds J, Geretti E, et al. A phase I study of MM-302, a HER2-targeted liposomal doxorubicin, in patients with advanced, HER2-positive (HER2+) breast cancer. JCO. Wolters Kluwer; 2012;30:TPS663–TPS663. https://doi.org/10.1200/jco.2012.30.15_suppl.tps663
114. Hepner A, Modi S, Jhaveri K. Targeting HER2/3 in Breast Cancer. Curr Breast Cancer Rep. 2017;9:61–9. https://doi.org/10.1007/s12609-017-0239-6
115. Gaddy DF, Lee H, Zheng J, Jaffray DA, Wickham TJ, Hendriks BS. Whole-body organ-level and kidney micro-dosimetric evaluations of 64Cu-loaded HER2/ErbB2-targeted liposomal doxorubicin (64Cu-MM-302) in rodents and primates. EJNMMI Res. 2015;5:24. https://doi.org/10.1186/s13550-015-0096-0
116. Hendriks B, Shields A, Siegel BA, Miller K, Munster P, Ma C, et al. PET/CT Imaging of 64CU-Labelled HER2 Liposomal Doxorubicin (64CU-MM-302) Quantifies Variability of Liposomal Drug Delivery to Diverse Tumor Lesions in HER2-Positive Breast Cancer Patients. Annals of Oncology. Elsevier; 2014;25:i19. https://doi.org/10.1093/annonc/mdu068.1
117. Fraguas-Sánchez AI, Lozza I, Torres-Suárez AI. Actively Targeted Nanomedicines in Breast Cancer: From Pre-Clinal Investigation to Clinic. Cancers. Multidisciplinary Digital Publishing Institute; 2022;14:1198. https://doi.org/10.3390/cancers14051198
118. Lee H, Gaddy D, Ventura M, Bernards N, de Souza R, Kirpotin D, et al. Companion Diagnostic 64Cu-Liposome Positron Emission Tomography Enables Characterization of Drug Delivery to Tumors and Predicts Response to Cancer Nanomedicines. Theranostics. 2018;8:2300–12. https://doi.org/10.7150/thno.21670
119. Research C for DE and. FDA D.I.S.C.O. Burst Edition: FDA approval of Fyarro (sirolimus protein-bound particles for injectable suspension (albumin-bound)) for locally advanced unresectable or metastatic malignant perivascular epithelioid cell tumor. FDA [Internet]. FDA; 2024 [cited 2025 Oct 25]; https://www.fda.gov/drugs/resources-information-approved-drugs/fda-disco-burst-edition-fda-approval-fyarro-sirolimus-protein-bound-particles-injectable-suspension. Accessed 25 Oct 2025
120. Weiss GJ, Chao J, Neidhart JD, Ramanathan RK, Bassett D, Neidhart JA, et al. First-in-human phase 1/2a trial of CRLX101, a cyclodextrin-containing polymer-camptothecin nanopharmaceutical in patients with advanced solid tumor malignancies. Invest New Drugs. 2013;31:986–1000. https://doi.org/10.1007/s10637-012-9921-8
121. Serrano-Martínez A, Victoria-Montesinos D, García-Muñoz AM, Hernández-Sánchez P, Lucas-Abellán C, González-Louzao R. A Systematic Review of Clinical Trials on the Efficacy and Safety of CRLX101 Cyclodextrin-Based Nanomedicine for Cancer Treatment. Pharmaceutics. Multidisciplinary Digital Publishing Institute; 2023;15:1824. https://doi.org/10.3390/pharmaceutics15071824
122. Shamaeizadeh N, Sadeghi E, Varshosaz J. Clinical Outcomes and Effectiveness of CRLX101 for Solid Tumors: A Systematic Review and Meta-analysis. Current Medicinal Chemistry. Bentham Science Publishers; 2025;32:3850–60. https://doi.org/10.2174/0109298673263933231206101556
123. Sa P, Mohapatra P, Swain SS, Khuntia A, Sahoo SK. Phytochemical-Based Nanomedicine for Targeting Tumor Microenvironment and Inhibiting Cancer Chemoresistance: Recent Advances and Pharmacological Insights. Mol Pharmaceutics. American Chemical Society; 2023;20:5254–77. https://doi.org/10.1021/acs.molpharmaceut.3c00286

124. Von Hoff DD, Mita MM, Ramanathan RK, Weiss GJ, Mita AC, LoRusso PM, et al. Phase I Study of PSMA-Targeted Docetaxel-Containing Nanoparticle BIND-014 in Patients with Advanced Solid Tumors. Clin Cancer Res. 2016;22:3157–63. https://doi.org/10.1158/1078-0432.CCR-15-2548
125. Bradley CA. Efficacy of a PSMA-targeted nanoparticle. Nat Rev Urol. Nature Publishing Group; 2018;15:590–1. https://doi.org/10.1038/s41585-018-0067-0
126. Kumarasamy RV, Natarajan PM, Umapathy VR, Roy JR, Mironescu M, Palanisamy CP. Clinical applications and therapeutic potentials of advanced nanoparticles: a comprehensive review on completed human clinical trials. Front Nanotechnol [Internet]. Frontiers; 2024 [cited 2025 Aug 21];6. https://doi.org/10.3389/fnano.2024.1479993
127. Sharma B, Crist RM, Adiseshaiah PP. Nanotechnology as a Delivery Tool for Precision Cancer Therapies. AAPS J. 2017;19:1632–42. https://doi.org/10.1208/s12248-017-0152-y
128. Kumthekar P, Rademaker A, Ko C, Dixit K, Schwartz MA, Sonabend AM, et al. A phase 0 first-in-human study using NU-0129: A gold base spherical nucleic acid (SNA) nanoconjugate targeting BCL2L12 in recurrent glioblastoma patients. JCO. Wolters Kluwer; 2019;37:3012–3012. https://doi.org/10.1200/JCO.2019.37.15_suppl.3012
129. Aborig M, Wettig S, Osei E, Alsefaou M. A Comprehensive Review of Gold Nanoparticles in Clinical Trials: Efficacy, Safety and Future Directions [Internet]. ChemRxiv; 2025 [cited 2025 Aug 21]. https://doi.org/10.26434/chemrxiv-2025-d6dw4
130. Gawel AM, Singh R, Debinski W. Metal-Based Nanostructured Therapeutic Strategies for Glioblastoma Treatment—An Update. Biomedicines. Multidisciplinary Digital Publishing Institute; 2022;10:1598. https://doi.org/10.3390/biomedicines10071598
131. Johannsen M, Thiesen B, Wust P, Jordan A. Magnetic nanoparticle hyperthermia for prostate cancer. International Journal of Hyperthermia. Taylor & Francis; 2010;26:790–5. https://doi.org/10.3109/02656731003745740
132. Sikorska M, Domanski G, Bamburowicz-Klimkowska M, Kasprzak A, Nowicka AM, Ruzycka-Ayoush M, et al. Studies on the thermal sensitivity of lung cancer cells exposed to an alternating magnetic field and magnesium-doped maghemite nanoparticles. Cancer Nano. 2024;15:38. https://doi.org/10.1186/s12645-024-00276-0
133. Żuk M, Gawęda W, Majkowska-Pilip A, Osial M, Wolski M, Bilewicz A, et al. Hybrid Radiobioconjugated Superparamagnetic Iron Oxide-Based Nanoparticles for Multimodal Cancer Therapy. Pharmaceutics. Multidisciplinary Digital Publishing Institute; 2021;13:1843. https://doi.org/10.3390/pharmaceutics13111843
134. Chandrasekharan P, Tay ZW, Hensley D, Zhou XY, Fung BK, Colson C, et al. Using magnetic particle imaging systems to localize and guide magnetic hyperthermia treatment: tracers, hardware, and future medical applications. Theranostics. 2020;10:2965–81. https://doi.org/10.7150/thno.40858
135. Ravichandran G, Rengan AK. Aptamer-Mediated Nanotheranostics for Cancer Treatment: A Review. ACS Appl Nano Mater. American Chemical Society; 2020;3:9542–59. https://doi.org/10.1021/acsanm.0c01785
136. Sivakumar B, Aswathy RG, Nagaoka Y, Iwai S, Venugopal K, Kato K, et al. Aptamer conjugated theragnostic multifunctional magnetic nanoparticles as a nanoplatform for pancreatic cancer therapy. RSC Adv. The Royal Society of Chemistry; 2013;3:20579–98. https://doi.org/10.1039/C3RA42645A

137. Zhou G, Latchoumanin O, Bagdesar M, Hebbard L, Duan W, Liddle C, et al. Aptamer-Based Therapeutic Approaches to Target Cancer Stem Cells. Theranostics. 2017;7:3948–61. https://doi.org/10.7150/thno.20725
138. Govindan Navaneeth A, Karthikeyan S. A comprehensive investigation of the biophysical approach for aptamer functionalized nanoparticles in cancer therapy: a review. RSC Pharmaceutics. Royal Society of Chemistry; 2024;1:879–903. https://doi.org/10.1039/D3PM00027C
139. S. Wadajkar A, U. Menon J, Kadapure T, T. Tran R, Yang J, T. Nguyen K. Design and Application of Magnetic-Based Theranostic Nanoparticle Systems. Recent Patents on Biomedical Engineering. 2013;6:47–57.
140. Jo SD, Ku SH, Won Y-Y, Kim SH, Kwon IC. Targeted Nanotheranostics for Future Personalized Medicine: Recent Progress in Cancer Therapy. Theranostics. 2016;6:1362–77. https://doi.org/10.7150/thno.15335
141. Arpicco S, Milla P, Stella B, Dosio F. Hyaluronic Acid Conjugates as Vectors for the Active Targeting of Drugs, Genes and Nanocomposites in Cancer Treatment. Molecules. Multidisciplinary Digital Publishing Institute; 2014;19:3193–230. https://doi.org/10.3390/molecules19033193
142. Mochida Y, Cabral H, Kataoka K. Polymeric micelles for targeted tumor therapy of platinum anticancer drugs. Expert Opinion on Drug Delivery. Taylor & Francis; 2017;14:1423–38. https://doi.org/10.1080/17425247.2017.1307338
143. Uchino H, Matsumura Y, Negishi T, Koizumi F, Hayashi T, Honda T, et al. Cisplatin-incorporating polymeric micelles (NC-6004) can reduce nephrotoxicity and neurotoxicity of cisplatin in rats. Br J Cancer. Nature Publishing Group; 2005;93:678–87. https://doi.org/10.1038/sj.bjc.6602772
144. In GK, Nieva J. Emerging chemotherapy agents in lung cancer: nanoparticles therapeutics for non-small cell lung cancer. Translational Cancer Research [Internet]. AME Publishing Company; 2015 [cited 2025 Aug 21];4. https://doi.org/10.3978/j.issn.2218-676X.2015.08.05
145. Valatabar N, Oroojalian F, Kazemzadeh M, Mokhtarzadeh AA, Safaralizadeh R, Sahebkar A. Recent advances in gene delivery nanoplatforms based on spherical nucleic acids. J Nanobiotechnol. 2024;22:386. https://doi.org/10.1186/s12951-024-02648-5
146. Zhu M, Wang S. Functional Nucleic-Acid-Decorated Spherical Nanoparticles: Preparation Strategies and Current Applications in Cancer Therapy. Small Science. 2021;1:2000056. https://doi.org/10.1002/smsc.202000056
147. Mahajan AS, Stegh AH. Spherical Nucleic Acids as Precision Therapeutics for the Treatment of Cancer—From Bench to Bedside. Cancers. Multidisciplinary Digital Publishing Institute; 2022;14:1615. https://doi.org/10.3390/cancers14071615
148. Tao Z, Zhang H, Wu S, Zhang J, Cheng Y, Lei L, et al. Spherical nucleic acids: emerging amplifiers for therapeutic nanoplatforms. Nanoscale. The Royal Society of Chemistry; 2024;16:4392–406. https://doi.org/10.1039/D3NR05971E
149. Tommasini-Ghelfi S, Lee A, Mirkin CA, Stegh AH. Synthesis, Physicochemical, and Biological Evaluation of Spherical Nucleic Acids for RNAi-Based Therapy in Glioblastoma. In: Dinesh Kumar L, editor. RNA Interference and Cancer Therapy: Methods and Protocols [Internet]. New York, NY: Springer; 2019 [cited 2025 Aug 21]. p. 371–91. https://doi.org/10.1007/978-1-4939-9220-1_25

150. Grapsa D, Syrigos K, Saif MW. Nanoliposomal irinotecan for treating pancreatic cancer. Expert Opinion on Orphan Drugs. Taylor & Francis; 2016;4:541–7. https://doi.org/10.1517/21678707.2016.1169171
151. Thomas A, Pommier Y. Targeting Topoisomerase I in the Era of Precision Medicine. Clin Cancer Res. 2019;25:6581–9. https://doi.org/10.1158/1078-0432.CCR-19-1089
152. Anselmo AC, Mitragotri S. Nanoparticles in the clinic: An update. Bioengineering & Translational Medicine. 2019;4:e10143. https://doi.org/10.1002/btm2.10143
153. Blocker S. Pet Imaging Of Early Therapeutic Response In Solid Tumors. Wayne State University Dissertations [Internet]. 2017; https://digitalcommons.wayne.edu/oa_dissertations/1785
154. Duncan R, Vicent MJ. Do HPMA copolymer conjugates have a future as clinically useful nanomedicines? A critical overview of current status and future opportunities. Advanced Drug Delivery Reviews. 2010;62:272–82. https://doi.org/10.1016/j.addr.2009.12.005
155. Parveen S, Arjmand F, Tabassum S. Clinical developments of antitumor polymer therapeutics. RSC Adv. 2019;9:24699–721. https://doi.org/10.1039/C9RA04358F
156. Gupta C, Naik I, Menon M, Ambre P, Coutinho E. A Review on Exploring the Opportunities of Polymer Drug Conjugated Systems for Targeted Cancer Treatment. Current Drug Delivery. Bentham Science Publishers; 2023;20:8–30. https://doi.org/10.2174/1567201819666220408095510
157. Zhang H, Qian C. Oligonucleotide-Based Modulation of Macrophage Polarization: Emerging Strategies in Immunotherapy. [cited 2025 Aug 22]; https://www.authorea.com/doi/full/10.22541/au.172115373.35217972?commit=84f0609612a3e020ae1f5c7a6a858c2d8ff017b8. Accessed 22 Aug 2025
158. Çakan E, Lara OD, Szymanowska A, Bayraktar E, Chavez-Reyes A, Lopez-Berestein G, et al. Therapeutic Antisense Oligonucleotides in Oncology: From Bench to Bedside. Cancers. Multidisciplinary Digital Publishing Institute; 2024;16:2940. https://doi.org/10.3390/cancers16172940
159. Guo R, Wang R, Zhang W, Li Y, Wang Y, Wang H, et al. Macrophage Polarisation in the Tumour Microenvironment: Recent Research Advances and Therapeutic Potential of Different Macrophage Reprogramming. Cancer Control. SAGE Publications Inc; 2025;32:10732748251316604. https://doi.org/10.1177/10732748251316604
160. M.D. Anderson Cancer Center. Phase I Study of Mesenchymal Stromal Cells-Derived Exosomes With KrasG12D siRNA for Metastatic Pancreas Cancer Patients Harboring KrasG12D Mutation [Internet]. clinicaltrials.gov; 2025 Apr. Report No.: NCT03608631. https://clinicaltrials.gov/study/NCT03608631. Accessed 1 Aug 2025
161. LeBleu VS, Smaglo BG, Mahadevan KK, Kirtley ML, McAndrews KM, Mendt M, et al. KRASG12D-Specific Targeting with Engineered Exosomes Reprograms the Immune Microenvironment to Enable Efficacy of Immune Checkpoint Therapy in PDAC Patients [Internet]. medRxiv; 2025 [cited 2025 Aug 22]. p. 2025.03.03.25322827. https://doi.org/10.1101/2025.03.03.25322827
162. Surana R, LeBleu VS, Lee JJ, Smaglo BG, Zhao D, Lee MS, et al. Phase I study of mesenchymal stem cell (MSC)-derived exosomes with KRASG12D siRNA in patients with metastatic pancreatic cancer harboring a KRASG12D mutation. JCO. Wolters Kluwer; 2022;40:TPS633–TPS633. https://doi.org/10.1200/JCO.2022.40.4_suppl.TPS633

163. Won E-J, Park H, Yoon T-J, Cho Y-S. Gene Therapy Using Nanocarriers for Pancreatic Ductal Adenocarcinoma: Applications and Challenges in Cancer Therapeutics. Pharmaceutics. 2022;14:137. https://doi.org/10.3390/pharmaceutics14010137
164. Hauck ML, LaRue SM, Petros WP, Poulson JM, Yu D, Spasojevic I, et al. Phase I Trial of Doxorubicin-Containing Low Temperature Sensitive Liposomes in Spontaneous Canine Tumors. Clin Cancer Res. 2006;12:4004–10. https://doi.org/10.1158/1078-0432.CCR-06-0226
165. Kozak A, Lavrih E, Mikhaylov G, Turk B, Vasiljeva O. Navigating the Clinical Landscape of Liposomal Therapeutics in Cancer Treatment. Pharmaceutics. 2025;17:276. https://doi.org/10.3390/pharmaceutics17020276
166. Borys N, Dewhirst MW. Drug development of lyso-thermosensitive liposomal doxorubicin: Combining hyperthermia and thermosensitive drug delivery. Advanced Drug Delivery Reviews. 2021;178:113985. https://doi.org/10.1016/j.addr.2021.113985
167. Williamson SK, Johnson GA, Maulhardt HA, Moore KM, McMeekin DS, Schulz TK, et al. A phase I study of intraperitoneal nanoparticulate paclitaxel (Nanotax®) in patients with peritoneal malignancies. Cancer Chemother Pharmacol. 2015;75:1075–87. https://doi.org/10.1007/s00280-015-2737-4
168. Desale SS, Soni KS, Romanova S, Cohen SM, Bronich TK. Targeted delivery of platinum-taxane combination therapy in ovarian cancer. Journal of Controlled Release. 2015;220:651–9. https://doi.org/10.1016/j.jconrel.2015.09.007
169. Hyldbakk A, Fleten KG, Snipstad S, Åslund AKO, Davies C de L, Flatmark K, et al. Intraperitoneal administration of cabazitaxel-loaded nanoparticles in peritoneal metastasis models. Nanomedicine: Nanotechnology, Biology and Medicine. 2023;48:102656. https://doi.org/10.1016/j.nano.2023.102656
170. Breusa S, Zilio S, Catania G, Bakrin N, Kryza D, Lollo G. Localized chemotherapy approaches and advanced drug delivery strategies: a step forward in the treatment of peritoneal carcinomatosis from ovarian cancer. Front Oncol. 2023;13:1125868. https://doi.org/10.3389/fonc.2023.1125868
171. Rietveld PCS, Guchelaar NAD, Sassen SDT, Koch BCP, Mathijssen RHJ, Koolen SLW. A Clinical Pharmacological Perspective on Intraperitoneal Chemotherapy. Drugs. 2025;85:931–43. https://doi.org/10.1007/s40265-025-02195-9
172. Reid G, Johnson TG, van Zandwijk N. Manipulating microRNAs for the Treatment of Malignant Pleural Mesothelioma: Past, Present and Future. Front Oncol [Internet]. Frontiers; 2020 [cited 2025 Aug 22];10. https://doi.org/10.3389/fonc.2020.00105
173. O'Neill CP, Dwyer RM. Nanoparticle-Based Delivery of Tumor Suppressor microRNA for Cancer Therapy. Cells. Multidisciplinary Digital Publishing Institute; 2020;9:521. https://doi.org/10.3390/cells9020521
174. Romano G, Acunzo M, Nana-Sinkam P. microRNAs as Novel Therapeutics in Cancer. Cancers. Multidisciplinary Digital Publishing Institute; 2021;13:1526. https://doi.org/10.3390/cancers13071526
175. Reda El Sayed S, Cristante J, Guyon L, Denis J, Chabre O, Cherradi N. MicroRNA Therapeutics in Cancer: Current Advances and Challenges. Cancers. Multidisciplinary Digital Publishing Institute; 2021;13:2680. https://doi.org/10.3390/cancers13112680
176. Tolcher AW, Papadopoulos KP, Patnaik A, Rasco DW, Martinez D, Wood DL, et al. Safety and activity of DCR-MYC, a first-in-class Dicer-substrate small interfering RNA (DsiRNA) targeting MYC, in a phase I study in patients with advanced solid tumors. JCO. Wolters Kluwer; 2015;33:11006–11006. https://doi.org/10.1200/jco.2015.33.15_suppl.11006

177. Habib S, Ariatti M, Singh M. Anti-c-myc RNAi-Based Onconanotherapeutics. Biomedicines. Multidisciplinary Digital Publishing Institute; 2020;8:612. https://doi.org/10.3390/biomedicines8120612
178. He H, Liu L, Morin EE, Liu M, Schwendeman A. Survey of Clinical Translation of Cancer Nanomedicines—Lessons Learned from Successes and Failures. Acc Chem Res. American Chemical Society; 2019;52:2445–61. https://doi.org/10.1021/acs.accounts.9b00228
179. El-Readi MZ, Althubiti MA. Cancer Nanomedicine: A New Era of Successful Targeted Therapy. Journal of Nanomaterials. 2019;2019:4927312. https://doi.org/10.1155/2019/4927312
180. Libutti SK, Paciotti GF, Byrnes AA, Alexander HR Jr, Gannon WE, Walker M, et al. Phase I and Pharmacokinetic Studies of CYT-6091, a Novel PEGylated Colloidal Gold-rhTNF Nanomedicine. Clin Cancer Res. 2010;16:6139–49. https://doi.org/10.1158/1078-0432.CCR-10-0978
181. Alexis F, Pridgen EM, Langer R, Farokhzad OC. Nanoparticle Technologies for Cancer Therapy. In: Schäfer-Korting M, editor. Drug Delivery [Internet]. Berlin, Heidelberg: Springer; 2010 [cited 2025 Aug 22]. p. 55–86. https://doi.org/10.1007/978-3-642-00477-3_2
182. Gerspach J, Wajant H, Pfizenmaier K. Death Ligands Designed to Kill: Development and Application of Targeted Cancer Therapeutics Based on Proapoptotic TNF Family Ligands. In: Kalthoff H, editor. Death Receptors and Cognate Ligands in Cancer [Internet]. Berlin, Heidelberg: Springer; 2009 [cited 2025 Aug 22]. p. 241–73. https://doi.org/10.1007/400_2008_22
183. Fu Y, Tang R, Zhao X. Engineering cytokines for cancer immunotherapy: a systematic review. Front Immunol [Internet]. Frontiers; 2023 [cited 2025 Aug 22];14. https://doi.org/10.3389/fimmu.2023.1218082
184. Malik MA, Hashmi AA, Al-Bogami AS, Wani MY. Harnessing the power of gold: advancements in anticancer gold complexes and their functionalized nanoparticles. J Mater Chem B. The Royal Society of Chemistry; 2024;12:552–76. https://doi.org/10.1039/D3TB01976D
185. Beg MS, Brenner AJ, Sachdev J, Borad M, Kang Y-K, Stoudemire J, et al. Phase I study of MRX34, a liposomal miR-34a mimic, administered twice weekly in patients with advanced solid tumors. Invest New Drugs. 2017;35:180–8. https://doi.org/10.1007/s10637-016-0407-y
186. Hong DS, Kang Y-K, Borad M, Sachdev J, Ejadi S, Lim HY, et al. Phase 1 study of MRX34, a liposomal miR-34a mimic, in patients with advanced solid tumours. Br J Cancer. Nature Publishing Group; 2020;122:1630–7. https://doi.org/10.1038/s41416-020-0802-1
187. Seyhan AA. Trials and Tribulations of MicroRNA Therapeutics. International Journal of Molecular Sciences. Multidisciplinary Digital Publishing Institute; 2024;25:1469. https://doi.org/10.3390/ijms25031469
188. Tinkaya M, Baran Y. MicroRNAs and Long Non-coding RNAs as Novel Targets in Anti-cancer Drug Development. Current Pharmaceutical Biotechnology. Bentham Science Publishers; 2023;24:913–25. https://doi.org/10.2174/1389201023666220803150431
189. Cadzow L, Arnold K, Thrasher D, Nolan J, Horhota A, Lewis-Clark E, et al. Abstract C184: BIND-510 improves the pharmacokinetics, tolerability, tumor accumulation and tumor growth inhibition in preclinical models of cancer compared to vincristine sulfate. Mol Cancer Ther. 2015;14:C184. https://doi.org/10.1158/1535-7163.TARG-15-C184
190. Shahin M, Alzahrani O, Alzhrani M. Current development in vincristine nanoformulations. IJMDC. 2020;1292–300. https://doi.org/10.24911/IJMDC.51-1591859609
191. Pooja D, Kulhari H, Adams DJ, Sistla R. Formulation and dosage of therapeutic nanosuspension for active targeting of docetaxel (WO 2014210485A1). Expert Opinion on

Therapeutic Patents. Taylor & Francis; 2016;26:745–9. https://doi.org/10.1080/13543776.2016.1180365
192. Sitia L, Sevieri M, Signati L, Bonizzi A, Chesi A, Mainini F, et al. HER-2-Targeted Nanoparticles for Breast Cancer Diagnosis and Treatment. Cancers. Multidisciplinary Digital Publishing Institute; 2022;14:2424. https://doi.org/10.3390/cancers14102424
193. Ughetto S, Giordano S. PERSONALIZED THERAPEUTIC STRATEGIES AND PREDICTIVE BIOMARKERS TO OVERCOME HER2 RESISTANCE IN GASTRIC CANCER.
194. Barnaby SN, Sita TL, Petrosko SH, Stegh AH, Mirkin CA. Therapeutic Applications of Spherical Nucleic Acids. In: Mirkin CA, Meade TJ, Petrosko SH, Stegh AH, editors. Nanotechnology-Based Precision Tools for the Detection and Treatment of Cancer [Internet]. Cham: Springer International Publishing; 2015 [cited 2025 Aug 22]. p. 23–50. https://doi.org/10.1007/978-3-319-16555-4_2
195. Valle JW, Armstrong A, Newman C, Alakhov V, Pietrzynski G, Brewer J, et al. A phase 2 study of SP1049C, doxorubicin in P-glycoprotein-targeting pluronics, in patients with advanced adenocarcinoma of the esophagus and gastroesophageal junction. Invest New Drugs. 2011;29:1029–37. https://doi.org/10.1007/s10637-010-9399-1
196. Danson S, Ferry D, Alakhov V, Margison J, Kerr D, Jowle D, et al. Phase I dose escalation and pharmacokinetic study of pluronic polymer-bound doxorubicin (SP1049C) in patients with advanced cancer. Br J Cancer. Nature Publishing Group; 2004;90:2085–91. https://doi.org/10.1038/sj.bjc.6601856
197. Alakhov V, Kabanov A. Block Copolymer-Based Formulations of Doxorubicin Effective Against Drug Resistant Tumours. In: Chiellini E, Sunamoto J, Migliaresi C, Ottenbrite RM, Cohn D, editors. Biomedical Polymers and Polymer Therapeutics [Internet]. Boston, MA: Springer US; 2002 [cited 2025 Aug 22]. p. 121–37. https://doi.org/10.1007/0-306-46842-5_10
198. Alakhov V, Klinski E, Li S, Pietrzynski G, Venne A, Batrakova E, et al. Block copolymer-based formulation of doxorubicin. From cell screen to clinical trials. Colloids and Surfaces B: Biointerfaces. 1999;16:113–34. https://doi.org/10.1016/S0927-7765(99)00064-8
199. Armand JP, Ducreux M, Mahjoubi M, Abigerges D, Bugat R, Chabot G, et al. CPT-11 (Irinotecan) in the treatment of colorectal cancer. European Journal of Cancer. 1995;31:1283–7. https://doi.org/10.1016/0959-8049(95)00212-2
200. Bleiberg H. CPT-11 in gastrointestinal cancer. European Journal of Cancer. 1999;35:371–9. https://doi.org/10.1016/S0959-8049(98)00423-7
201. Wagener DJTh, Verdonk HER, Dirix LY, Catimel G, Siegenthaler P, Buitenhuis M, et al. Phase II trial of CPT-11 in patients with advanced pancreatic cancer, an EORTC early clinical trials group study. Annals of Oncology. 1995;6:129–32. https://doi.org/10.1093/oxfordjournals.annonc.a059107
202. Yi B-R, Kim SU, Choi K-C. Co-treatment with therapeutic neural stem cells expressing carboxyl esterase and CPT-11 inhibit growth of primary and metastatic lung cancers in mice. Oncotarget. 2014;5:12835–48. https://doi.org/10.18632/oncotarget.2547
203. Stathopoulos GP, Rigatos SK, Dimopoulos MA, Giannakakis T, Foutzilas G, Kouroussis C, et al. Treatment of pancreatic cancer with a combination of irinotecan (CPT-11) and gemcitabine: a multicenter phase II study by the Greek Cooperative Group for Pancreatic Cancer. Annals of Oncology. 2003;14:388–94. https://doi.org/10.1093/annonc/mdg109
204. Hussein L, Moaness M, Mabrouk M, Farahat MG, Beherei HH. Advancements in mesoporous bioactive glasses for effective bone cancer therapy: Recent developments and future perspectives. Biomaterials and Biosystems. 2025;17:100108. https://doi.org/10.1016/j.bbiosy.2025.100108

205. Borges R, Pelosine AM, de Souza ACS, Machado J, Justo GZ, Gamarra LF, et al. Bioactive Glasses as Carriers of Cancer-Targeted Drugs: Challenges and Opportunities in Bone Cancer Treatment. Materials. Multidisciplinary Digital Publishing Institute; 2022;15:9082. https://doi.org/10.3390/ma15249082
206. Sharifi E, Bigham A, Yousefiasl S, Trovato M, Ghomi M, Esmaeili Y, et al. Mesoporous Bioactive Glasses in Cancer Diagnosis and Therapy: Stimuli-Responsive, Toxicity, Immunogenicity, and Clinical Translation. Advanced Science. 2022;9:2102678. https://doi.org/10.1002/advs.202102678
207. Vallejo FA, Sigdel G, Veliz EA, Leblanc RM, Vanni S, Graham RM. Carbon Dots in Treatment of Pediatric Brain Tumors: Past, Present, and Future Directions. International Journal of Molecular Sciences. Multidisciplinary Digital Publishing Institute; 2023;24:9562. https://doi.org/10.3390/ijms24119562
208. Pardo J, Peng Z, Leblanc RM. Cancer Targeting and Drug Delivery Using Carbon-Based Quantum Dots and Nanotubes. Molecules. Multidisciplinary Digital Publishing Institute; 2018;23:378. https://doi.org/10.3390/molecules23020378
209. Anand R, Kumar L, Mohan L, Bharadvaja N. Nano-inspired smart medicines targeting brain cancer: diagnosis and treatment. J Biol Inorg Chem. 2023;28:1–15. https://doi.org/10.1007/s00775-022-01981-0
210. Mukherjee A, Sarkar N. Recent Developments in Quantum Dots Technologies as Effective Theranostic Tools Against Cancer. In: Barik P, Mondal S, editors. Application of Quantum Dots in Biology and Medicine: Recent Advances [Internet]. Singapore: Springer Nature; 2022 [cited 2025 Aug 22]. p. 103–23. https://doi.org/10.1007/978-981-19-3144-4_6
211. Mamot C, Drummond DC, Greiser U, Hong K, Kirpotin DB, Marks JD, et al. Epidermal Growth Factor Receptor (EGFR)-targeted Immunoliposomes Mediate Specific and Efficient Drug Delivery to EGFR- and EGFRvIII-overexpressing Tumor Cells1. Cancer Res. 2003;63:3154–61.
212. Lukianova-Hleb EY, Ren X, Townley D, Wu X, Kupferman ME, Lapotko DO. Plasmonic Nanobubbles Rapidly Detect and Destroy Drug-Resistant Tumors. Theranostics. 2012;2:976–87. https://doi.org/10.7150/thno.5116
213. Mondal G, Almawash S, Chaudhary AK, Mahato RI. EGFR-Targeted Cationic Polymeric Mixed Micelles for Codelivery of Gemcitabine and miR-205 for Treating Advanced Pancreatic Cancer. Mol Pharmaceutics. American Chemical Society; 2017;14:3121–33. https://doi.org/10.1021/acs.molpharmaceut.7b00355
214. Sivadasan D, Sultan MH, Madkhali O, Almoshari Y, Thangavel N. Polymeric Lipid Hybrid Nanoparticles (PLNs) as Emerging Drug Delivery Platform—A Comprehensive Review of Their Properties, Preparation Methods, and Therapeutic Applications. Pharmaceutics. Multidisciplinary Digital Publishing Institute; 2021;13:1291. https://doi.org/10.3390/pharmaceutics13081291
215. Alsaab HO, Alharbi FD, Alhibs AS, Alanazi NB, Alshehri BY, Saleh MA, et al. PLGA-Based Nanomedicine: History of Advancement and Development in Clinical Applications of Multiple Diseases. Pharmaceutics. Multidisciplinary Digital Publishing Institute; 2022;14:2728. https://doi.org/10.3390/pharmaceutics14122728
216. Jia W, Wu Y, Xie Y, Yu M, Chen Y. Advanced Polymeric Nanoparticles for Cancer Immunotherapy: Materials Engineering, Immunotherapeutic Mechanism and Clinical Translation. Advanced Materials. 2025;37:2413603. https://doi.org/10.1002/adma.202413603
217. Parvin N, Aslam M, Joo SW, Mandal TK. Nano-Phytomedicine: Harnessing Plant-Derived Phytochemicals in Nanocarriers for Targeted Human Health Applications. Molecules. 2025;30:3177. https://doi.org/10.3390/molecules30153177

218. Wahnou H, El Kebbaj R, Liagre B, Sol V, Limami Y, Duval RE. Curcumin-Based Nanoparticles: Advancements and Challenges in Tumor Therapy. Pharmaceutics. Multidisciplinary Digital Publishing Institute; 2025;17:114. https://doi.org/10.3390/pharmaceutics17010114
219. Cordani M, Condello M, Meschini S, Strippoli R. Autophagy Modulation in Cancer Treatment Utilizing Nanomaterials and Nanocarriers. Frontiers Media SA; 2023.
220. Haider M, Elsherbeny A, Pittalà V, Consoli V, Alghamdi MA, Hussain Z, et al. Nanomedicine Strategies for Management of Drug Resistance in Lung Cancer. International Journal of Molecular Sciences. Multidisciplinary Digital Publishing Institute; 2022;23:1853. https://doi.org/10.3390/ijms23031853
221. Khan MdA, Zafaryab Md, Mehdi SH, Ahmad I, Rizvi MMA. Characterization and anti-proliferative activity of curcumin loaded chitosan nanoparticles in cervical cancer. International Journal of Biological Macromolecules. 2016;93:242–53. https://doi.org/10.1016/j.ijbiomac.2016.08.050
222. Azmoonfar R, Moslehi M, Shahbazi-Gahrouei D, Shiri E, Azizi M. Radioprotection and enhanced efficacy by curcumin-loaded chitosan nanoparticles in mitigating radiation-induced liver injury. Biochemical and Biophysical Research Communications. 2025;753:151512. https://doi.org/10.1016/j.bbrc.2025.151512
223. Harakeh S, Saber SH, Al-Raddadi R, Alamri T, Al-Jaouni S, Qari M, et al. Novel curcumin nanoformulation induces apoptosis, and reduces migration and angiogenesis in liver cancer cells. Artificial Cells, Nanomedicine, and Biotechnology. Taylor & Francis; 2023;51:361–70. https://doi.org/10.1080/21691401.2023.2238756
224. Kundu AK, Iyer SV, Chandra S, Adhikari AS, Iwakuma T, Mandal TK. Novel siRNA formulation to effectively knockdown mutant p53 in osteosarcoma. PLOS ONE. Public Library of Science; 2017;12:e0179168. https://doi.org/10.1371/journal.pone.0179168
225. Sindhu RK, Goyal S, Thirumal D, Rani K, Gupta AK, Babu MA, et al. Smart Targeted Novel Nanocarriers-Based Treatment of Arthritis. Nanotechnology and Drug Delivery. Jenny Stanford Publishing; 2024.
226. Song S, Xia H, Guo M, Wang S, Zhang S, Ma P, et al. Role of macrophage in nanomedicine-based disease treatment. Drug Delivery. Taylor & Francis; 2021;28:752–66. https://doi.org/10.1080/10717544.2021.1909175
227. Izci M, Maksoudian C, Manshian BB, Soenen SJ. The Use of Alternative Strategies for Enhanced Nanoparticle Delivery to Solid Tumors. Chem Rev. American Chemical Society; 2021;121:1746–803. https://doi.org/10.1021/acs.chemrev.0c00779
228. Kiaei SZF, Nouralishahi A, Ghasemirad M, Barkhordar M, Ghaffari S, Kheradjoo H, et al. Advances in natural killer cell therapies for breast cancer. Immunology & Cell Biology. 2023;101:705–26. https://doi.org/10.1111/imcb.12658
229. Dees S, Ganesan R, Singh S, Grewal IS. Bispecific Antibodies for Triple Negative Breast Cancer. Trends in Cancer. Elsevier; 2021;7:162–73. https://doi.org/10.1016/j.trecan.2020.09.004
230. Raju GSR, Pavitra E, Varaprasad GL, Bandaru SS, Nagaraju GP, Farran B, et al. Nanoparticles mediated tumor microenvironment modulation: current advances and applications. J Nanobiotechnol. 2022;20:274. https://doi.org/10.1186/s12951-022-01476-9
231. Guan C, Zhu X, Feng C. DNA Nanodevice-Based Drug Delivery Systems. Biomolecules. Multidisciplinary Digital Publishing Institute; 2021;11:1855. https://doi.org/10.3390/biom11121855

232. Tao Z, Zhang H, Wu S, Zhang J, Cheng Y, Lei L, et al. Spherical nucleic acids: emerging amplifiers for therapeutic nanoplatforms. Nanoscale. The Royal Society of Chemistry; 2024;16:4392–406. https://doi.org/10.1039/D3NR05971E
233. Jiang H, Bao Q, Yang T, Yang M, Mao C. Precision Treatment of Colon Cancer Using Doxorubicin-Loaded Metal–Organic-Framework-Coated Magnetic Nanoparticles. ACS Appl Mater Interfaces [Internet]. American Chemical Society; 2024 [cited 2025 July 9];16:49003–12. https://doi.org/10.1021/acsami.4c08602
234. Bai X, Xu Y, Liu Y. Therapeutic effects of DOX-loaded hydrogel MOF nanocarriers on triple negative breast cancer and derivative design via reinforcement learning. Sci Rep [Internet]. Nature Publishing Group; 2024 [cited 2025 July 9];14:23946. https://doi.org/10.1038/s41598-024-75466-2
235. Lohlamoh W, Soontornworajit B, Rotkrua P. Anti-Proliferative Effect of Doxorubicin-Loaded AS1411 Aptamer on Colorectal Cancer Cell. Asian Pac J Cancer Prev. 2021;22:2209–19. https://doi.org/10.31557/APJCP.2021.22.7.2209
236. Hosseini NF, Amini R, Ramezani M, Saidijam M, Hashemi SM, Najafi R. AS1411 aptamer-functionalized exosomes in the targeted delivery of doxorubicin in fighting colorectal cancer. Biomedicine & Pharmacotherapy. 2022;155:113690. https://doi.org/10.1016/j.biopha.2022.113690
237. Zhu J, Huang H, Dong S, Ge L, Zhang Y. Progress in Aptamer-Mediated Drug Delivery Vehicles for Cancer Targeting and Its Implications in Addressing Chemotherapeutic Challenges. Theranostics. 2014;4:931–44. https://doi.org/10.7150/thno.9663
238. Chandola C, Kalme S, Casteleijn MG, Urtti A, Neerathilingam M. Application of aptamers in diagnostics, drug-delivery and imaging. J Biosci. 2016;41:535–61. https://doi.org/10.1007/s12038-016-9632-y
239. Hu H-H, Wang S-Q, Zhao H, Chen Z-S, Shi X, Chen X-B. $HER2^{+}$ advanced gastric cancer: Current state and opportunities (Review). International Journal of Oncology. Spandidos Publications; 2024;64:1–17. https://doi.org/10.3892/ijo.2024.5624
240. Liu J, Zhu T, Zhao R, Ren W, Zhao F, Liu J. Elucidating molecular mechanisms and therapeutic synergy: irreversible HER2-TKI plus T-Dxd for enhanced anti-HER2 treatment of gastric cancer. Gastric Cancer. 2024;27:495–505. https://doi.org/10.1007/s10120-024-01478-6
241. Huang J, Chen X, Guo J, Song L, Mu Y, Zhao H, et al. Apatinib combined with trastuzumab and albumin-bound paclitaxel for treatment of HER2+ breast cancer with brain metastases resistant to anti-HER2 TKIs: A case report. Oncology Letters. Spandidos Publications; 2023;25:1–6. https://doi.org/10.3892/ol.2022.13642
242. Okines A, Kang V, Walker LN, Pohlmann PR. 125TiP SGNTUC-019: Phase II basket study of tucatinib and trastuzumab in previously treated solid tumours with HER2 alterations: HER2-mutated breast cancer cohort. Annals of Oncology. Elsevier; 2021;32:S74–5. https://doi.org/10.1016/j.annonc.2021.03.139
243. Furugaki K, Fujimura T, Mizuta H, Yoshimoto T, Asakawa T, Yoshimura Y, et al. FGFR blockade inhibits targeted therapy-tolerant persister in basal FGFR1- and FGF2-high cancers with driver oncogenes. npj Precis Onc. Nature Publishing Group; 2023;7:107. https://doi.org/10.1038/s41698-023-00462-0
244. Balar AV, Milowsky MI. Novel Agents in Invasive Urothelial Cancer.
245. Yang X, Su L-J, Rosa FGL, Smith EE, Cho SK, Kavanagh B, et al. Abstract 2728: Thermal ablative therapy with novel gold nanorods in an orthotopic model of urinary bladder cancer. Cancer Res. 2014;74:2728. https://doi.org/10.1158/1538-7445.AM2014-2728

246. Cai H, Wang R, Guo X, Song M, Yan F, Ji B, et al. Combining Gemcitabine-Loaded Macrophage-like Nanoparticles and Erlotinib for Pancreatic Cancer Therapy. Mol Pharm. 2021;18:2495–506. https://doi.org/10.1021/acs.molpharmaceut.0c01225
247. Joshi G, Kumar A, Sawant K. Enhanced bioavailability and intestinal uptake of Gemcitabine HCl loaded PLGA nanoparticles after oral delivery. Eur J Pharm Sci. 2014;60:80–9. https://doi.org/10.1016/j.ejps.2014.04.014
248. Devulapally R, Foygel K, Sekar TV, Willmann JK, Paulmurugan R. Gemcitabine and Antisense-microRNA Co-encapsulated PLGA–PEG Polymer Nanoparticles for Hepatocellular Carcinoma Therapy. ACS Appl Mater Interfaces [Internet]. American Chemical Society; 2016 [cited 2025 July 9];8:33412–22. https://doi.org/10.1021/acsami.6b08153
249. Crumbaker M, Pathmanandavel S, Yam AO, Nguyen A, Ho B, Chan L, et al. Phase I/II Trial of the Combination of 177Lutetium Prostate specific Membrane Antigen 617 and Idronoxil (NOX66) in Men with End-stage Metastatic Castration-resistant Prostate Cancer (LuPIN). European Urology Oncology [Internet]. 2021 [cited 2025 July 9];4:963–70. https://doi.org/10.1016/j.euo.2020.07.002
250. Noxopharm Limited. A Phase 1b/2a Multicenter Study of NOX66 and External Beam Radiotherapy in Patients With Metastatic Castration-resistant Prostate Cancer and Other Solid Tumors [Internet]. clinicaltrials.gov; 2024 May. Report No.: NCT04957290. https://clinicaltrials.gov/study/NCT04957290. Accessed 9 July 2025
251. de Souza PL, Capp AL, Chikhladze N, Mezvrishvili Z, Messina M, Mautner G. Phase I study of a novel S1P inhibitor, NOX66, in combination with radiotherapy in patients with metastatic castration-resistant prostate cancer. JCO. Wolters Kluwer; 2020;38:5533–5533. https://doi.org/10.1200/JCO.2020.38.15_suppl.5533
252. Lee J, Kim T. Current Status and Future Perspectives of Nuclear Medicine in Prostate Cancer from Imaging to Therapy: A Comprehensive Review. Biomedicines. Multidisciplinary Digital Publishing Institute; 2025;13:1132. https://doi.org/10.3390/biomedicines13051132
253. Ginghină O, Hudiță A, Zaharia C, Tsatsakis A, Mezhuev Y, Costache M, et al. Current Landscape in Organic Nanosized Materials Advances for Improved Management of Colorectal Cancer Patients. Materials. Multidisciplinary Digital Publishing Institute; 2021;14:2440. https://doi.org/10.3390/ma14092440
254. Alharbi A. Development of Novel Polymeric Micellar DACHPt for Enhanced Platinum Based Chemotherapy in Colorectal Cancer [Internet]. ERA. 2019 [cited 2025 Aug 22]. https://doi.org/10.7939/r3-j2d6-r603
255. Liu T, Deng Y, Yao J, Xiong H, Yao J. Assembly Simulation and Synergistic Chemotherapy of TPGS Derivative Functionalized Polymersomes in Hepatocellular Carcinoma. Nanomedicine. Taylor & Francis; 2019;14:1707–27. https://doi.org/10.2217/nnm-2019-0024
256. Arya G, Das M, Sahoo SK. Evaluation of curcumin loaded chitosan/PEG blended PLGA nanoparticles for effective treatment of pancreatic cancer. Biomedicine & Pharmacotherapy [Internet]. 2018 [cited 2025 July 9];102:555–66. https://doi.org/10.1016/j.biopha.2018.03.101
257. Liu L, Sun L, Wu Q, Guo W, Li L, Chen Y, et al. Curcumin loaded polymeric micelles inhibit breast tumor growth and spontaneous pulmonary metastasis. Int J Pharm. 2013;443:175–82. https://doi.org/10.1016/j.ijpharm.2012.12.032
258. Prasanth R, Nair G, Girish CM. Enhanced endocytosis of nano-curcumin in nasopharyngeal cancer cells: An atomic force microscopy study. Applied Physics Letters [Internet]. 2011 [cited 2025 July 9];99:163706. https://doi.org/10.1063/1.3653388

259. Basniwal RK, Khosla R, Jain N. Improving the anticancer activity of curcumin using nanocurcumin dispersion in water. Nutr Cancer. 2014;66:1015–22. https://doi.org/10.1080/01635581.2014.936948
260. Hu B, Sun D, Sun C, Sun Y-F, Sun H-X, Zhu Q-F, et al. A polymeric nanoparticle formulation of curcumin in combination with sorafenib synergistically inhibits tumor growth and metastasis in an orthotopic model of human hepatocellular carcinoma. Biochem Biophys Res Commun. 2015;468:525–32. https://doi.org/10.1016/j.bbrc.2015.10.031
261. Chamani F, Sadeghizadeh M, Masoumi M, Babashah S. Evaluation of MiR-34 Family and DNA Methyltransferases 1, 3A, 3B Gene Expression Levels in Hepatocellular Carcinoma Following Treatment with Dendrosomal Nanocurcumin. Asian Pac J Cancer Prev. 2016;17:219–24. https://doi.org/10.7314/apjcp.2016.17.s3.219
262. Al-Obaidy R, Haider AJ, Al-Musawi S, Arsad N. Targeted delivery of paclitaxel drug using polymer-coated magnetic nanoparticles for fibrosarcoma therapy: in vitro and in vivo studies. Sci Rep [Internet]. Nature Publishing Group; 2023 [cited 2025 July 9];13:3180. https://doi.org/10.1038/s41598-023-30221-x
263. Ngema LM, Adeyemi SA, Marimuthu T, Ubanako PN, Ngwa W, Choonara YE. Short Antiangiogenic MMP-2 Peptide-Decorated Conjugated Linoleic Acid-Coated SPIONs for Targeted Paclitaxel Delivery in an A549 Cell Xenograft Mouse Tumor Model. ACS Omega [Internet]. American Chemical Society; 2024 [cited 2025 July 9];9:700–13. https://doi.org/10.1021/acsomega.3c06489
264. Ngema LM, Adeyemi SA, Marimuthu T, Ubanako P, Wamwangi D, Choonara YE. Synthesis of Novel Conjugated Linoleic Acid (CLA)-Coated Superparamagnetic Iron Oxide Nanoparticles (SPIONs) for the Delivery of Paclitaxel with Enhanced In Vitro Anti-Proliferative Activity on A549 Lung Cancer Cells. Pharmaceutics [Internet]. 2022 [cited 2025 July 9];14:829. https://doi.org/10.3390/pharmaceutics14040829
265. Rivera-Rodriguez A, Chiu-Lam A, Morozov VM, Ishov AM, Rinaldi C. Magnetic nanoparticle hyperthermia potentiates paclitaxel activity in sensitive and resistant breast cancer cells. Int J Nanomedicine [Internet]. 2018 [cited 2025 July 9];13:4771–9. https://doi.org/10.2147/IJN.S171130
266. Benjamin AS, Nayak S. Iron oxide nanoparticles coated with bioactive materials: a viable theragnostic strategy to improve osteosarcoma treatment. Discover Nano [Internet]. 2025 [cited 2025 July 9];20:18. https://doi.org/10.1186/s11671-024-04163-w
267. Erkisa M, Ari F, Ulku I, Khodadust R, Yar Y, Yagci Acar H, et al. Etoposide Loaded SPION-PNIPAM Nanoparticles Improve the in vitro Therapeutic Outcome on Metastatic Prostate Cancer Cells via Enhanced Apoptosis. Chem Biodivers. 2020;17:e2000607. https://doi.org/10.1002/cbdv.202000607
268. Ghalehkhondabi V, Fazlali A, Soleymani M. Temperature and pH-responsive PNIPAM@PAA Nanospheres with a Core-Shell Structure for Controlled Release of Doxorubicin in Breast Cancer Treatment. Journal of Pharmaceutical Sciences [Internet]. 2023 [cited 2025 July 9];112:1957–66. https://doi.org/10.1016/j.xphs.2023.04.009
269. Dhamecha D, Le D, Chakravarty T, Perera K, Dutta A, Menon JU. Fabrication of PNIPAm-based Thermoresponsive Hydrogel Microwell Arrays for Tumor Spheroid Formation. Mater Sci Eng C Mater Biol Appl [Internet]. 2021 [cited 2025 July 9];125:112100. https://doi.org/10.1016/j.msec.2021.112100
270. Jin K, Zhang H, Yang Y, Gao Y. Nano-drug delivery systems based on biodegradable polymers for the therapy of gynecological malignancies. International Journal of Polymeric

Materials and Polymeric Biomaterials. Taylor & Francis; 2024;73:1262–77. https://doi.org/10.1080/00914037.2023.2263133

271. Yao S, Li L, Liu C, Wang Z, Liu H, Su X, et al. Novel Multifunctional Magnetic Nanoparticles : An Efficient Theranostic Platform for Magnetic Resonance Imaging and Targeted Therapy of Cervical Cancer [Internet]. Research Square; 2021 [cited 2025 Aug 22]. https://doi.org/10.21203/rs.3.rs-957553/v1

272. Yao S, Li L, Su X, Wang K, Lu Z, Yuan C, et al. Development and evaluation of novel tumor-targeting paclitaxel-loaded nano-carriers for ovarian cancer treatment: in vitro and in vivo. J Exp Clin Cancer Res. 2018;37:29. https://doi.org/10.1186/s13046-018-0700-z

273. Gralewska P, Gajek A, Marczak A, Rogalska A. Targeted Nanocarrier-Based Drug Delivery Strategies for Improving the Therapeutic Efficacy of PARP Inhibitors against Ovarian Cancer. International Journal of Molecular Sciences. Multidisciplinary Digital Publishing Institute; 2024;25:8304. https://doi.org/10.3390/ijms25158304

274. Li Q, Liu X, Yan W, Chen Y. Antitumor effect of poly lactic acid nanoparticles loaded with cisplatin and chloroquine on the oral squamous cell carcinoma. Aging (Albany NY). 2020;13:2593–603. https://doi.org/10.18632/aging.202297

275. Liu D, Zhang Q, Wang J, Fan L, Zhu W, Cai D. Hyaluronic acid-coated single-walled carbon nanotubes loaded with doxorubicin for the treatment of breast cancer. Die Pharmazie - An International Journal of Pharmaceutical Sciences. 2019;74:83–90. https://doi.org/10.1691/ph.2019.8152

276. Gayathri K, Vidya R. Carbon nanomaterials as carriers for the anti-cancer drug doxorubicin: a review on theoretical and experimental studies. Royal Society of Chemistry; 2024 [cited 2025 Aug 22]; https://doi.org/10.1039/D4NA00278D

277. Grushevskaya HV, Krylova NG. Carbon Nanotubes as A High-Performance Platform for Target Delivery of Anticancer Quinones. Current Pharmaceutical Design. Bentham Science Publishers; 2018;24:5207–18. https://doi.org/10.2174/1381612825666190117095132

278. Bhattacharya D, Mukhopadhyay M, Shivam K, Tripathy S, Patra R, Pramanik A. Recent developments in photodynamic therapy and its application against multidrug resistant cancers. Biomed Mater. IOP Publishing; 2023;18:062005. https://doi.org/10.1088/1748-605X/ad02d4

279. Conte C, Ungaro F, Mazzaglia A, Quaglia F. Photodynamic Therapy for Cancer: Principles, Clinical Applications, and Nanotechnological Approaches. In: Alonso MJ, Garcia-Fuentes M, editors. Nano-Oncologicals: New Targeting and Delivery Approaches [Internet]. Cham: Springer International Publishing; 2014 [cited 2025 Aug 22]. p. 123–60. https://doi.org/10.1007/978-3-319-08084-0_5

280. Zhang P, Xiao Y, Sun X, Lin X, Koo S, Yaremenko AV, et al. Cancer nanomedicine toward clinical translation: Obstacles, opportunities, and future prospects. Med. Elsevier; 2023;4:147–67. https://doi.org/10.1016/j.medj.2022.12.001

281. Iranpour S, Bahrami AR, Nekooei S, Sh. Saljooghi A, Matin MM. Improving anti-cancer drug delivery performance of magnetic mesoporous silica nanocarriers for more efficient colorectal cancer therapy. J Nanobiotechnol. 2021;19:314. https://doi.org/10.1186/s12951-021-01056-3

282. Yousefiasl S, Ghovvati M, Alibakhshi A, Azizi M, Samadi P, Kumar A, et al. Smart Mesoporous Silica Nanoparticles in Cancer: Diagnosis, Treatment, Immunogenicity, and Clinical Translation. Small. 2025;21:2408898. https://doi.org/10.1002/smll.202408898

283. Poonia N, Lather V, Pandita D. Mesoporous silica nanoparticles: a smart nanosystem for management of breast cancer. Drug Discovery Today. 2018;23:315–32. https://doi.org/10.1016/j.drudis.2017.10.022

284. Colilla M, Vallet-Regí M. Organically Modified Mesoporous Silica Nanoparticles against Bacterial Resistance. Chem Mater. American Chemical Society; 2023;35:8788–805. https://doi.org/10.1021/acs.chemmater.3c02192
285. Kesse S, Boakye-Yiadom KO, Ochete BO, Opoku-Damoah Y, Akhtar F, Filli MS, et al. Mesoporous Silica Nanomaterials: Versatile Nanocarriers for Cancer Theranostics and Drug and Gene Delivery. Pharmaceutics. Multidisciplinary Digital Publishing Institute; 2019;11:77. https://doi.org/10.3390/pharmaceutics11020077
286. Sengodan N. Enhanced Histopathology Image Feature Extraction using EfficientNet with Dual Attention Mechanisms and CLAHE Preprocessing [Internet]. arXiv; 2024 [cited 2025 July 8]. https://doi.org/10.48550/arXiv.2410.22392
287. Sarvamangala DR, Kulkarni RV. Convolutional neural networks in medical image understanding: a survey. Evol Intell [Internet]. 2022 [cited 2025 July 8];15:1–22. https://doi.org/10.1007/s12065-020-00540-3
288. Salehi AW, Khan S, Gupta G, Alabduallah BI, Almjally A, Alsolai H, et al. A Study of CNN and Transfer Learning in Medical Imaging: Advantages, Challenges, Future Scope. Sustainability [Internet]. Multidisciplinary Digital Publishing Institute; 2023 [cited 2025 July 8];15:5930. https://doi.org/10.3390/su15075930
289. Yadav SS, Jadhav SM. Deep convolutional neural network based medical image classification for disease diagnosis. Journal of Big Data [Internet]. 2019 [cited 2025 July 8];6:113. https://doi.org/10.1186/s40537-019-0276-2
290. Noh S-H. Analysis of Gradient Vanishing of RNNs and Performance Comparison. Information [Internet]. Multidisciplinary Digital Publishing Institute; 2021 [cited 2025 July 8];12:442. https://doi.org/10.3390/info12110442
291. Malashin I, Tynchenko V, Gantimurov A, Nelyub V, Borodulin A. Applications of Long Short-Term Memory (LSTM) Networks in Polymeric Sciences: A Review. Polymers [Internet]. Multidisciplinary Digital Publishing Institute; 2024 [cited 2025 July 8];16:2607. https://doi.org/10.3390/polym16182607
292. Liu X, Liu C, Huang R, Zhu H, Liu Q, Mitra S, et al. Long short-term memory recurrent neural network for pharmacokinetic-pharmacodynamic modeling. Int J Clin Pharmacol Ther. 2021;59:138–46. https://doi.org/10.5414/CP203800
293. Lee Y, Son J, Song M. BertSRC: transformer-based semantic relation classification. BMC Medical Informatics and Decision Making [Internet]. 2022 [cited 2025 July 8];22:234. https://doi.org/10.1186/s12911-022-01977-5
294. Nerella S, Bandyopadhyay S, Zhang J, Contreras M, Siegel S, Bumin A, et al. Transformers and large language models in healthcare: A review. Artificial Intelligence in Medicine [Internet]. 2024 [cited 2025 July 8];154:102900. https://doi.org/10.1016/j.artmed.2024.102900
295. Luo R, Sun L, Xia Y, Qin T, Zhang S, Poon H, et al. BioGPT: generative pre-trained transformer for biomedical text generation and mining. Briefings in Bioinformatics [Internet]. 2022 [cited 2025 July 8];23:bbac409. https://doi.org/10.1093/bib/bbac409
296. Sarumi OA, Heider D. Large language models and their applications in bioinformatics. Computational and Structural Biotechnology Journal [Internet]. 2024 [cited 2025 July 8];23:3498–505. https://doi.org/10.1016/j.csbj.2024.09.031
297. Riddick G, Song H, Ahn S, Walling J, Borges-Rivera D, Zhang W, et al. Predicting in vitro drug sensitivity using Random Forests. Bioinformatics [Internet]. 2011 [cited 2025 July 8];27:220–4. https://doi.org/10.1093/bioinformatics/btq628

298. Rahman R, Dhruba SR, Ghosh S, Pal R. Functional random forest with applications in dose-response predictions. Sci Rep [Internet]. Nature Publishing Group; 2019 [cited 2025 July 8];9:1628. https://doi.org/10.1038/s41598-018-38231-w
299. Lind AP, Anderson PC. Predicting drug activity against cancer cells by random forest models based on minimal genomic information and chemical properties. PLoS One [Internet]. 2019 [cited 2025 July 8];14:e0219774. https://doi.org/10.1371/journal.pone.0219774
300. Li J, Chen A, Liu Z, Wei S, Zhang J, Chen J, et al. Machine learning driven prediction of drug efficacy in lung cancer: based on protein biomarkers and clinical features. Life Sciences [Internet]. 2025 [cited 2025 July 8];375:123706. https://doi.org/10.1016/j.lfs.2025.123706
301. Wu F, Zhang X, Fang Z, Yu X. Support Vector Machine-Based Global Classification Model of the Toxicity of Organic Compounds to Vibrio fischeri. Molecules [Internet]. 2023 [cited 2025 July 8];28:2703. https://doi.org/10.3390/molecules28062703
302. Tharwat A, Moemen YS, Hassanien AE. Classification of toxicity effects of biotransformed hepatic drugs using whale optimized support vector machines. Journal of Biomedical Informatics [Internet]. 2017 [cited 2025 July 8];68:132–49. https://doi.org/10.1016/j.jbi.2017.03.002
303. Rodríguez-Pérez R, Vogt M, Bajorath J. Support Vector Machine Classification and Regression Prioritize Different Structural Features for Binary Compound Activity and Potency Value Prediction. ACS Omega [Internet]. American Chemical Society; 2017 [cited 2025 July 8];2:6371–9. https://doi.org/10.1021/acsomega.7b01079
304. Rahman SM, Lan J, Kaeli D, Dy J, Alshawabkeh A, Gu AZ. Machine Learning-based Biomarkers Identification from Toxicogenomics - Bridging to Regulatory Relevant Phenotypic Endpoints. J Hazard Mater [Internet]. 2022 [cited 2025 July 8];423:127141. https://doi.org/10.1016/j.jhazmat.2021.127141
305. Qin S, Jiang S, Li J, Balaprakash P, Lehn RCV, M. Zavala V. Capturing molecular interactions in graph neural networks: a case study in multi-component phase equilibrium. Digital Discovery [Internet]. Royal Society of Chemistry; 2023 [cited 2025 July 8];2:138–51. https://doi.org/10.1039/D2DD00045H
306. Zhou J, Cui G, Hu S, Zhang Z, Yang C, Liu Z, et al. Graph neural networks: A review of methods and applications. AI Open [Internet]. 2020 [cited 2025 July 8];1:57–81. https://doi.org/10.1016/j.aiopen.2021.01.001
307. Zhang S, Jin Y, Liu T, Wang Q, Zhang Z, Zhao S, et al. SS-GNN: A Simple-Structured Graph Neural Network for Affinity Prediction. ACS Omega [Internet]. American Chemical Society; 2023 [cited 2025 July 8];8:22496–507. https://doi.org/10.1021/acsomega.3c00085
308. Huang K, Xiao C, Glass LM, Zitnik M, Sun J. SkipGNN: predicting molecular interactions with skip-graph networks. Sci Rep [Internet]. Nature Publishing Group; 2020 [cited 2025 July 8];10:21092. https://doi.org/10.1038/s41598-020-77766-9
309. Ochiai T, Inukai T, Akiyama M, Furui K, Ohue M, Matsumori N, et al. Variational autoencoder-based chemical latent space for large molecular structures with 3D complexity. Commun Chem [Internet]. Nature Publishing Group; 2023 [cited 2025 July 8];6:249. https://doi.org/10.1038/s42004-023-01054-6
310. Liu Z, Liu Y, Yu Z, Yang Z, Fu Q, Guo Y, et al. PT-VAE: Variational autoencoder with prior concept transformation. Neurocomputing [Internet]. 2025 [cited 2025 July 8];638:130129. https://doi.org/10.1016/j.neucom.2025.130129
311. Kingma DP, Welling M. An Introduction to Variational Autoencoders. FNT in Machine Learning [Internet]. 2019 [cited 2025 July 8];12:307–92. https://doi.org/10.1561/2200000056

312. Sevgen E, Moller J, Lange A, Parker J, Quigley S, Mayer J, et al. ProT-VAE: Protein Transformer Variational AutoEncoder for Functional Protein Design [Internet]. bioRxiv; 2023 [cited 2025 July 8]. p. 2023.01.23.525232. https://doi.org/10.1101/2023.01.23.525232
313. Simidjievski N, Bodnar C, Tariq I, Scherer P, Andres Terre H, Shams Z, et al. Variational Autoencoders for Cancer Data Integration: Design Principles and Computational Practice. Front Genet [Internet]. 2019 [cited 2025 July 8];10:1205. https://doi.org/10.3389/fgene.2019.01205
314. Lambard G, Yamazaki K, Demura M. Generation of highly realistic microstructural images of alloys from limited data with a style-based generative adversarial network. Sci Rep [Internet]. Nature Publishing Group; 2023 [cited 2025 July 8];13:566. https://doi.org/10.1038/s41598-023-27574-8
315. Rather IH, Kumar S. Generative adversarial network based synthetic data training model for lightweight convolutional neural networks. Multimed Tools Appl [Internet]. 2024 [cited 2025 July 8];83:6249–71. https://doi.org/10.1007/s11042-023-15747-6
316. Chen Y, Yang X-H, Wei Z, Heidari AA, Zheng N, Li Z, et al. Generative Adversarial Networks in Medical Image augmentation: A review. Computers in Biology and Medicine [Internet]. 2022 [cited 2025 July 8];144:105382. https://doi.org/10.1016/j.compbiomed.2022.105382
317. Nezhadettehad A, Zaslavsky A, Rakib A, Loke SW. Uncertainty-Aware Parking Prediction Using Bayesian Neural Networks. Sensors [Internet]. Multidisciplinary Digital Publishing Institute; 2025 [cited 2025 July 8];25:3463. https://doi.org/10.3390/s25113463
318. Gawlikowski J, Tassi CRN, Ali M, Lee J, Humt M, Feng J, et al. A survey of uncertainty in deep neural networks. Artif Intell Rev [Internet]. 2023 [cited 2025 July 8];56:1513–89. https://doi.org/10.1007/s10462-023-10562-9
319. Semenova E, Williams DP, Afzal AM, Lazic SE. A Bayesian neural network for toxicity prediction [Internet]. bioRxiv; 2020 [cited 2025 July 8]. p. 2020.04.28.065532. https://doi.org/10.1101/2020.04.28.065532
320. Surapunt T, Wang S. Ensemble Modeling with a Bayesian Maximal Information Coefficient-Based Model of Bayesian Predictions on Uncertainty Data. Information [Internet]. Multidisciplinary Digital Publishing Institute; 2024 [cited 2025 July 8];15:228. https://doi.org/10.3390/info15040228
321. Usman M, Chen F. Generation and optimization of gold nanoclusters via reinforcement learning. Eur Phys J D [Internet]. 2025 [cited 2025 July 8];79:58. https://doi.org/10.1140/epjd/s10053-025-01006-w
322. Zhou Z, Li X, Zare RN. Optimizing Chemical Reactions with Deep Reinforcement Learning. ACS Cent Sci [Internet]. American Chemical Society; 2017 [cited 2025 July 8];3:1337–44. https://doi.org/10.1021/acscentsci.7b00492
323. Raju RK. Exploring Nanocluster Potential Energy Surfaces via Deep Reinforcement Learning: Strategies for Global Minimum Search. J Phys Chem A [Internet]. American Chemical Society; 2024 [cited 2025 July 8];128:9122–34. https://doi.org/10.1021/acs.jpca.4c04416
324. Akbulut Y, Sengur A, Guo Y, Smarandache F. NS-k-NN: Neutrosophic Set-Based k-Nearest Neighbors Classifier. Symmetry [Internet]. Multidisciplinary Digital Publishing Institute; 2017 [cited 2025 July 8];9:179. https://doi.org/10.3390/sym9090179
325. Cunningham P, Delany SJ. k-Nearest Neighbour Classifiers: 2nd Edition (with Python examples). ACM Comput Surv [Internet]. 2022 [cited 2025 July 8];54:1–25. https://doi.org/10.1145/3459665
326. Guo G, Wang H, Bell D, Bi Y, Greer K. KNN Model-Based Approach in Classification. In: Meersman R, Tari Z, Schmidt DC, editors. On The Move to Meaningful Internet Systems 2003:

CoopIS, DOA, and ODBASE. Berlin, Heidelberg: Springer; 2003. p. 986–96. https://doi.org/10.1007/978-3-540-39964-3_62
327. Uddin S, Haque I, Lu H, Moni MA, Gide E. Comparative performance analysis of K-nearest neighbour (KNN) algorithm and its different variants for disease prediction. Sci Rep [Internet]. Nature Publishing Group; 2022 [cited 2025 July 8];12:6256. https://doi.org/10.1038/s41598-022-10358-x
328. Gajewicz A, Puzyn T, Odziomek K, Urbaszek P, Haase A, Riebeling C, et al. Decision tree models to classify nanomaterials according to the DF4nanoGrouping scheme. Nanotoxicology [Internet]. Taylor & Francis; 2018 [cited 2025 July 8];12:1–17. https://doi.org/10.1080/17435390.2017.1415388
329. Bilgi E, Karakus CO. Machine learning-assisted prediction of the toxicity of silver nanoparticles: a meta-analysis. J Nanopart Res [Internet]. 2023 [cited 2025 July 8];25:157. https://doi.org/10.1007/s11051-023-05806-2
330. Fresch B, Hiluf D, Collini E, Levine RD, Remacle F. Molecular decision trees realized by ultrafast electronic spectroscopy. Proceedings of the National Academy of Sciences [Internet]. Proceedings of the National Academy of Sciences; 2013 [cited 2025 July 8];110:17183–8. https://doi.org/10.1073/pnas.1314978110
331. Qin W, Henneberger L, Glüge J, König M, Escher BI. Baseline Toxicity Model to Identify the Specific and Nonspecific Effects of Per- and Polyfluoroalkyl Substances in Cell-Based Bioassays. Environ Sci Technol [Internet]. American Chemical Society; 2024 [cited 2025 July 8];58:5727–38. https://doi.org/10.1021/acs.est.3c09950
332. Tweed CD, Crook AM, Amukoye EI, Dawson R, Diacon AH, Hanekom M, et al. Toxicity associated with tuberculosis chemotherapy in the REMoxTB study. BMC Infectious Diseases [Internet]. 2018 [cited 2025 July 8];18:317. https://doi.org/10.1186/s12879-018-3230-6
333. Shipe ME, Deppen SA, Farjah F, Grogan EL. Developing prediction models for clinical use using logistic regression: an overview. J Thorac Dis [Internet]. 2019 [cited 2025 July 8];11:S574–84. https://doi.org/10.21037/jtd.2019.01.25
334. Halvari T, Nurminen JK, Mikkonen T. Testing the Robustness of AutoML Systems. Electron Proc Theor Comput Sci [Internet]. 2020 [cited 2025 July 8];319:103–16. https://doi.org/10.4204/EPTCS.319.8
335. Ribeiro PH, Orzechowski P, Wagenaar J, Moore JH. Benchmarking AutoML algorithms on a collection of synthetic classification problems [Internet]. arXiv; 2023 [cited 2025 July 8]. https://doi.org/10.48550/arXiv.2212.02704
336. Alaiad A, Migdady A, Al-Khatib RM, Alzoubi O, Zitar RA, Abualigah L. Autokeras Approach: A Robust Automated Deep Learning Network for Diagnosis Disease Cases in Medical Images. Journal of Imaging [Internet]. Multidisciplinary Digital Publishing Institute; 2023 [cited 2025 July 8];9:64. https://doi.org/10.3390/jimaging9030064
337. Lin R-H, Lin P, Wang C-C, Tung C-W. A novel multitask learning algorithm for tasks with distinct chemical space: zebrafish toxicity prediction as an example. Journal of Cheminformatics [Internet]. 2024 [cited 2025 July 8];16:91. https://doi.org/10.1186/s13321-024-00891-4
338. Nelatoori KB, Kommanti HB. Multi-task learning for toxic comment classification and rationale extraction. J Intell Inf Syst [Internet]. 2023 [cited 2025 July 8];60:495–519. https://doi.org/10.1007/s10844-022-00726-4
339. Zhao W, Li J, Li H. A multi-task learning approach for chemical process abnormity locations and fault classifications. Chemometrics and Intelligent Laboratory Systems [Internet]. 2023 [cited 2025 July 8];233:104719. https://doi.org/10.1016/j.chemolab.2022.104719

340. Du B-X, Xu Y, Yiu S-M, Yu H, Shi J-Y. ADMET property prediction via multi-task graph learning under adaptive auxiliary task selection. iScience [Internet]. 2023 [cited 2025 July 8];26:108285. https://doi.org/10.1016/j.isci.2023.108285
341. Fuadah YN, Qauli AI, Pramudito MA, Marcellinus A, Hanum UL, Lim KM. A stacking ensemble machine learning model for evaluating cardiac toxicity of drugs based on in silico biomarkers. CPT Pharmacometrics Syst Pharmacol. 2024;13:2159–70. https://doi.org/10.1002/psp4.13229
342. Mohammed A, Kora R. A comprehensive review on ensemble deep learning: Opportunities and challenges. Journal of King Saud University - Computer and Information Sciences [Internet]. 2023 [cited 2025 July 8];35:757–74. https://doi.org/10.1016/j.jksuci.2023.01.014
343. Rawat D, Bajaj R, Manchanda R, Mehta A, Paramasivam P, Bhagat SK, et al. A robust and statistical analyzed predictive model for drug toxicity using machine learning. Sci Rep [Internet]. Nature Publishing Group; 2025 [cited 2025 July 8];15:17993. https://doi.org/10.1038/s41598-025-02333-z
344. Ijcsis J of CS. T-Ensemble Approach for Drug Toxicity Prediction. [cited 2025 July 8]; https://www.academia.edu/31932149/T_Ensemble_Approach_for_Drug_Toxicity_Prediction. Accessed 8 July 2025
345. Li C, Guan N, Zhang H. Anticancer drug synergy prediction based on CatBoost. PeerJ Comput Sci [Internet]. 2025 [cited 2025 July 8];11:e2829. https://doi.org/10.7717/peerj-cs.2829
346. Ji X, Tong W, Liu Z, Shi T. Five-Feature Model for Developing the Classifier for Synergistic vs. Antagonistic Drug Combinations Built by XGBoost. Front Genet [Internet]. Frontiers; 2019 [cited 2025 July 8];10. https://doi.org/10.3389/fgene.2019.00600
347. R S, Ayachit SS, Patil V, Singh A. Competitive Analysis of the Top Gradient Boosting Machine Learning Algorithms. 2020 2nd International Conference on Advances in Computing, Communication Control and Networking (ICACCCN) [Internet]. 2020 [cited 2025 July 8]. p. 191–6. https://doi.org/10.1109/ICACCCN51052.2020.9362840
348. Wu G, Jiang J, Liu X. A Practical Contrastive Learning Framework for Single-Image Super-Resolution. IEEE Transactions on Neural Networks and Learning Systems [Internet]. 2024 [cited 2025 July 8];35:15834–45. https://doi.org/10.1109/TNNLS.2023.3290038
349. Chen T, Kornblith S, Norouzi M, Hinton G. A Simple Framework for Contrastive Learning of Visual Representations [Internet]. arXiv; 2020 [cited 2025 July 8]. https://doi.org/10.48550/arXiv.2002.05709
350. Yang H, Li J. SimCLIC: A Simple Framework for Contrastive Learning of Image Classification. Journal of Systems Science and Information [Internet]. 2023 [cited 2025 July 8];11:204–18. https://doi.org/10.21078/JSSI-2023-204-15
351. Moukafih Y, Sbihi N, Ghogho M, Smaili K. SuperConText: Supervised Contrastive Learning Framework for Textual Representations. IEEE Access [Internet]. 2023 [cited 2025 July 8];11:16820–30. https://doi.org/10.1109/ACCESS.2023.3241490
352. Duan Y, Bao H, Bai G, Wei Y, Xue K, You Z, et al. Learning to Diagnose: Meta-Learning for Efficient Adaptation in Few-Shot AIOps Scenarios. Electronics [Internet]. Multidisciplinary Digital Publishing Institute; 2024 [cited 2025 July 9];13:2102. https://doi.org/10.3390/electronics13112102
353. Gull S, Kim J. Metric-Based Meta-Learning Approach for Few-Shot Classification of Brain Tumors Using Magnetic Resonance Images. Electronics [Internet]. Multidisciplinary Digital Publishing Institute; 2025 [cited 2025 July 9];14:1863. https://doi.org/10.3390/electronics14091863

354. Yang C, Chen X, Huang J, An Y, Huang Z, Sun Y. A few-shot link prediction framework to drug repurposing using multi-level attention network. Comput Biol Med. 2024;170:107936. https://doi.org/10.1016/j.compbiomed.2024.107936
355. Vella D, Ebejer J-P. Few-Shot Learning for Low-Data Drug Discovery. J Chem Inf Model [Internet]. American Chemical Society; 2023 [cited 2025 July 9];63:27–42. https://doi.org/10.1021/acs.jcim.2c00779
356. Jing Y, Bian Y, Hu Z, Wang L, Xie X-QS. Deep Learning for Drug Design: an Artificial Intelligence Paradigm for Drug Discovery in the Big Data Era. AAPS J [Internet]. 2018 [cited 2025 July 9];20:58. https://doi.org/10.1208/s12248-018-0210-0
357. Piroozmand F, Mohammadipanah F, Sajedi H. Spectrum of deep learning algorithms in drug discovery. Chemical Biology & Drug Design [Internet]. 2020 [cited 2025 July 9];96:886–901. https://doi.org/10.1111/cbdd.13674
358. Emmert-Streib F, Yang Z, Feng H, Tripathi S, Dehmer M. An Introductory Review of Deep Learning for Prediction Models With Big Data. Front Artif Intell [Internet]. Frontiers; 2020 [cited 2025 July 9];3. https://doi.org/10.3389/frai.2020.00004
359. Jaiswal AK, Tiwari P, Garg S, Hossain MS. Entity-aware capsule network for multi-class classification of big data: A deep learning approach. Future Generation Computer Systems [Internet]. 2021 [cited 2025 July 8];117:1–11. https://doi.org/10.1016/j.future.2020.11.012
360. Wang N, Yang A, Cui Z, Ding Y, Xue Y, Su Y. Capsule Attention Network for Hyperspectral Image Classification. Remote Sensing [Internet]. Multidisciplinary Digital Publishing Institute; 2024 [cited 2025 July 8];16:4001. https://doi.org/10.3390/rs16214001
361. Lian Y, Chen M. CA-CGNet: Component-Aware Capsule Graph Neural Network for Non-Rigid Shape Correspondence. Applied Sciences [Internet]. Multidisciplinary Digital Publishing Institute; 2023 [cited 2025 July 8];13:3261. https://doi.org/10.3390/app13053261
362. Chattoraj S, Chakraborty A, Gupta A, Vishwakarma Y, Vishwakarma K, Aparajeeta J. Deep Phenotypic Cell Classification using Capsule Neural Network. Annu Int Conf IEEE Eng Med Biol Soc. 2021;2021:4031–6. https://doi.org/10.1109/EMBC46164.2021.9629862
363. Lu J, Deng K, Zhang X, Liu G, Guan Y. Neural-ODE for pharmacokinetics modeling and its advantage to alternative machine learning models in predicting new dosing regimens. iScience. 2021;24:102804. https://doi.org/10.1016/j.isci.2021.102804
364. Giacometti T, Rocchi E, Cojutti PG, Magnani F, Remondini D, Pea F, et al. Leveraging Neural ODEs for Population Pharmacokinetics of Dalbavancin in Sparse Clinical Data. Entropy [Internet]. Multidisciplinary Digital Publishing Institute; 2025 [cited 2025 July 8];27:602. https://doi.org/10.3390/e27060602
365. Owoyele O, Pal P. ChemNODE: A neural ordinary differential equations framework for efficient chemical kinetic solvers. Energy and AI [Internet]. 2022 [cited 2025 July 8];7:100118. https://doi.org/10.1016/j.egyai.2021.100118
366. Rodrigues JA. Using Physics-Informed Neural Networks (PINNs) for Tumor Cell Growth Modeling. Mathematics [Internet]. Multidisciplinary Digital Publishing Institute; 2024 [cited 2025 July 8];12:1195. https://doi.org/10.3390/math12081195
367. Taghizadeh E, Byrne HM, Wood BD. Explicit physics-informed neural networks for nonlinear closure: The case of transport in tissues. Journal of Computational Physics [Internet]. 2022 [cited 2025 July 8];449:110781. https://doi.org/10.1016/j.jcp.2021.110781
368. Moradi Kashkooli F, Kolios MC. Multi-Scale and Multi-Physics Models of the Transport of Therapeutic/Diagnostic Cancer Agents. Cancers (Basel) [Internet]. 2023 [cited 2025 July 8];15:5850. https://doi.org/10.3390/cancers15245850

369. Murari K, Roul P, Sundar S. Physics informed neural network for forward and inverse modeling of low grade brain tumors [Internet]. arXiv; 2025 [cited 2025 July 8]. https://doi.org/10.48550/arXiv.2504.07058
370. Sheller MJ, Edwards B, Reina GA, Martin J, Pati S, Kotrotsou A, et al. Federated learning in medicine: facilitating multi-institutional collaborations without sharing patient data. Sci Rep [Internet]. Nature Publishing Group; 2020 [cited 2025 July 8];10:12598. https://doi.org/10.1038/s41598-020-69250-1
371. Du J, Li W, Liu P, Vong C-M, You Y, Lei B, et al. Federated learning using model projection for multi-center disease diagnosis with non-IID data. Neural Networks [Internet]. 2024 [cited 2025 July 8];178:106409. https://doi.org/10.1016/j.neunet.2024.106409
372. Ju L, Hellander A, Spjuth O. Federated learning for predicting compound mechanism of action based on image-data from cell painting. Artificial Intelligence in the Life Sciences [Internet]. 2024 [cited 2025 July 8];5:100098. https://doi.org/10.1016/j.ailsci.2024.100098
373. Teo ZL, Jin L, Li S, Miao D, Zhang X, Ng WY, et al. Federated machine learning in healthcare: A systematic review on clinical applications and technical architecture. Cell Rep Med [Internet]. 2024 [cited 2025 July 8];5:101419. https://doi.org/10.1016/j.xcrm.2024.101419
374. Hermann M, Kwak H, Ruf B, Weinmann M. Leveraging Neural Radiance Fields for Large-Scale 3D Reconstruction from Aerial Imagery. Remote Sensing [Internet]. Multidisciplinary Digital Publishing Institute; 2024 [cited 2025 July 8];16:4655. https://doi.org/10.3390/rs16244655
375. Khojasteh SB, Fuentes-Jimenez D, Pizarro D, Espinel Y, Bartoli A. MIS-NeRF: neural radiance fields in minimally-invasive surgery. Int J CARS [Internet]. 2025 [cited 2025 July 8];20:1481–90. https://doi.org/10.1007/s11548-025-03429-7
376. Liu J, Shi Y, Huang D, Qu J. Neural Radiance Fields for High-Fidelity Soft Tissue Reconstruction in Endoscopy. Sensors [Internet]. Multidisciplinary Digital Publishing Institute; 2025 [cited 2025 July 8];25:565. https://doi.org/10.3390/s25020565
377. Chen S, Peng M, Li Y, Ju B-F, Bao H, Chen Y-L, et al. Multi-view neural 3D reconstruction of micro- and nanostructures with atomic force microscopy. Commun Eng [Internet]. Nature Publishing Group; 2024 [cited 2025 July 8];3:131. https://doi.org/10.1038/s44172-024-00270-9
378. Jain U, Wilson A, Gulshan V. Multimodal contrastive learning for remote sensing tasks [Internet]. arXiv; 2022 [cited 2025 July 8]. https://doi.org/10.48550/arXiv.2209.02329
379. Xia W, Zhang C, Weng C, Yu M, Yu D. Self-Supervised Text-Independent Speaker Verification Using Prototypical Momentum Contrastive Learning. ICASSP 2021 - 2021 IEEE International Conference on Acoustics, Speech and Signal Processing (ICASSP) [Internet]. 2021 [cited 2025 July 8]. p. 6723–7. https://doi.org/10.1109/ICASSP39728.2021.9414973
380. Alakhdar A, Poczos B, Washburn N. Diffusion Models in De Novo Drug Design. J Chem Inf Model [Internet]. American Chemical Society; 2024 [cited 2025 July 8];64:7238–56. https://doi.org/10.1021/acs.jcim.4c01107
381. Sako M, Yasuo N, Sekijima M. DiffInt: A Diffusion Model for Structure-Based Drug Design with Explicit Hydrogen Bond Interaction Guidance. J Chem Inf Model [Internet]. American Chemical Society; 2025 [cited 2025 July 8];65:71–82. https://doi.org/10.1021/acs.jcim.4c01385
382. Watson JL, Juergens D, Bennett NR, Trippe BL, Yim J, Eisenach HE, et al. De novo design of protein structure and function with RFdiffusion. Nature [Internet]. Nature Publishing Group; 2023 [cited 2025 July 8];620:1089–100. https://doi.org/10.1038/s41586-023-06415-8
383. Cao H, Tan C, Gao Z, Xu Y, Chen G, Heng P-A, et al. A Survey on Generative Diffusion Models. IEEE Transactions on Knowledge and Data Engineering [Internet]. IEEE Computer Society; 2024 [cited 2025 July 8];36:2814–30. https://doi.org/10.1109/TKDE.2024.3361474

384. Batzner S, Musaelian A, Sun L, Geiger M, Mailoa JP, Kornbluth M, et al. E(3)-equivariant graph neural networks for data-efficient and accurate interatomic potentials. Nat Commun [Internet]. Nature Publishing Group; 2022 [cited 2025 July 9];13:2453. https://doi.org/10.1038/s41467-022-29939-5
385. Unke O, Bogojeski M, Gastegger M, Geiger M, Smidt T, Müller K-R. SE(3)-equivariant prediction of molecular wavefunctions and electronic densities. Advances in Neural Information Processing Systems [Internet]. Curran Associates, Inc.; 2021 [cited 2025 July 9]. p. 14434–47. https://proceedings.neurips.cc/paper/2021/hash/78f1893678afbeaa90b1fa01b9cfb860-Abstract.html. Accessed 9 July 2025
386. Gong X, Li H, Zou N, Xu R, Duan W, Xu Y. General framework for E(3)-equivariant neural network representation of density functional theory Hamiltonian. Nat Commun [Internet]. Nature Publishing Group; 2023 [cited 2025 July 9];14:2848. https://doi.org/10.1038/s41467-023-38468-8
387. Ahmad W, Tayara H, Chong KT. Attention-Based Graph Neural Network for Molecular Solubility Prediction. ACS Omega [Internet]. American Chemical Society; 2023 [cited 2025 July 9];8:3236–44. https://doi.org/10.1021/acsomega.2c06702
388. Vrahatis AG, Lazaros K, Kotsiantis S. Graph Attention Networks: A Comprehensive Review of Methods and Applications. Future Internet [Internet]. Multidisciplinary Digital Publishing Institute; 2024 [cited 2025 July 9];16:318. https://doi.org/10.3390/fi16090318
389. Shen J, Ain QT, Liu Y, Liang B, Qiang X, Kou Z. GTAT: empowering graph neural networks with cross attention. Sci Rep [Internet]. Nature Publishing Group; 2025 [cited 2025 July 9];15:4760. https://doi.org/10.1038/s41598-025-88993-3
390. Lim J, Ryu S, Park K, Choe YJ, Ham J, Kim WY. Predicting Drug–Target Interaction Using a Novel Graph Neural Network with 3D Structure-Embedded Graph Representation. J Chem Inf Model [Internet]. American Chemical Society; 2019 [cited 2025 July 9];59:3981–8. https://doi.org/10.1021/acs.jcim.9b00387
391. Chatterjee T, Shaw AD, Friswell MI, Khodaparast HH. Sparse Bayesian machine learning for the interpretable identification of nonlinear structural dynamics: Towards the experimental data-driven discovery of a quasi zero stiffness device. Mechanical Systems and Signal Processing [Internet]. 2023 [cited 2025 July 9];205:110858. https://doi.org/10.1016/j.ymssp.2023.110858
392. Chatterjee T, Chowdhury R. Refined sparse Bayesian learning configuration for stochastic response analysis. Probabilistic Engineering Mechanics [Internet]. 2018 [cited 2025 July 9];52:15–27. https://doi.org/10.1016/j.probengmech.2018.02.001
393. Tripura T, Chakraborty S. A sparse Bayesian framework for discovering interpretable nonlinear stochastic dynamical systems with Gaussian white noise. Mechanical Systems and Signal Processing [Internet]. 2023 [cited 2025 July 9];187:109939. https://doi.org/10.1016/j.ymssp.2022.109939
394. Zhu Y, Zhao H. Hypernetwork Representation Learning with the Set Constraint. Applied Sciences [Internet]. Multidisciplinary Digital Publishing Institute; 2022 [cited 2025 July 9];12:2650. https://doi.org/10.3390/app12052650
395. Ren Y, Ma H, Liu S, Wang K. Hypernetwork Link Prediction Method Based on Fusion of Topology and Attribute Features. Entropy [Internet]. Multidisciplinary Digital Publishing Institute; 2023 [cited 2025 July 9];25:89. https://doi.org/10.3390/e25010089
396. Cho W, Lee K, Rim D, Park N. Hypernetwork-based Meta-Learning for Low-Rank Physics-Informed Neural Networks. 2023 [cited 2025 July 9]. https://openreview.net/forum?id=dzqKAM2sKa. Accessed 9 July 2025

397. Beck J, Jackson MT, Vuorio R, Whiteson S. Hypernetworks in Meta-Reinforcement Learning [Internet]. arXiv; 2022 [cited 2025 July 9]. https://doi.org/10.48550/arXiv.2210.11348
398. Scius-Bertrand A, Jungo M, Vögtlin L, Spat J-M, Fischer A. Zero-Shot Prompting and Few-Shot Fine-Tuning: Revisiting Document Image Classification Using Large Language Models. 2025 [cited 2025 July 8]. p. 152–66. https://doi.org/10.1007/978-3-031-78495-8_10
399. Patil R, Gudivada V. A Review of Current Trends, Techniques, and Challenges in Large Language Models (LLMs). Applied Sciences [Internet]. Multidisciplinary Digital Publishing Institute; 2024 [cited 2025 July 8];14:2074. https://doi.org/10.3390/app14052074
400. Seputis D, Mihailov S, Chatterjee S, Xiao Z. Multi-Modal Adapter for Vision-Language Models [Internet]. arXiv; 2024 [cited 2025 July 9]. https://doi.org/10.48550/arXiv.2409.02958
401. Yang F, Zhu Y, Li X, Zhan Y, Zhao H, Zheng S, et al. FOCUS: Unified Vision-Language Modeling for Interactive Editing Driven by Referential Segmentation [Internet]. arXiv; 2025 [cited 2025 July 9]. https://doi.org/10.48550/arXiv.2506.16806
402. Li X, Li J, Li F, Zhu L, Yang Y, Shen HT. Generalizing vision-language models to novel domains: A comprehensive survey [Internet]. arXiv; 2025 [cited 2025 July 9]. https://doi.org/10.48550/arXiv.2506.18504
403. Zhang J, Cai K, Fan Y, Wang J, Wang K. CF-VLM:CounterFactual Vision-Language Fine-tuning [Internet]. arXiv; 2025 [cited 2025 July 9]. https://doi.org/10.48550/arXiv.2506.17267
404. Zhang J, Liu Z, Wang Y, Li Y. SubGDiff: A Subgraph Diffusion Model to Improve Molecular Representation Learning [Internet]. arXiv; 2024 [cited 2025 July 9]. https://doi.org/10.48550/arXiv.2405.05665
405. Chen H, Xu C, Zheng L, Zhang Q, Lin X. Diffusion-based Graph Generative Methods [Internet]. arXiv; 2024 [cited 2025 July 9]. https://doi.org/10.48550/arXiv.2401.15617
406. Yang N, Wu H, Zeng K, Li Y, Bao S, Yan J. Molecule generation for drug design: A graph learning perspective. Fundamental Research [Internet]. 2024 [cited 2025 July 9]; https://doi.org/10.1016/j.fmre.2024.11.027
407. Wen L, Tang X, Ouyang M, Shen X, Yang J, Zhu D, et al. Hyperbolic Graph Diffusion Model [Internet]. arXiv; 2024 [cited 2025 July 9]. https://doi.org/10.48550/arXiv.2306.07618
408. Lee J, Yoon W, Kim S, Kim D, Kim S, So CH, et al. BioBERT: a pre-trained biomedical language representation model for biomedical text mining. Bioinformatics [Internet]. 2020 [cited 2025 July 9];36:1234–40. https://doi.org/10.1093/bioinformatics/btz682
409. Zhao S, Su C, Lu Z, Wang F. Recent advances in biomedical literature mining. Brief Bioinform [Internet]. 2020 [cited 2025 July 9];22:bbaa057. https://doi.org/10.1093/bib/bbaa057
410. Sun C, Yang Z, Wang L, Zhang Y, Lin H, Wang J. Biomedical named entity recognition using BERT in the machine reading comprehension framework. Journal of Biomedical Informatics [Internet]. 2021 [cited 2025 July 9];118:103799. https://doi.org/10.1016/j.jbi.2021.103799
411. Moreau E, Hardiman O, Heverin M, O'Sullivan D. Mining impactful discoveries from the biomedical literature. BMC Bioinformatics [Internet]. 2024 [cited 2025 July 9];25:303. https://doi.org/10.1186/s12859-024-05881-9
412. Beltagy I, Lo K, Cohan A. SciBERT: A Pretrained Language Model for Scientific Text [Internet]. arXiv; 2019 [cited 2025 July 9]. https://doi.org/10.48550/arXiv.1903.10676
413. Seth P, Rathore Y, Singh NK, Chitroda C, Sankarapu VK. xai_evals : A Framework for Evaluating Post-Hoc Local Explanation Methods [Internet]. arXiv; 2025 [cited 2025 July 8]. https://doi.org/10.48550/arXiv.2502.03014
414. Dehdarirad T. Evaluating explainability in language classification models: A unified framework incorporating feature attribution methods and key factors affecting faithfulness. Data

and Information Management [Internet]. 2025 [cited 2025 July 8];100101. https://doi.org/10.1016/j.dim.2025.100101

415. Hooshyar D, Yang Y. Problems With SHAP and LIME in Interpretable AI for Education: A Comparative Study of Post-Hoc Explanations and Neural-Symbolic Rule Extraction. IEEE Access [Internet]. 2024 [cited 2025 July 8];12:137472–90. https://doi.org/10.1109/ACCESS.2024.3463948

416. Salih AM, Raisi-Estabragh Z, Galazzo IB, Radeva P, Petersen SE, Lekadir K, et al. A Perspective on Explainable Artificial Intelligence Methods: SHAP and LIME. Advanced Intelligent Systems [Internet]. 2025 [cited 2025 July 8];7:2400304. https://doi.org/10.1002/aisy.202400304
